# Energetic and Economic Aspects of Rebound, Part I: Foundations of a Rigorous Analytical Framework

**DOI:** 10.1177/01956574251331969

**Published:** 2025-07-07

**Authors:** Matthew Kuperus Heun, Gregor Semieniuk, Paul E. Brockway

**Affiliations:** 1 Engineering Department, Calvin University, Grand Rapids, MI, USA; 2 Sustainability Research Institute, School of Earth and Environment, University of Leeds, Leeds, UK; 3 Faculty of Economic and Management Science, School for Public Leadership, Stellenbosch University, Stellenbosch, South Africa; 4 School of Public Policy & Department of Economics, University of Massachusetts Amherst, Amherst, MA, USA; 5 World Bank, Washington, DC, USA

**Keywords:** energy efficiency, energy rebound, energy services, microeconomic rebound, substitution and income effects, macroeconomic rebound

## Abstract

Widespread implementation of energy efficiency is a key greenhouse gas emissions mitigation measure, but rebound can “take back” energy savings. However, the absence of solid analytical foundations hinders empirical determination of the size of rebound. A new clarity is needed, one that involves both economics and energy analysis. In this paper (Part I), we advance foundations of a rigorous analytical framework for consumer-sided rebound that starts at the microeconomic level and is approachable for both energy analysts and economists. We develop foundations of a framework that (i) clarifies the energy, expenditure, and consumption aspects of rebound, (ii) combines embodied energy with operations, maintenance, and disposal effects (under a new “emplacement effect”), and (iii) provides the first operationalized link between microeconomic and macroeconomic levels. The framework enables determination of the effects of non-marginal energy service price decreases, satiated demand for the energy service, and reduced economy-wide energy demand.

**JEL Classification**: O13, Q40, Q43

## 1. Introduction

Energy efficiency is often considered to be the most important means of reducing energy consumption and CO_2_ emissions (International Energy Agency 2017, 139: Fig. 3.15). But energy rebound makes energy efficiency less effective at decreasing energy consumption by taking back (or reversing, in the case of “backfire”) energy savings expected from energy efficiency improvements (Sorrell 2009). As such, energy rebound is a threat to a low-carbon future (Brockway et al. 2017; van den Bergh 2017).

Recent evidence shows that rebound is both larger than commonly assumed (Stern 2020) and mostly missing from large energy and climate models (Brockway et al. 2021). Thus, rebound could be an important reason why energy consumption and carbon emissions have never been absolutely decoupled from economic growth (Brockway et al. 2021; Haberl et al. 2020).

### 1.1. A Short History of Rebound

Famously, the roots of energy rebound trace back to Jevons who said “*[i]t is wholly a confusion of ideas to suppose that the economical use of fuel is equivalent to a diminished consumption. The very contrary is the truth*” (Jevons 1865, 103, emphasis in original). Less famously, the origins of rebound extend further backward from Jevons to Williams (1840) and Parkes who wrote “[t]he economy of fuel is the secret of the economy of the steam-engine; it is the fountain of its power, and the adopted measure of its effects. Whatever, therefore, conduces to increase the efficiency of coal, and to diminish the cost of its use, directly tends to augment the value of the steam-engine, and to enlarge the field of its operations” (Parkes 1838, 161). For nearly 200 years, then, it has been understood that efficiency gains may be taken back or, paradoxically, cause *growth* in energy consumption, as Jevons suggested.

The oil crises of the 1970s shone a light back onto energy efficiency, and research into rebound appeared late in the decade (Madlener and Turner 2016; Saunders et al. 2021). A modern debate over the magnitude of energy rebound commenced. On one side, scholars including Brookes (1979, 1990) and Khazzoom (1980) suggested rebound could be large. Others, including Lovins (1988) and Grubb (1990, 1992), claimed rebound was likely to be small. Debate over the size of energy rebound continues today. Advocates of small rebound (less than, say, 50%), suggest “the rebound effect is overplayed” (Gillingham et al. 2013, 475), while others claim (i) that the evidence for large rebound (greater than 50%) is growing (Berner et al. 2022; Saunders 2015) and (ii) that rebound will reduce the effectiveness of energy efficiency to decrease carbon emissions (van den Bergh 2017).

### 1.2. Absence of Solid Analytical Foundations

Turner contends that the lack of consensus on the magnitude of energy rebound in the modern empirical literature is caused by “a rush to empirical estimation in the absence of solid analytical foundations” (Turner 2013, 25). Progress has been made recently on how price changes affect economy-wide rebound in general equilibrium frameworks (Blackburn and Moreno-Cruz 2020; Fullerton and Ta 2020; Lemoine 2020). And arguments from microeconomics (i.e., at sectoral and individual level) have been used from the outset of the modern debate (e.g., Greening, Greene and Difiglio 2000; Khazzoom 1980), and Borenstein (2015) and Chan and Gillingham (2015) made further progress toward solidifying the microeconomic analytical foundations.

Rebound involves simultaneous changes in energy, expenditure, and consumption aspects—keeping an overview of all aspects is difficult, with no approach to our knowledge documenting all changes in a straightforward and consistent manner. For instance, while the microeconomic categories of substitution and income effects provide analytical clarity about how behavior changes affect energy service consumption, it has been unclear how they could be used for precise numerical rebound calculations. Where previous numerical calculations were made, they tended to approximate the substitution effect from other goods to the cheaper energy service, without maintaining constant utility for the device user. They also used constant price elasticities for non-marginal efficiency improvements, even though constant price elasticities typically provide only approximations of substitution and income effects for small efficiency changes. Further, previous analytical studies have stressed the importance of the cost of buying an upgraded device as well as the energy embodied in the device. Yet, there is no clearly formulated approach for how to incorporate these cost and energy components into rebound calculations. Finally, while recent general equilibrium rebound modeling has led to important insights about the effects of changing prices, dynamic aspects of a macroeconomic rebound have been neglected by these approaches.

In the absence of solid analytical foundations, the wide variety of rebound calculation approaches contributes to a wide range of rebound values, giving the appearance of uncertainty and leading some energy and climate modelers to either (i) use questionable rebound values or (ii) ignore rebound altogether. Insufficient inclusion of rebound in energy and climate models could lead to overly optimistic projections of the capability of energy efficiency to reduce carbon emissions (Brockway et al. 2021). We suggest that improving the conceptual foundations of rebound and solidifying the analytical frameworks will (i) help generate more robust estimates of rebound, (ii) lead to better rebound calculations in energy and climate models, and (iii) provide improved evidence for policymaking around energy efficiency.

But why is there an “absence of solid analytical foundations?” We propose that development of solid analytical frameworks for rebound is hampered by the fact that rebound is a decidedly interdisciplinary topic, involving both economics and energy analysis. Birol and Keppler (2000, 458) note that “different implicit and explicit assumptions of different research communities (‘economists’, ‘engineers’) … have in the past led to vastly differing points of view.”^1^ Turner states that “[d]ifferent definitions of energy efficiency will be appropriate in different circumstances. However, … it is often not clear what different authors mean by energy efficiency” (Turner 2013, 237–8). If authors from the two disciplines cannot even agree on the key terms, it is unsurprising that analytical foundations have not yet been fully elucidated. To fully understand rebound, economists need to have an energy analyst’s understanding of energy, and energy analysts need to have an economist’s understanding of finance and human behavior.^2^ Developing the knowledge and skills required to assess and calculate, let alone mitigate, rebound effects is a tall order, indeed.

### 1.3. New Clarity Is Needed

We contend that new clarity is needed. Specifically, a description of rebound that is (i) consistent across energy, expenditure, and consumption aspects, (ii) technically rigorous, and (iii) approachable from both disciplines (economics and energy analysis) will be a good starting point toward that clarity. In other words, the finance and human behavior aspects of rebound need to be presented in ways energy analysts can understand. And the energy aspects of rebound need to be presented in ways economists can understand.

Summarizing, we surmise that development of effective carbon reduction policies has been hampered, in part, by the fact that rebound is not sufficiently included in energy and climate models. We suspect that one reason rebound is not sufficiently included is the lack of consensus on rebound calculation methods and, hence, rebound magnitude. Building upon Turner (2013), we contend that lack of consensus on rebound magnitude is a symptom of the absence of solid analytical foundations for rebound. We posit that developing solid analytical frameworks is difficult because energy rebound is an inherently interdisciplinary topic. We believe that providing a detailed explication of a rigorous analytical framework for energy rebound, which is approachable by both energy analysts and economists alike, will go some way toward providing additional clarity in the field.

### 1.4. Objective, Contributions, and Structure

The *objective* of this paper is to help advance clarity in the field of energy rebound by supporting the development of a rigorous analytical framework, one that (i) starts at the microeconomics of rebound (building especially upon Borenstein 2015) and (ii) is approachable for both energy analysts and economists.^3^ We strive to keep the framework as simple as possible and limit our attention to a model of consumer demand for energy services, while demonstrating that the approach is transferable to a producer model with few modifications.

The key *contributions* of this paper are (i) a novel and clear explication of interrelated energy, expenditure, and consumption aspects of energy rebound, (ii) development of a rebound analysis framework that combines embodied energy effects, operations, maintenance, and disposal rebound effects, and exact expressions for substitution and income rebound effects under non-marginal energy efficiency increases and (by implication) non-marginal energy service price decreases, (iii) an operationalized link between rebound effects on microeconomic and macroeconomic levels, and (iv) development of an extension of the framework to an energy price rebound effect.

The remainder of this paper is *structured* as follows. Section 2 describes the rebound analysis framework. Section 3 discusses this framework relative to previous frameworks and provides an initial assessment of an energy price effect. Section 4 concludes. Results from the application of our framework to energy efficiency upgrades to a car and an electric lamp can be found in Heun et al. (2025).

## 2. Methods: Development of the Framework

In this section, we develop an energy rebound framework for an individual consumer who upgrades the energy efficiency of a single device (concisely, “the framework,”“this framework,” or “our framework”). We endeavor to help advance clarity in the field of energy rebound by providing sufficient detail to assist energy analysts to understand the economics and economists to understand the energy analysis.

### 2.1. Rebound Typology

Table 1 shows our typology of rebound effects. We follow others, including Jenkins, Nordhaus and Shellenberger (2011) and Walnum, Aall and Løkke (2014), in identifying and including both direct and indirect rebound effects, which occur at (direct) and beyond (indirect) the level of the device and its user. Again following others, such as Gillingham, Rapson and Wagner (2016), we distinguish between rebound effects at the microeconomic and macroeconomic levels.

**Table 1.** Rebound Typology for Our Framework.

Rebound level	Direct rebound ( $Re_{\mathrm{dir}}$ )	Indirect rebound ( $Re_{\mathrm{indir}}$ )
*Microeconomic rebound* ( $Re_{\mathrm{micro}}$ )	*Emplacement effect* ( $Re_{\mathrm{dempl}}$ )	*Emplacement effect* ( $Re_{\mathrm{iempl}}$ )
These mechanisms occur at the single device/user level within a static economy based on responses to the reduction in implicit price of an energy service.	Accounts for performance of the Energy Efficiency Upgrade (EEU) only. No behavior changes occur. The direct energy effect of emplacement of the EEU is expected device-level energy savings. By definition, there is no rebound from direct emplacement effects ( $Re_{\mathrm{dempl}}\;\equiv \;0$ ).	Differential energy adjustments beyond the usage of the upgraded device, via (i) the embodied energy associated with the manufacturing phase ( $Re_{\mathrm{emb}}$ ) and (ii) the implied energy demand from operations, maintenance, and disposal ( $Re_{\mathrm{OMd}}$ ). $Re_{\mathrm{iempl}}$ can be >0 or <0 , depending on the characteristics of the EEU.
	*Substitution effect* ( $Re_{\mathrm{dsub}}$ )	*Substitution effect* ( $Re_{\mathrm{isub}}$ )
	Increase in energy service consumption due to its lower prices as a result of the EEU. Excludes, by definition, the effects of freed cash (income effects). $Re_{\mathrm{dsub}}\;>\;0$ is typical due to greater consumption of the energy service.	Reduction in other goods consumption due to the relatively higher prices as a result of the EEU. Excludes, by definition, the effects of freed cash (income effects). $Re_{\mathrm{isub}}\;<\;0$ is typical due to reduced consumption of other goods and services.
	*Income effect* ( $Re_{\mathrm{dinc}}$ )	*Income effect* ( $Re_{\mathrm{iinc}}$ )
	Spending of some of the freed cash to obtain more of the energy service. $Re_{\mathrm{dinc}}\;>\;0$ is typical due to increased consumption of the energy service.	Spending of some of the freed cash on other goods and services. $Re_{\mathrm{iinc}}\;>\;0$ is typical due to increased consumption of other goods and services.
*Macroeconomic rebound* ( $Re_{\mathrm{macro}}$ )		*Macroeconomic effect* ( $Re_{\mathrm{macro}}$ )
These mechanisms originate from the dynamic response of the economy to reach a stable equilibrium (between supply and demand for energy services and other goods). These mechanisms combine various short and long run effects.		Increased energy consumption in the broader macroeconomic system, that is, beyond responses at the microeconomic (device/user) level. $Re_{\mathrm{macro}}\;>\;0$ is typical due to sectoral productivity increases (the EEU) causing additional economic growth in the wider economy.

Microeconomic rebound occurs at the level of the single device and its user and in our framework comprises three effects: an emplacement effect, a substitution effect, and an income effect, with direct and indirect partitions for each.

“Emplacement” is a new term we introduce to collect effects associated with installing higher-efficiency devices, including (i) embodied energy of their manufacture (subscript emb ), (ii) operations and maintenance (subscript OM ), and (iii) disposal (subscript d ) activities. Although none of the embodied, operations and maintenance, or disposal effects are new (see Borenstein 2015, 3: footnote 5; 16: footnote 37; Saunders et al. 2021; Sorrell, Dimitropoulos and Sommerville 2009; Sorrell, Gatersleben and Druckman 2020), we separate them from substitution and income microeconomic effects (Table 1) to calculate rebound according to the steps in our framework (see Section 2.5).

The direct rebound effect can be partitioned into a direct emplacement effect, a direct substitution effect, and a direct income effect. At the level of the device, all of the direct rebound effects change the consumption of energy by the device whose efficiency has been upgraded, according to a microeconomic behavioral model of the consumer who responds to the cheaper energy service.

Similarly, the indirect rebound effect can be partitioned into an indirect emplacement effect, an indirect substitution effect, and an indirect income effect. All of the indirect effects change the induced energy consumption beyond the upgraded device, again according to a microeconomic behavioral model. We assume a *partial equilibrium* response to the energy efficiency upgrade (EEU) at the microeconomic level; other prices in the economy ( $p_{g}$ ) remain unchanged in response to the EEU.

In contrast, macroeconomic rebound is a broader, economy-wide response to the single device upgrade. Like other authors, we recognize many macroeconomic rebound effects, even if we don’t later distinguish among them.^4^ At the macroeconomic level, *general equilibrium* effects can occur as prices for all goods and services (even energy) may change in response to the EEU. Further treatment of macroeconomic rebound can be found in Section 2.5.4 of this paper (Part I) and in Section 4.1 of Heun et al. (2025). Discussion of an energy price rebound effect can be seen in Section 3.2 below and in Section 4.6 of Heun et al. (2025).

**Figure 1.**
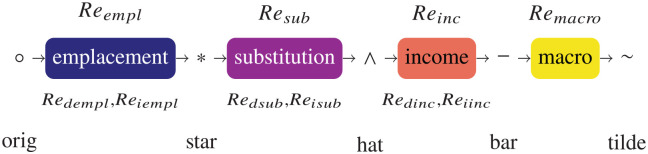
Flowchart of rebound effects and decorations.

Figure 1 shows rebound effects arranged in the left-to-right order of their discussion in this paper. The left-to-right order does not necessarily represent the progression of rebound effects through time. Rebound symbols are shown above each effect ( $Re_{\mathrm{empl}}$ , etc.). Nomenclature for partitions of direct and indirect rebound is shown beneath each effect ( $Re_{\mathrm{dempl}}$ , etc.). Decorations for each stage are shown between rebound effects (○, * etc.). Names for the decorations are given at the bottom of the figure (“orig,”“star,” etc.).^5^

### 2.2. Rebound Relationships

Energy rebound ( Re ) is defined as

(1) Re≡1−actualfinalenergysavingsrateexpectedfinalenergysavingsrate,

where both actual and expected final energy savings rates are in MJ/yr (megajoules per year) and expected positive. The final energy “takeback” rate is defined as the expected final energy savings rate less the actual final energy savings rate.^6^ Rewriting equation (1) with the definition of takeback gives

(2) Re=1−expectedfinalenergysavingsrate-takebackrateexpectedfinalenergysavingsrate.

Simplifying gives

(3) Re=takebackrateexpectedfinalenergysavingsrate.

We define rebound at the final energy^7^ stage of the energy conversion chain, because the final energy stage is the point of energy purchase by the device user. To simplify derivations, we choose not to apply final-to-primary energy multipliers to final energy rates in the numerators and denominators of rebound expressions derived from equations (1) and (3); they divide out anyway.^8^ Henceforth, we drop the adjective “final” from the noun “energy,” unless there is reason to indicate a specific stage of the energy conversion chain.

### 2.3. The Energy Conversion Device and Energy Efficiency Upgrade (EEU)

We assume an energy conversion device (say, a car) that consumes energy (say, gasoline) at a rate ${\overset{\cdot }{E}}^{{}^{\circ}}$ (in MJ/yr). We use “rate” to indicate any quantity measured per unit time, such as a flow of energy per year or a flow of income per year. None of the rates in this paper indicate exponential (%/yr) changes. Rates are identified by a single dot above the symbol, a convention adopted from the engineering literature where, for example, $\overset{\cdot }{x}$ often indicates a velocity in m/s (meters per second) and $\overset{\cdot }{E}$ often indicates an energy flow rate in kW (kilowatts). The overdot is an important notational element in this paper, as it distinguishes between stocks (without overdots) and flows (with overdots). For example, E is a quantity of energy in, say, MJ, while $\overset{\cdot }{E}$ is a rate of energy in, say, MJ/yr. We later annualize capital costs ( $C_{\mathrm{cap}}$ in $), disposal costs ( $C_{d}$ in $), and energy embodied in the device during its production ( $E_{\mathrm{emb}}$ in MJ) to create undiscounted cost rates ( ${\overset{\cdot }{C}}_{\mathrm{cap}}$ and ${\overset{\cdot }{C}}_{d}$ in $/yr) and embodied energy rates ( ${\overset{\cdot }{E}}_{\mathrm{emb}}$ in MJ/yr). (Cost discounting^9^ is captured by the variables $\tau_{\alpha }$ and $\tau_{\omega }$ . See Appendix B.1 for details.)

Energy is available at price $p_{E}$ (in $/MJ). The original energy conversion device provides a rate of energy service ${\overset{\cdot }{q}}_{s}^{{}^{\circ}}$ (in, say, vehicle-km/yr) with final-to-service efficiency $\eta^{{}^{\circ}}$ (in, say, vehicle-km/MJ). An energy efficiency upgrade (EEU) increases final-to-service efficiency such that $\eta^{{}^{\circ}}\;<\;\eta^{*}=\hat{\eta }=\bar{\eta }=\tilde{\eta }$ , as shown in Table B.1. The EEU is not costless, so the upgraded device may be more expensive to purchase than a like-for-like replacement of the original device. We call this increased “capital cost” ( $C_{\mathrm{cap}}^{{}^{\circ}}\;<\;C_{\mathrm{cap}}^{*}$ ). It may also be more costly to operate and maintain (subscript OM ) and dispose (subscript d ) of the upgraded device ( ${\overset{\cdot }{C}}_{\mathrm{OM}}^{{}^{\circ}}\;<\;{\overset{\cdot }{C}}_{\mathrm{OM}}^{*}$ and ${\overset{\cdot }{C}}_{d}^{{}^{\circ}}\;<\;{\overset{\cdot }{C}}_{d}^{*}$ ). However, the opposite may hold, too. As final-to-service efficiency increases ( $\eta^{{}^{\circ}}\;<\;\eta^{*}$ ), the price of the energy service declines ( $p_{s}^{{}^{\circ}}\;>\;p_{s}^{*}$ ). The energy price ( $p_{E}$ ) is assumed exogenous at the microeconomic level ( $p_{E}^{{}^{\circ}}=p_{E}^{*}={\hat{p}}_{E}={\bar{p}}_{E}={\tilde{p}}_{E}$ ), so the energy purchaser (the device user) is a price taker.^10^ Initially, the device user spends income ( $\overset{\cdot }{M}$ ) on energy for the device ( ${\overset{\cdot }{C}}_{s}^{{}^{\circ}}=p_{E}^{{}^{\circ}}{\overset{\cdot }{E}}_{s}^{{}^{\circ}}$ ), annualized capital costs for the device ( $\tau_{\alpha }^{{}^{\circ}}{\overset{\cdot }{C}}_{\mathrm{cap}}^{{}^{\circ}}$ ), annualized costs for operations and maintenance ( ${\overset{\cdot }{C}}_{\mathrm{OM}}^{{}^{\circ}}$ ) and disposal of the device ( $\tau_{\omega }^{{}^{\circ}}{\overset{\cdot }{C}}_{d}^{{}^{\circ}}$ ), and other goods and services ( ${\overset{\cdot }{C}}_{g}^{{}^{\circ}}$ ). The budget constraint for the device user is

(4) $$\overset{\cdot }{M}=\tau_{\alpha }^{{}^{\circ}}{\overset{\cdot }{C}}_{\mathrm{cap}}^{{}^{\circ}}+{\overset{\cdot }{C}}_{s}^{{}^{\circ}}+{\overset{\cdot }{C}}_{\mathrm{OM}}^{{}^{\circ}}+\tau_{\omega }^{{}^{\circ}}{\overset{\cdot }{C}}_{d}^{{}^{\circ}}+{\overset{\cdot }{C}}_{g}^{{}^{\circ}},$$

where $\tau_{\alpha }^{{}^{\circ}}$ and $\tau_{\omega }^{{}^{\circ}}$ account for discounting, and ${\overset{\cdot }{C}}_{\mathrm{cap}}^{{}^{\circ}}$ and ${\overset{\cdot }{C}}_{d}^{{}^{\circ}}$ are undiscounted cost rates given by $C_{\mathrm{cap}}^{{}^{\circ}}/t_{\mathrm{life}}^{{}^{\circ}}$ and $C_{d}^{{}^{\circ}}/t_{\mathrm{life}}^{{}^{\circ}}$ . Note that $\tau_{\alpha }\geq 1$ , and $\tau_{\omega }\leq 1$ ; equalities apply when interest rate ( r ) is zero. (See Appendix B.1 for details on discounting.) After substituting the product of energy price ( $p_{E}$ ) and the rate of energy consumption (given by the ratio of the rate of energy service consumption and efficiency, ${\overset{\cdot }{q}}_{s}/\eta$ ), after substituting the product of price ( $p_{g}$ ) and the rate ( ${\overset{\cdot }{q}}_{g}$ ) of other goods consumption, after substituting ${\overset{\cdot }{C}}_{\mathrm{OMd}}^{{}^{\circ}}\;\equiv \;{\overset{\cdot }{C}}_{\mathrm{OM}}^{{}^{\circ}}+\tau_{\omega }^{{}^{\circ}}{\overset{\cdot }{C}}_{d}^{{}^{\circ}}$ , and after some rearrangement, equation (4) becomes

(5) $$\overset{\cdot }{M}-\tau_{\alpha }^{{}^{\circ}}{\overset{\cdot }{C}}_{\mathrm{cap}}^{{}^{\circ}}-{\overset{\cdot }{C}}_{\mathrm{OMd}}^{{}^{\circ}}=p_{E}^{{}^{\circ}}\frac{{\overset{\cdot }{q}}_{s}^{{}^{\circ}}}{\eta^{{}^{\circ}}}+p_{g}{\overset{\cdot }{q}}_{g}^{{}^{\circ}}\;,$$

which is the usual discounted budget constraint for the microeconomic consumer after subtracting capital, operations and maintenance, and disposal costs.

Later (Sections 2.5.1–2.5.4), we walk through the four rebound effects (emplacement, substitution, income, and macro), deriving rebound expressions for each, but first we show typical energy and cost relationships (Section 2.4).

### 2.4. Typical Energy and Cost Relationships

With the rebound notation of Appendix A, four typical relationships emerge. First, the consumption rate of the energy service ( ${\overset{\cdot }{q}}_{s}$ ) is the product of final-to-service efficiency ( η ) and the rate of energy consumption by the energy conversion device ( ${\overset{\cdot }{E}}_{s}$ ). Typical units for automotive transport and illumination (the examples in Heun et al. (2025)) are shown beneath each equation.^11^

(6) $$\begin{array}{c} {\overset{\cdot }{q}}_{s}=\eta {\overset{\cdot }{E}}_{s} \end{array}$$

$$\begin{array}{c} \left[\mathrm{pass}\cdot \mathrm{km}/\mathrm{yr}]=[\mathrm{pass}\cdot \mathrm{km}/\mathrm{MJ}][\mathrm{MJ}/\mathrm{yr}\right]\\ \left[\mathrm{lm}\cdot \mathrm{hr}/\mathrm{yr}]=[\mathrm{lm}\cdot \mathrm{hr}/\mathrm{MJ}][\mathrm{MJ}/\mathrm{yr}\right] \end{array}$$

Second, the energy service price ( $p_{s}$ ) is the ratio of energy price ( $p_{E}$ ) to the final-to-service efficiency ( η ).

(7) $$p_{s}=\frac{p_{E}}{\eta }$$

$$\begin{array}{c} {}[\$/\mathrm{pass}\cdot \mathrm{km}]=\frac{\left[\frac{\$}{\mathrm{MJ}}\right]}{\left[\mathrm{pass}\cdot \mathrm{km}/\mathrm{MJ}\right]}\\ {}[\$/\mathrm{lm}\cdot \mathrm{hr}]=\frac{\left[\$/\mathrm{MJ}\right]}{\left[\mathrm{lm}\cdot \mathrm{hr}/\mathrm{MJ}\right]} \end{array}$$

Third, energy service expenditure rates ( ${\overset{\cdot }{C}}_{s}$ ) are the product of energy price ( $p_{E}$ ) and device energy consumption rates ( ${\overset{\cdot }{E}}_{s}$ ).

(8) $${\overset{\cdot }{C}}_{s}=p_{E}{\overset{\cdot }{E}}_{s}$$

[$/yr]=[$/MJ][MJ/yr]

Fourth, indirect energy rates for operations and maintenance ( ${\overset{\cdot }{E}}_{\mathrm{OM}}$ ), disposal ( ${\overset{\cdot }{E}}_{d}$ ), and other goods expenditures ( ${\overset{\cdot }{E}}_{g}$ ) are the product of expenditures rates ( ${\overset{\cdot }{C}}_{\mathrm{OM}}$ , $\tau_{\omega }{\overset{\cdot }{C}}_{d}$ , and ${\overset{\cdot }{C}}_{g}$ ) and the energy intensity of the economy ( $I_{E}$ ).

(9) $${\overset{\cdot }{E}}_{\mathrm{OM}}={\overset{\cdot }{C}}_{\mathrm{OM}}I_{E}$$

(10) $${\overset{\cdot }{E}}_{d}=\tau_{\omega }{\overset{\cdot }{C}}_{d}I_{E}$$

(11) $${\overset{\cdot }{E}}_{g}={\overset{\cdot }{C}}_{g}I_{E}$$

[MJ/yr]=[$/yr][MJ/$]

Note that the indirect energy rate for the disposal effect is obtained from disposal costs that include discounting. (See Appendix B.1 for details on cost discounting.)

### 2.5. Rebound Effects

The four rebound effects (emplacement, substitution, income, and macro) are discussed in subsections below. In each subsection, we define the effect and show mathematical expressions for rebound ( Re ) caused by the effect. Detailed derivations of all rebound expressions can be found in Appendix B. See, in particular, Tables B.3 to B.6, which provide a parallel structure for energy and financial accounting across all rebound effects. We begin with the emplacement effect.

#### 2.5.1. Emplacement Effect

The emplacement effect accounts for performance changes of the device due to the fact that a higher-efficiency device has been put in service (and will need to be decommissioned at a later date); consumption patterns are assumed unchanged. Behavior adjustments are addressed later, in the substitution and income effects. Any (positive or negative) adjustment in income due to emplacement (measured as net income, ${\overset{\cdot }{N}}^{*}$ ) is added to the freed cash ( $\overset{\cdot }{G}$ ) spent in the income effect.

*Direct emplacement effect* ( $Re_{\mathrm{dempl}}$ ). The direct emplacement effects of the EEU include device energy savings ( ${\overset{\cdot }{S}}_{\mathrm{dev}}$ ) and device energy cost savings ( $\Delta {\overset{\cdot }{C}}_{s}^{*}$ ). ${\overset{\cdot }{S}}_{\mathrm{dev}}$ can be written conveniently as

(12) $${\overset{\cdot }{S}}_{\mathrm{dev}}=\left(\frac{\eta^{*}}{\eta^{{}^{\circ}}}-1\right)\;\frac{\eta^{{}^{\circ}}}{\eta^{*}}{\overset{\cdot }{E}}_{s}^{{}^{\circ}}.$$

(See Appendix B.4.1 for the derivation.)

Because the original and upgraded device are assumed to have equal performance^12^ and because behavior changes are not considered in the direct emplacement effect, actual and expected energy savings rates are identical, and there is no takeback. By definition, then, the direct emplacement effect causes no rebound. Thus,

(13) $$Re_{\mathrm{dempl}}=0.$$

*Indirect emplacement effects* ( $Re_{\mathrm{iempl}}$ ). Although the direct emplacement effect does not cause rebound, indirect emplacement effects may indeed cause rebound. Indirect emplacement effects account for the life cycle of the energy conversion device, including (i) changes in the embodied energy rate ( $\Delta {\overset{\cdot }{E}}_{\mathrm{emb}}^{*}$ ), (ii) changes in the operations and maintenance energy and expenditure rates ( $\Delta {\overset{\cdot }{E}}_{\mathrm{OM}}^{*}$ and $\Delta {\overset{\cdot }{C}}_{\mathrm{OM}}^{*}$ ), and (iii) changes in the disposal energy and expenditure rates ( $\Delta {\overset{\cdot }{E}}_{d}^{*}$ and $\Delta {\overset{\cdot }{C}}_{d}^{*}$ ).

*Embodied energy effect* ( $Re_{\mathrm{emb}}$ ). One of the unique features of this framework is that independent analyses of embodied energy and capital costs of the EEU are required. We note that the different terms (embodied energy rate, ${\overset{\cdot }{E}}_{\mathrm{emb}}$ , and capital cost rate, ${\overset{\cdot }{C}}_{\mathrm{cap}}$ ) might seem to imply different processes, but they actually refer to the same emplacement effect. Purchasing an upgraded device (which likely leads to ${\overset{\cdot }{C}}_{\mathrm{cap}}^{{}^{\circ}}\neq {\overset{\cdot }{C}}_{\mathrm{cap}}^{*}$ ) will likely mean a changed embodied energy rate ( ${\overset{\cdot }{E}}_{\mathrm{emb}}^{{}^{\circ}}\neq {\overset{\cdot }{E}}_{\mathrm{emb}}^{*}$ ) to provide the same energy service. Our names for these aspects of rebound (embodied energy and capital cost) reflect common usage in the energy and economics fields, respectively.

Consistent with the energy analysis literature, we define embodied energy to be the sum of all energy consumed in the production of the energy conversion device, all the way back to resource extraction.^13^ Energy is embodied in the device within manufacturing and distribution supply chains prior to consumer acquisition of the device. We assume no energy is embodied in the device while in service. The EEU causes the embodied energy of the energy conversion device to change from $E_{\mathrm{emb}}^{{}^{\circ}}$ to $E_{\mathrm{emb}}^{*}$ .

For simplicity, we spread all embodied energy evenly over the lifetime of the device which gives a constant embodied energy rate ( ${\overset{\cdot }{E}}_{\mathrm{emb}}$ ). Thus, we allocate embodied energy over the life of the original and upgraded devices ( $t_{\mathrm{life}}^{{}^{\circ}}$ and $t_{\mathrm{life}}^{*}$ , respectively) without discounting to obtain embodied energy rates, such that ${\overset{\cdot }{E}}_{\mathrm{emb}}^{{}^{\circ}}=E_{\mathrm{emb}}^{{}^{\circ}}/t_{\mathrm{life}}^{{}^{\circ}}$ and ${\overset{\cdot }{E}}_{\mathrm{emb}}^{*}=E_{\mathrm{emb}}^{*}/t_{\mathrm{life}}^{*}$ . The change in embodied final energy due to the EEU (expressed as a rate) is given by $\Delta {\overset{\cdot }{E}}_{\mathrm{emb}}^{*}={\overset{\cdot }{E}}_{\mathrm{emb}}^{*}-{\overset{\cdot }{E}}_{\mathrm{emb}}^{{}^{\circ}}$ . The expression for embodied energy rebound is

(14) $$Re_{\mathrm{emb}}=\frac{\left(\frac{E_{\mathrm{emb}}^{*}}{E_{\mathrm{emb}}^{{}^{\circ}}}\frac{t_{\mathrm{life}}^{{}^{\circ}}}{t_{\mathrm{life}}^{*}}-1\right){\overset{\cdot }{E}}_{\mathrm{emb}}^{{}^{\circ}}}{{\overset{\cdot }{S}}_{\mathrm{dev}}}.$$

(See Appendix B.4.2 for details of the derivation.)

Embodied energy rebound ( $Re_{\mathrm{emb}}$ ) can be either positive or negative, depending on the sign of the term $\left(E_{\mathrm{emb}}^{*}/E_{\mathrm{emb}}^{{}^{\circ}}\right)\left(t_{\mathrm{life}}^{{}^{\circ}}/t_{\mathrm{life}}^{*}\right)-1$ . Rising energy efficiency can be associated with increased device complexity, additional energy consumption in manufacturing, and more embodied energy, such that $E_{\mathrm{emb}}^{{}^{\circ}}\;<\;E_{\mathrm{emb}}^{*}$ and $Re_{\mathrm{emb}}\;>\;0$ , all other things being equal. However, if the upgraded device has longer life than the original device ( $t_{\mathrm{life}}^{*}\;>\;t_{\mathrm{life}}^{{}^{\circ}}$ ), ${\overset{\cdot }{E}}_{\mathrm{emb}}^{*}-{\overset{\cdot }{E}}_{\mathrm{emb}}^{{}^{\circ}}$ could be negative, meaning that the upgraded device has a lower embodied energy rate than the original device.

*Operations, maintenance, and disposal effects* ( $Re_{\mathrm{OMd}}$ ). In addition to embodied energy, indirect emplacement effect rebound accounts for energy demanded by operations and maintenance (subscript OM ) and disposal (subscript d ) activities. Operations and maintenance expenditures are typically modeled as a per-year expense, a rate (e.g., ${\overset{\cdot }{C}}_{\mathrm{OM}}^{*}$ ). On the other hand, disposal costs (e.g., $C_{d}^{{}^{\circ}}$ ) are incurred at the end of the useful life of the energy conversion device (subscript ω ). We annualize disposal costs (with discounting) across the lifetime of the original and upgraded devices ( $t_{\mathrm{life}}^{{}^{\circ}}$ and $t_{\mathrm{life}}^{*}$ , respectively) to form discounted expenditure rates such that ${\overset{\cdot }{C}}_{\mathrm{OMd}}^{{}^{\circ}}={\overset{\cdot }{C}}_{\mathrm{OM}}^{{}^{\circ}}+\tau_{\omega }^{{}^{\circ}}{\overset{\cdot }{C}}_{d}^{{}^{\circ}}$ and ${\overset{\cdot }{C}}_{\mathrm{OMd}}^{*}={\overset{\cdot }{C}}_{\mathrm{OM}}^{*}+\tau_{\omega }^{*}{\overset{\cdot }{C}}_{d}^{*}$ .

For simplicity, we assume that operations, maintenance, and disposal expenditures imply energy consumption elsewhere in the economy at its overall energy intensity ( $I_{E}$ ). Therefore, the change in energy consumption rate caused by a change in maintenance and disposal expenditures is given by $\Delta {\overset{\cdot }{C}}_{\mathrm{OMd}}^{*}I_{E}=\left({\overset{\cdot }{C}}_{\mathrm{OMd}}^{*}-{\overset{\cdot }{C}}_{\mathrm{OMd}}^{{}^{\circ}}\right)I_{E}$ . Rebound from operations, maintenance, and disposal activities is given by

(15) $$Re_{\mathrm{OMd}}=\frac{\left(\frac{{\overset{\cdot }{C}}_{\mathrm{OMd}}^{*}}{{\overset{\cdot }{C}}_{\mathrm{OMd}}^{{}^{\circ}}}-1\right){\overset{\cdot }{C}}_{\mathrm{OMd}}^{{}^{\circ}}I_{E}}{{\overset{\cdot }{S}}_{\mathrm{dev}}}.$$

(See Appendix B.4.2 for details of the derivation.)

#### 2.5.2. Substitution Effect

Neoclassical economic theory determines consumer behavior through utility maximization. It decomposes price-induced behavior change into (i) substituting energy service consumption for other goods consumption due to the lower post-EEU price of the energy service (the substitution effect) and (ii) spending of the higher real income (the income effect).^14^ This section develops mathematical expressions for substitution effect rebound ( $Re_{\mathrm{sub}}$ ), thereby accepting the standard neoclassical microeconomic assumptions about consumer behavior.^15^ (The next section addresses income effect rebound, $Re_{\mathrm{inc}}$ .) The substitution effect determines compensated demand, which is the demand for the expenditure-minimizing consumption bundle that maintains utility at the pre-EEU level, given the new prices. Compensated demand is a technical term for a thought experiment from welfare economics: the device user’s budget is altered so that the user is “compensated” for the change in price so as to maintain the same level of utility as before. In the case of an EEU, this implies the budget is reduced because the energy service price has fallen, so that it becomes cheaper to maintain a given level of utility. The change in the budget is called “compensating variation” (CV). The substitution effect involves (i) an increase in consumption of the energy service, the direct substitution effect (subscript dsub ) and (ii) a decrease in consumption of other goods, the indirect substitution effect (subscript isub ). Thus, two terms comprise substitution effect rebound: direct substitution rebound ( $Re_{\mathrm{dsub}}$ ) and indirect substitution rebound ( $Re_{\mathrm{isub}}$ ).

After emplacement of the more efficient device (but before the substitution effect), the price of the energy service decreases ( $p_{s}^{{}^{\circ}}\;>\;p_{s}^{*}$ ). After compensating variation tightens the budget constraint, consumption at the new energy service price ( $p_{s}^{*}$ ) yields utility at the same level as prior to the EEU by consuming more of the now-lower-cost energy service (subscript s ) and less of the now-relatively-more-expensive other goods (subscript g ).

A constant price elasticity (CPE) utility model is often used in the literature (e.g., see Borenstein 2015, 17: footnote 43) for determining post-substitution effect consumption and therefore $Re_{\mathrm{dsub}}$ and $Re_{\mathrm{isub}}$ (see Appendix B.4.3). However, the CPE utility model can deliver only an approximation of the substitution effect for two reasons. First, because it is a reduced form model and only uncompensated elasticities are observed, the CPE utility model reports the sum of direct substitution effect and direct income effect rebound ( $Re_{\mathrm{dsub}}+Re_{\mathrm{dinc}}$ ). Second, price elasticities typically change as consumption bundles change, whereas the CPE price elasticity remains constant by definition. Typically, constant price elasticities (as in the CPE utility model) are approximations that are applicable only to marginal price changes. As shown in Heun et al. (2025), these approximations can lead to small or large errors depending on the case, relative to the exact model, which we introduce next. Appendix C derives changes in price elasticities for non-CPE models.

Here, we present a constant elasticity of substitution (CES) utility model that allows all of the uncompensated own price elasticity ( $\varepsilon_{{\overset{\cdot }{q}}_{s},p_{s}}$ ), the uncompensated cross price elasticity ( $\varepsilon_{{\overset{\cdot }{q}}_{g},p_{s}}$ ), the compensated own price elasticity ( $\varepsilon_{{\overset{\cdot }{q}}_{s},p_{s},c}$ ), and the compensated cross price elasticity ( $\varepsilon_{{\overset{\cdot }{q}}_{g},p_{s},c}$ ) to vary along an indifference curve, thereby enabling numerically precise analysis of non-marginal energy service price changes ( $p_{s}^{{}^{\circ}}>>p_{s}^{*}$ ). The CES utility model allows the direct calculation of the utility-maximizing consumption bundle for any constraint, describing the device user’s behavior as

(16) $$\frac{\overset{\cdot }{u}}{{\overset{\cdot }{u}}^{{}^{\circ}}}={\left[f_{{\overset{\cdot }{C}}_{s}}^{{}^{\circ}}{\left(\frac{{\overset{\cdot }{q}}_{s}}{{\overset{\cdot }{q}}_{s}^{{}^{\circ}}}\right)}^{\rho }+\left(1-f_{{\overset{\cdot }{C}}_{s}}^{{}^{\circ}}\right){\left(\frac{{\overset{\cdot }{C}}_{g}}{{\overset{\cdot }{C}}_{g}^{{}^{\circ}}}\right)}^{\rho }\right]}^{\left(1/\rho \right)}.$$

The device user’s utility rate (relative to the original condition, ${\overset{\cdot }{u}}^{{}^{\circ}}$ ) is determined by the consumption rate of the energy service ( ${\overset{\cdot }{q}}_{s}$ ) and the consumption rate of other goods and services ( ${\overset{\cdot }{C}}_{g}$ ). The share parameter ( $f_{{\overset{\cdot }{C}}_{s}}^{{}^{\circ}}$ ) between ${\overset{\cdot }{q}}_{s}$ and ${\overset{\cdot }{C}}_{g}$ is taken from the original (pre-EEU) consumption basket. The exponent ρ is calculated from the (constant) elasticity of substitution ( σ ) as ρ≡(σ−1)/σ . All quantities are normalized to pre-EEU values so that the cost share of other goods can be used straightforwardly in empirical applications rather than having to construct quantity and price indices. The normalized specification is commonly used in empirical CES *production* function applications (Gechert et al. 2021; Klump, Mcadam and Willman 2012; Temple 2012). See Appendix C for further details of the CES utility model.

Direct substitution effect rebound ( $Re_{\mathrm{dsub}}$ ) is

(17) $$Re_{\mathrm{dsub}}=\frac{\Delta {\hat{\overset{\cdot }{E}}}_{s}}{{\overset{\cdot }{S}}_{\mathrm{dev}}}\;,$$

which can be rearranged to

(18) $$Re_{\mathrm{dsub}}=\frac{\frac{{\hat{\overset{\cdot }{q}}}_{s}}{{\overset{\cdot }{q}}_{s}^{{}^{\circ}}}-1}{\frac{\hat{\eta }}{\eta^{{}^{\circ}}}-1}.$$

Indirect substitution effect rebound ( $Re_{\mathrm{isub}}$ ) is given by

(19) $$Re_{\mathrm{isub}}=\frac{\Delta {\hat{\overset{\cdot }{C}}}_{g}I_{E}}{{\overset{\cdot }{S}}_{\mathrm{dev}}}\;,$$

which can be rearranged to

(20) $$Re_{\mathrm{isub}}=\frac{\frac{{\hat{\overset{\cdot }{C}}}_{g}}{{\overset{\cdot }{C}}_{g}^{{}^{\circ}}}-1}{\frac{\hat{\eta }}{\eta^{{}^{\circ}}}-1}\;\frac{\hat{\eta }}{\eta^{{}^{\circ}}}\;\frac{{\overset{\cdot }{C}}_{g}^{{}^{\circ}}I_{E}}{{\overset{\cdot }{E}}_{s}^{{}^{\circ}}}.$$

To find the post-substitution effect point (∧), we solve for the location on the indifference curve where its slope is equal to the slope of the post-EEU expenditure line, assuming the CES utility model.^16^ The results are

(21) $$\frac{{\hat{\overset{\cdot }{q}}}_{s}}{{\overset{\cdot }{q}}_{s}^{{}^{\circ}}}={\left\{f_{{\overset{\cdot }{C}}_{s}}^{{}^{\circ}}+\left(1-f_{{\overset{\cdot }{C}}_{s}}^{{}^{\circ}}\right){\left[\left(\frac{1-f_{{\overset{\cdot }{C}}_{s}}^{{}^{\circ}}}{f_{{\overset{\cdot }{C}}_{s}}^{{}^{\circ}}}\right)\frac{p_{s}^{*}{\overset{\cdot }{q}}_{s}^{{}^{\circ}}}{{\overset{\cdot }{C}}_{g}^{{}^{\circ}}}\right]}^{\rho /\left(1-\rho \right)}\right\}}^{-1/\rho }$$

and

(22) $$\frac{{\hat{\overset{\cdot }{C}}}_{g}}{{\overset{\cdot }{C}}_{g}^{{}^{\circ}}}={\left(1+f_{{\overset{\cdot }{C}}_{s}}^{{}^{\circ}}\left\{{\left[\left(\frac{1-f_{{\overset{\cdot }{C}}_{s}}^{{}^{\circ}}}{f_{{\overset{\cdot }{C}}_{s}}^{{}^{\circ}}}\right)\frac{p_{s}^{*}{\overset{\cdot }{q}}_{s}^{{}^{\circ}}}{{\overset{\cdot }{C}}_{g}^{{}^{\circ}}}\right]}^{\rho /\left(\rho -1\right)}-1\right\}\right)}^{-1/\rho }.$$

Equation (21) can be substituted directly into equation (18) to obtain an expression for direct substitution rebound ( $Re_{\mathrm{dsub}}$ ) via the CES utility model.

(23) $$Re_{\mathrm{dsub}}=\frac{{\left\{f_{{\overset{\cdot }{C}}_{s}}^{{}^{\circ}}+\left(1-f_{{\overset{\cdot }{C}}_{s}}^{{}^{\circ}}\right){\left[\left(\frac{1-f_{{\overset{\cdot }{C}}_{s}}^{{}^{\circ}}}{f_{{\overset{\cdot }{C}}_{s}}^{{}^{\circ}}}\right)\frac{p_{s}^{*}{\overset{\cdot }{q}}_{s}^{{}^{\circ}}}{{\overset{\cdot }{C}}_{g}^{{}^{\circ}}}\right]}^{\rho /\left(1-\rho \right)}\right\}}^{-1/\rho }-1}{\frac{\hat{\eta }}{\eta^{{}^{\circ}}}-1}$$

Equation (22) can be substituted directly into equation (20) to obtain an expression for indirect substitution rebound ( $Re_{\mathrm{isub}}$ ) via the CES utility model.

(24) $$Re_{\mathrm{isub}}=\frac{{\left(1+f_{{\overset{\cdot }{C}}_{s}}^{{}^{\circ}}\left\{{\left[\left(\frac{1-f_{{\overset{\cdot }{C}}_{s}}^{{}^{\circ}}}{f_{{\overset{\cdot }{C}}_{s}}^{{}^{\circ}}}\right)\frac{p_{s}^{*}{\overset{\cdot }{q}}_{s}^{{}^{\circ}}}{{\overset{\cdot }{C}}_{g}^{{}^{\circ}}}\right]}^{\rho /\left(\rho -1\right)}-1\right\}\right)}^{-1/\rho }-1}{\frac{\hat{\eta }}{\eta^{{}^{\circ}}}-1}\frac{\hat{\eta }}{\eta^{{}^{\circ}}}\;\frac{{\overset{\cdot }{C}}_{g}^{{}^{\circ}}I_{E}}{{\overset{\cdot }{E}}_{s}^{{}^{\circ}}}$$

(See Appendix B.4.3 for details of the derivations of equations (18), (20), and (21)–(24).)

#### 2.5.3. Income Effect

The monetary income rate of the device user ( $\overset{\cdot }{M}$ ) remains unchanged across the rebound effects. Thanks to the energy service price decline, real income rises, and freed cash from the EEU is given as $\overset{\cdot }{G}=p_{E}{\overset{\cdot }{S}}_{\mathrm{dev}}$ . (See equation (90) in Appendix B.3.) Emplacement effect adjustments and compensating variation modify freed cash to leave the device user with *net* savings ( $\hat{\overset{\cdot }{N}}$ ) from the EEU, as shown in equation (100) in Appendix B.3. (Derivations of expressions for freed cash from the emplacement effect ( $\overset{\cdot }{G}$ ) and net savings after the substitution effect ( $\hat{\overset{\cdot }{N}}$ ) are presented in Tables B.3 and B.4.) Rebound from the income effect quantifies the rate of additional energy demand that arises when the energy conversion device user spends net savings from the EEU.

Additional energy demand from the income effect is determined by several constraints. The income effect under utility maximization satisfies the budget constraint, so that net savings are zero after the income effect ( $\bar{\overset{\cdot }{N}}=0$ ). (See Appendix D for a mathematical proof that the income preference equations below (equations (25) and (29)) satisfy the budget constraint.)

A second constraint is that net savings are spent completely on (i) additional consumption of the energy service ( ${\hat{\overset{\cdot }{q}}}_{s}\;<\;{\bar{\overset{\cdot }{q}}}_{s}$ ) and (ii) additional consumption of other goods ( ${\hat{\overset{\cdot }{q}}}_{g}\;<\;{\bar{\overset{\cdot }{q}}}_{g}$ ). The proportions in which income-effect spending is allocated depends on the utility model, which prescribes the income expansion path for consumption. Given post-EEU prices, maximized CES utility means spending in the same proportion on the energy service and other goods across the income effect, a property known as homotheticity. This constraint is satisfied by construction below, particularly via an effective income term ( M·^' ).

However, this framework could accommodate non-homothetic preferences for spending across the income effect (turning the income expansion path into a more general curve instead of a line). Demand for certain energy services could satiate as consumers become more affluent, implying income elasticities of the energy service of less than one (Greening, Greene and Difiglio 2000). At the lower bound, the consumer spends all income after the substitution effect on other goods (subscript g ) and none on the energy service (subscript s ), choices that serve to reduce rebound due to typically lower energy intensity of other goods compared to the energy service.^17^

We next show expressions for direct and indirect income effect rebound.

*Direct income effect* ( $Re_{\mathrm{dinc}}$ ). The income elasticity of energy service demand ( $\varepsilon_{{\overset{\cdot }{q}}_{s},\overset{\cdot }{M}}$ ) quantifies the amount of net savings spent on more of the energy service ( ${\hat{\overset{\cdot }{q}}}_{s}\;<\;{\bar{\overset{\cdot }{q}}}_{s}$ ). (See Appendix C for additional information about elasticities.) Spending of net savings on additional energy service consumption leads to direct income effect rebound ( $Re_{\mathrm{dinc}}$ ).

The ratio of rates of energy service consumed across the income effect is given by

(25) q·¯sq·^s=(1+N·^M·^')εq·s,M·.

Under the CES utility model, homotheticity means that $\varepsilon_{{\overset{\cdot }{q}}_{s},\overset{\cdot }{M}}=1$ .

Effective income ( M·^' ) is given by

(26) M·^'≡M·−τα*C·cap*−C·OMd*−N·^.

For the purposes of the income effect, effective income (equation (26)) adjusts original income ( ${\overset{\cdot }{M}}^{{}^{\circ}}$ ) to account for sunk costs ( $\tau_{\alpha }^{*}{\overset{\cdot }{C}}_{\mathrm{cap}}^{*}$ and ${\overset{\cdot }{C}}_{\mathrm{OMd}}^{*}$ ) and net savings ( $\hat{\overset{\cdot }{N}}$ ).

Direct income rebound is defined as

(27) $$Re_{\mathrm{dinc}}\;\equiv \;\frac{\Delta {\bar{\overset{\cdot }{E}}}_{s}}{{\overset{\cdot }{S}}_{\mathrm{dev}}}.$$

(See Table B.5.) After substitution, rearranging, and canceling of terms (Appendix B.4.4), the expression for direct income rebound under the CES utility model is

(28) Redinc=(1+N·^M·^')εq·s,M·−1η*η°−1{fC·s°+(1−fC·s°)[(1−fC·s°fC·s°)ps*q·s°C·g°]ρ/(1−ρ)}−1/ρ.

If there are no net savings after the substitution effect ( $\hat{\overset{\cdot }{N}}=0$ ), direct income effect rebound is zero ( $Re_{\mathrm{dinc}}=0$ ), as expected.^18^

Under a non-homothetic utility model, the bounding condition is satiated consumption of the energy service such that as the device owner becomes richer, none of the net income ( $\hat{\overset{\cdot }{N}}$ ) is spent on more of the energy service, and thus $Re_{\mathrm{dinc}}=0$ would occur.

*Indirect income effect* ( $Re_{\mathrm{iinc}}$ ). Not all net savings ( $\hat{\overset{\cdot }{N}}$ ) are spent on more energy for the energy conversion device. The income elasticity of other goods demand ( $\varepsilon_{{\overset{\cdot }{q}}_{g},\overset{\cdot }{M}}$ ) quantifies the amount of net savings spent on additional other goods ( ${\hat{\overset{\cdot }{q}}}_{s}\;<\;{\bar{\overset{\cdot }{q}}}_{s}$ ). Spending of net savings on additional other goods and services leads to indirect income effect rebound ( $Re_{\mathrm{iinc}}$ ).

The ratio of rates of other goods consumed across the income effect is given by

(29) q·¯gq·^g=(1+N·^M·^')εq·g,M·.

Under the assumption that prices of other goods are exogenous (see Appendix E), the ratio of rates of other goods consumption ( ${\bar{\overset{\cdot }{q}}}_{g}/{\hat{\overset{\cdot }{q}}}_{g}$ ) is equal to the ratio of rates of other goods expenditures ( ${\bar{\overset{\cdot }{C}}}_{g}/{\hat{\overset{\cdot }{C}}}_{g}$ ) such that

(30) C·¯gC·^g=(1+N·^M·^')εq·g,M·.

Homotheticity means that $\varepsilon_{{\overset{\cdot }{q}}_{g},\overset{\cdot }{M}}=1$ . As shown in Table B.5, indirect income rebound is defined as

(31) $$Re_{\mathrm{iinc}}\;\equiv \;\frac{\Delta {\bar{\overset{\cdot }{C}}}_{g}I_{E}}{{\overset{\cdot }{S}}_{\mathrm{dev}}}.$$

After substitution, rearranging, and canceling of terms, the expression for indirect income rebound under the CES utility model is

(32) Reiinc=(1+N·^M·^')εq·g,M·−1η*η°−1(η*η°)C·g°IEE·s°×(1+fC·s°{[(1−fC·s°fC·s°)ps*q·s°C·g°]ρ/(ρ−1)−1})−1/ρ.

(See Appendix B.4.4 for details of the derivation of direct and indirect income effect rebound.)

Under the bounding satiated utility model, all net income ( $\hat{\overset{\cdot }{N}}$ ) is spent on other goods, and indirect rebound becomes simply $Re_{\mathrm{iinc}}=\frac{\hat{\overset{\cdot }{N}}I_{E}}{{\overset{\cdot }{S}}_{\mathrm{dev}}}$ .

#### 2.5.4. Macro Effect

The previous rebound effects (emplacement effect, substitution effect, and income effect) occur at the microeconomic level. However, changes at the microeconomic level can have important impacts at the macroeconomic or economy-wide level.

It is one of the basic tenets of economics that productivity gains have been the main long-run driver of economic growth in the last couple of centuries (Marx 1867; Smith 1776; Solow 1957). Interest in the impact of individual sectors on the whole economy reaches arguably even farther back (Quesnay 1759) and continues to the present (Leontief 1986). Recent work revived interest in firm- and sector-specific shocks on aggregate output and demonstrates that due to interlinkages between firms and sectors, productivity shocks in a firm or sector can have larger macroeconomic consequences than the original shock (Acemoglu et al. 2012; Baqaee and Farhi 2019; Gabaix 2011). Foerster et al. (2022) estimate that 3/4 of long-run U.S. growth since 1950 can be attributed to sector-specific (as opposed to aggregate) trend factors. Because the EEU represents a positive, sector-specific productivity shock, the same principles apply. These kinds of rebounds can be captured by a general equilibrium model (Stern 2020), but we propose a simple rule for incorporating this macroeconomic effect of productivity growth into our partial equilibrium framework.

Before establishing a formalism for $Re_{\mathrm{macro}}$ , we clarify the link between consumer theory and economic growth. Turner (2013) cautions that when households see the productivity of their non-market activities increase, GDP remains unchanged.^19^ That may be true in the short run. But the question over longer periods is whether the more productive household energy services do not also feed through into economic growth accounted for by GDP. People in affluent countries spend about as much time on unpaid (i.e., non-market) work as on paid work (Folbre 2021). Therefore productivity improvements in unpaid work can spill over into paid work, which enters GDP. One channel could be time-saving. If the EEU saves time, then saved time could be spent on more paid work or on increasing human capital (Gautham and Folbre 2024; Sorrell and Dimitropoulos 2008). If the EEU saves money (but no time), then the freed cash could be spent to create additional demand for products that translate into higher GDP and possibly faster productivity growth (Magacho and McCombie 2018). The freed cash could also be spent on more effective (and more costly) human capital-increasing activities or even be used to start a venture. In all cases, it would be rash to conclude that just because some EEUs lead to productivity increases not captured directly by GDP, they do not eventually lead to additional economic growth.^20^

Borenstein also addressed these macro effects from consumer behavior noting that “income effect rebound will be larger economy-wide than would be inferred from evaluating only the direct income gain from the end user’s transaction” (Borenstein 2015, 11) and likened it to a macroeconomic multiplier.^21^ The sectoral growth shock literature also uses multipliers to conceptualize the impacts of sectoral productivity shocks on aggregate output (Buera and Trachter 2024; Foerster et al. 2022). Using multipliers has the advantage that they can be directly linked to the income effect (minus compensating variation) and its consequence for macroeconomic rebound. Borenstein also notes that scaling from net savings ( ${\overset{\cdot }{N}}^{*}$ ) at the device level to productivity-driven growth at the macro level is unexplored territory.

We operationalize the macro rebound multiplier idea by noting that higher productivity makes the device cheaper to operate (and possibly purchase), which allows consumers to purchase a larger bundle of goods and services. If the overall expansion of the economy is a multiple of the direct increase in productivity expressed as productivity gains in other sectors, then the macro effect can simply be represented as a multiple of the (indirect) emplacement effect at the post-emplacement stage (*) of Figure 1, a multiplier that we represent by a macro factor ( k ).^22^

The macro factor ( k ) represents respending in the broader economy after the emplacement effect has occurred and is not tied to any particular EEU or economic sector. k≥0 is expected. k=0 means there is no macroeconomic effect resulting from the energy efficiency upgrade. k>0 means that productivity-driven macroeconomic growth has occurred with consequent implications for additional energy consumption in the wider economy.

We assume as a first approximation (following Antal and van den Bergh 2014; Borenstein 2015) that macro effect responding implies energy consumption according to the average energy intensity of the economy ( $I_{E}$ ). Macro rebound is therefore given by

(33) $$Re_{\mathrm{macro}}=\frac{k{\overset{\cdot }{N}}^{*}I_{E}}{{\overset{\cdot }{S}}_{\mathrm{dev}}}.$$

(See Table B.6.) After some algebra (Appendix B.4.5), we arrive at an expression for macro effect rebound:

(34) $$Re_{\mathrm{macro}}=k\left(p_{E}I_{E}-Re_{\mathrm{cap}}-Re_{\mathrm{OMd}}\right).$$

Another macroeconomic rebound could arise from the energy price, which could fall due to lower demand (Borenstein 2015; Gillingham, Rapson and Wagner 2016). The size of the energy price effect depends on the size of the energy savings from the EEU relative to the energy demand in the economy. Therefore, calculating the energy price effect requires additional assumptions about how many households adopt the new device, which we consider to be outside the scope of our core framework. However, we show how it could be incorporated by adding an assumption about EEU adoption shares and a model of the energy market to derive a rebound expression for the energy price effect in Section 3.2 and Appendix F.

### 2.6. Rebound Sum

The sum of all rebound emerges from the four rebound effects (emplacement effect, substitution effect, income effect, and macro effect). Macro effect rebound ( $Re_{\mathrm{macro}}$ in equation (34)) is expressed in terms of other rebound effects. (Derivation details can be found in Appendix B.4.6.) After algebra and canceling of terms, we find

(35) $$\begin{array}{c} Re_{\mathrm{tot}}=Re_{\mathrm{emb}}+k\left(p_{E}I_{E}-Re_{\mathrm{cap}}\right)+\left(1-k\right)Re_{\mathrm{OMd}}+\\ \;\;\;\;\;Re_{\mathrm{dsub}}+Re_{\mathrm{isub}}+Re_{\mathrm{dinc}}+Re_{\mathrm{iinc}}. \end{array}$$

## 3. Discussion

### 3.1. Comparison to Other Rebound Frameworks

We developed above a rebound framework for consumers. We note that many of its components are similar to those for a producer-sided framework due to symmetries between neoclassical microeconomic producer and consumer theory. Ours is a partial equilibrium framework at the microeconomic level that provides a detailed assessment of individual EEUs with tractable, easy-to-understand mathematics. Partial equilibrium frameworks are easier to understand, in part, because they constrain price variation to the energy service only; all other prices remain constant (at least at the microeconomic level).^23^ In our framework, general equilibrium effects and other dynamic effects at the macroeconomic level are captured by a simplified, one-dimensional rebound effect discussed in Section 2.5.4.

We are not the first to develop a rebound analysis framework, so it is worthwhile to compare our framework to others for key features: analysis of all rebound effects; analysis of energy, expenditure, and consumption aspects of rebound; level of detail in the consumer preference model; allowance for non-marginal energy efficiency changes; and empirical application. When all of the above characteristics are present, a fuller picture of rebound can emerge.^24^ Table 2 shows our assessment of selected previous partial equilibrium frameworks (in columns) relative to the characteristics discussed above (in rows).

**Table 2.** Comparison Among Relevant Rebound Analysis Frameworks.

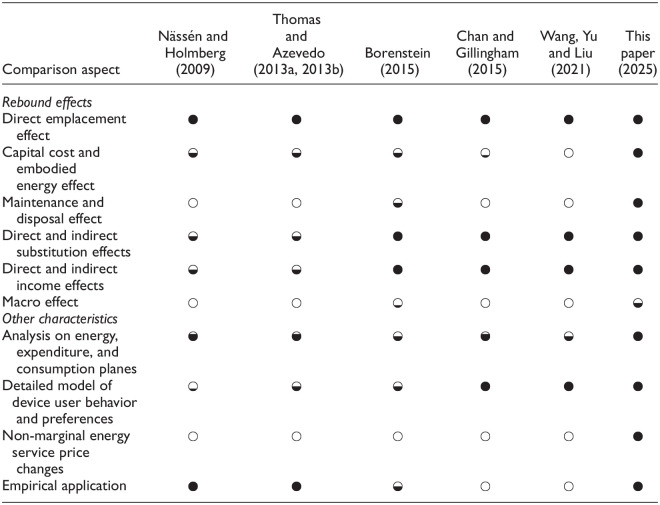

*Note*. Empty (white) circles indicate no treatment of a subject by a framework. Partly and fully filled circles indicate partial and comprehensive treatment of a subject by a framework.

Because all frameworks evaluate the expected decrease in direct energy consumption from the EEU, the “Direct emplacement effect” row contains • in all columns. Three early papers (Nässén and Holmberg 2009; Thomas and Azevedo 2013a, 2013b) estimate rebound quantitatively, earning high marks (•) in the “Empirical application” row. Both Nässén and Holmberg and Thomas and Azevedo motivate their frameworks at least partially with microeconomic theory (consumer preferences and substitution and income effects) but use simple linear demand functions in their empirical analyses. Thus, the connection between economic theory and empirics is tenuous, leading to intermediate ratings (◒ or less) in the “substitution effects,”“income effects,” and “Detailed model of consumer preferences” rows. More recently, Chan and Gillingham (2015) and Wang, Yu and Liu (2021) anchor the rebound effect firmly in consumer theory, earning high ratings (•) in the “substitution effects,”“income effects,” and “Detailed model of consumer preferences” rows. They extend their frameworks to advanced topics that our framework does not presently incorporate, such as multiple fuels, energy services, and nested utility functions with intermediate inputs. However, neither Chan and Gillingham nor Wang et al. provide empirical applications, earning ○ in the last row of Table 2. In the middle of the table (and between the other studies in time), the framework by Borenstein (2015) touches on nearly all important characteristics. However, the Borenstein framework cannot separate substitution and income effects cleanly in empirical analysis, reverting to partial analyses of both, leading to a ◒ rating in the “Detailed model of consumer preferences” and “Empirical application” rows.

No previous framework engages fully with either the differential financial effects or the differential energetic effects of the upfront purchase of the upgraded device, leading to low ratings across all previous frameworks in the “Capital cost and embodied energy effect” row. In fact, except for Nässén and Holmberg (2009), no framework engages with capital costs, although all note its importance. (Nässén and Holmberg note that capital costs and embodied energy can have very strong effects on rebound.) Thomas and Azevedo (2013a, 2013b) provide the only framework that traces embodied energy effects of every consumer good using input-output methods, but they do not analyze embodied energy of the upgraded device. Borenstein (2015) notes the embodied energy of the upgraded device and the embodied energy of other goods but does not integrate embodied energy or financing costs into the framework for empirical analysis. Borenstein is, however, the only author to treat the financial side of embodied energy or maintenance and disposal effects. Borenstein (2015) postulates the macro effect, but does not operationalize the link between micro and macro levels, earning ◒ in the “Macro effect” row. No other framework even discusses the link between macro and micro rebound effects, leading to ° in the “Macro effect” row for all previous frameworks (apart from Borenstein 2015). Our framework operationalizes the link between micro and macro levels, via the macro factor ( k ), but more work can be done in this area. Thus, “This paper (2025)” earns ◒ in the “Macro effect” row. Finally, all previous frameworks assume constant price elasticities and implicitly marginal or small improvements in efficiency, excluding the numerically precise analysis of important non-incremental upgrades where price elasticities are likely to vary. Therefore, all previous frameworks earn ○ in the “Non-marginal energy service price changes” row.

Table 2 shows that previous frameworks contain many key pieces, providing starting points from which to develop our rebound analysis framework. A left-to-right reading of the table demonstrates that previous frameworks start from microeconomic consumer theory and move towards more rigorous theoretical treatment over time, with recent frameworks making important advanced theoretical contributions at the expense of empirical applicability. In the end, no previous rebound analysis framework combines all rebound effects across energy, expenditure, and consumption aspects with a detailed model of consumer preferences, non-marginal energy service price changes, and empirical applicability for the simplest case (understandable across disciplines) of a single fuel and a single energy service. In particular, assessing the rebound implications of differential capital costs, non-marginal price changes, and the macro effect required conceptual development as in Section 2.5.4 and Appendix B.4.5. (Development of empirical applications is left for Heun et al. (2025).) This paper addresses most of the gaps in Table 2; hence we fill the “This paper (2025)” column with filled circles (•) in nearly all rows. By so doing, we help advance clarity in the field of energy rebound.

### 3.2. Notes on an Energy Price Rebound Effect

The income effect (Section 2.5.3) captures the energy and rebound implications of expanding real income at the level of the upgraded device. The partial equilibrium framework described herein enables calculation of income effect rebound ( $Re_{\mathrm{inc}}$ ) without regard to changes in energy price ( $p_{E}$ ), because the energy price is assumed exogenous.

But there are other effects at work beyond the device level and outside the boundaries of a partial equilibrium analysis. One of those effects is an energy price effect. This section (and Appendix F) shows that our partial equilibrium framework can be extended to obtain an initial estimate of the rebound implications of an energy price effect ( $Re_{p_{E}}$ ) with an analysis that remains short of full equilibrium.

The energy price effect can lead to rebound when EEUs are applied to energy conversion devices at a scale that is substantial relative to the economy-wide use of energy. Examples of conditions under which the energy price effect could be significant include replacing all cars in the economy by hybrids and replacing all domestic electric lamps in the economy by LEDs, to use the examples from Heun et al. (2025). With reduced energy demand throughout the economy, an energy price reduction can be expected ( $p_{E}^{{}^{\circ}}\;>\;{\bar{p}}_{E}$ ) as the lower energy price leads to rebalancing of supply and demand. With the now-lower energy price ( ${\bar{p}}_{E}$ ), the device owner has additional freed cash ( ${\overset{\cdot }{G}}_{p_{E}}$ ) to spend, in addition to the adjustments described by the substitution and income effects (see Sections 2.5.2 and 2.5.3).

A complete analysis of the price effect would amount to introducing a full model of the energy market and involve solving a system of simultaneous equations for the new economy-wide energy demand, the new energy price, and a new consumption bundle. But in this instance, as we desire a simple estimate of energy price rebound, we conservatively assume the device owner spends the additional freed cash (the result of the lower energy price) exclusively on other goods, with energy implications at the energy intensity of the economy ( $I_{E}$ ). Under these assumptions, Appendix F derives an expression for rebound from the energy price effect as

(36) $$Re_{p_{E}}=\frac{{\overset{\cdot }{G}}_{p_{E}}I_{E}}{{\overset{\cdot }{S}}_{\mathrm{dev}}}\;,$$

where ${\overset{\cdot }{G}}_{p_{E}}$ is the freed cash arising from the reduction in energy price due to widespread adopotion of the EEU throughout the economy.

## 4. Conclusions

In this paper (Part I), we developed foundations of a rigorous analytical framework that includes all rebound effects across energy, expenditure, and consumption aspects with a detailed model of consumer preferences and non-marginal energy service price changes in an operational manner linking micro and macro effects for the simplest case of a single fuel and a single energy service. Furthermore, we presented approaches for exploring consumer satiation of energy service demand and for analyzing the effect of reduced energy demand on energy price to create energy price rebound. With careful explication of rebound effects and clear derivation of rebound expressions, we help advance the analytical foundations for empirical analyses and facilitate interdisciplinary understanding of rebound phenomena toward the goal of enhancing clarity in the field of energy rebound and enabling more robust rebound calculations for sound energy and climate policy.

Future work could be pursued in several areas. (i) Other utility models (besides the CES utility model, but not a Cobb-Douglas utility model) could be explored for the substitution effect. (ii) Although this is a consumer-sided framework, we demonstrated that it could be extended to effects affecting producers such as the energy price rebound effect. Further work could explore additional extensions to other producer-sided energy rebound effects. Moreover, a neoclassical producer framework, in analogy to the consumer framework, could be derived due to the substantial symmetry between neoclassical consumer and producer models (Fine 2016; Varian 1992). (iii) This framework could be extended to include some of the advanced topics in Chan and Gillingham (2015) and Wang, Yu and Liu (2021), such as multiple fuels or energy services, more than one other consumption good, and nested utility functions with intermediate inputs. (iv) This framework could be extended to include fuel-switching EEUs, wherein the upgraded device uses a different fuel from the original device. (v) The greenhouse gas emissions implications of energy rebound could be evaluated using this framework, provided that the primary energy associated with final energy purchases were available. Borenstein (2015) went some way to analyzing emissions and could provide a starting point for such work. The capability to analyze fuel-switching EEUs (discussed in the previous item) will be important for analyzing the greenhouse gas emissions implications of many EEUs that involve electrification, such as the transition to all-electric vehicles and the conversion of natural gas and oil furnaces to heat pumps for home heating.

In Heun et al. (2025), we further help advance clarity in rebound analysis in three ways. First, we develop a way to visualize the energy, expenditure, and consumption aspects of rebound effects. Second, we apply the framework to two EEUs: an upgraded car and an upgraded electric lamp. Finally, we provide results of rebound calculations for the two examples.

## Footnotes

1 We prefer the term “energy analysts” over “engineers,” because “energy analysts” better describes the group of people engaged in “energy analysis.” For this paper, we define “energy analysis” to be the study of energy transformations from stocks to flows and wastes along society’s energy conversion chain for the purpose of generating energy services, economic activity, and human well-being.

2 Indeed, this is why the authors for these papers come from the disciplines of energy analysis (MKH, PEB) and economics (GS).

3 This objective may mean that some aspects of the development of the framework will seem obvious to energy analysts while other aspects will seem obvious to economists.

4 For example, Sorrell (2009) sets out five macroeconomic rebound effects: embodied energy effects, responding effects, output effects, energy market effects, and composition effects. (We place the embodied energy effect at the microeconomic level.) Santarius (2016) and Lange et al. (2021) introduce meso (i.e., sectoral) level rebound between the micro and macro levels. van den Bergh (2011) distinguishes 14 types of rebound, providing, perhaps, the greatest complexity.

5 Note that the vocabulary and mathematical notation for rebound effects is important; Figure 1 and Appendix A provide guides to elements used throughout this paper, including symbols, Greek letters, abbreviations, decorations, and subscripts. The notational elements can be mixed to provide a rich and expressive symbolic “language” for energy rebound. As the goal of this paper is to bridge disciplines, the nomenclature will necessarily have unfamiliar elements to each discipline involved.

6 Note that the takeback rate can be negative, indicating that the actual final energy savings rate is greater than the expected final energy savings rate, a condition called hyperconservation.

7 Conventionally, stages of the energy conversion chain are primary energy (e.g., coal, oil, natural gas, wind, and solar), final energy (e.g., electricity and refined petroleum), useful energy (e.g., heat, light, and mechanical drive), and energy services (e.g., transport, illumination, and space heating). See Sousa et al. (2017) for an introduction to societal energy and exergy accounting.

8 Primary energy may be important when the upgraded device consumes a different final energy carrier compared to the original device, that is, when fuel-switching occurs (Chan and Gillingham 2015).

9 We discount money because interest changes the available amount of money over time. In contrast, we do not discount energy, because there is no temporal variation in the ability of energy to effect changes (via heat or work) in the physical world. We thank an anonymous reviewer for the insight that, in principle, the carbon content of energy could also be discounted if one assumes that near term emissions are worse than later emissions.

10 Relaxing the exogenous energy price assumption would require a general equilibrium model that is beyond the scope of this paper. However, see Section 3.2 where we discuss an energy price rebound effect as an extension of this framework.

11 Note that “pass” is short for “passenger,” and “lm” is the SI notation for the lumen, a unit of lighting energy rate.

12 Of course, it is often the case that the original and upgraded devices have small performance differences. For example, a high-efficiency LED lamp may have slightly greater or slightly lesser lumen output than the incandescent lamp it replaces. For the purpose of explicating this framework, we assume that the performance of the upgraded device can be matched closely enough to the performance of the original device such that the differences are immaterial to the user.

13 We take an energy approach here, consistent with the literature on energy rebound. One could use an alternative quantification of energy, such as exergy, the work potential of energy (Sciubba and Wall 2007) or emergy, the solar content of energy (Brown and Herendeen 1996).

14 For the original development of the decomposition see Slutsky (1915) and Allen (1936). For a modern introduction see Nicholson and Snyder (2017).

15 Alternative assumptions on behavior would arise from, for example, adopting a behavioral economic framework (Dorner 2019; Dütschke et al. 2018) or an informational entropy-constrained economic framework (Foley 2020).

16 Other utility models could be used; however, the Cobb-Douglas utility model is inappropriate for this framework, because it assumes that the sum of substitution and income rebound is 100% *always*. Regardless of the utility model, expressions for ${\hat{\overset{\cdot }{q}}}_{s}/{\overset{\cdot }{q}}_{s}^{{}^{\circ}}$ and ${\hat{\overset{\cdot }{C}}}_{g}/{\overset{\cdot }{C}}_{g}^{{}^{\circ}}$ must be determined and substituted into equations (18) and (20), respectively.

17 In principle, the energy service could be an “inferior good” whose consumption declines as incomes rise. However, energy service elasticities of income have been estimated to be positive over the long run, so we do not expect the inferior good case to be relevant (Fouquet 2014).

18 Zero net savings ( $\hat{\overset{\cdot }{N}}=0$ ) could occur if increases in the capital cost rate ( $\Delta {\overset{\cdot }{C}}_{\mathrm{cap}}^{*}$ ) and/or the operations, maintenance, and disposal cost rate ( $\Delta {\overset{\cdot }{C}}_{\mathrm{OMd}}^{*}$ ) consume all freed cash ( $\overset{\cdot }{G}$ ) plus savings from the compensating variation.

19 To appreciate the difference between production for the market and production for the household, consider the example of increased fuel efficiency. In one case, the household upgrades its own car and takes more trips (direct rebound), without effect on GDP. In the other case, the household buys the energy service (transport) directly from a taxi company that upgrades its cars. Here, the taxi company lowers the price but gains more customers, leading immediately to growth in inflation-adjusted (i.e., real) GDP, as more driving services are produced. Yet, the physical change of more car trips is the same in both cases.

20 Nevertheless, as long as the energy efficiency improvement (in this example, an upgraded car) is the only technological progress in the economy, further output growth may be constrained to the extent that the other inputs into production remain constrained at their original levels and substituting energy for the other inputs to production is limited by the prevailing technology.

21 It is important to distinguish this multiplier from an autonomous expansion of expenditure, a demand-side shock, in an otherwise unchanged economy, that is, the Keynesian multiplier (Kahn 1931; Keynes 1936), that risks crowding out other economic activity (Gillingham, Rapson and Wagner 2016). Our energy productivity improvement is a supply-side shock. After the EEU, it takes less energy (and therefore less energy cost) to generate the same economic activity, because energy efficiency has improved, so the concept of crowding-out as defined by macroeconomics does not apply.

22 The macro factor ( k ) appears unitless, but its units are actually $ of economy-wide expansion created per $ of net savings gained by the device user in the emplacement effect ( ${\overset{\cdot }{N}}^{*}$ ).

23 General equilibrium frameworks provide detail and precision on economy-wide price adjustments, but they give up specificity about individual device upgrades, make assumptions during calibration, and lose simplicity of exposition.

24 See Section 2.2 of Heun et al. (2025) for literal pictures of rebound in energy, expenditure, and consumption planes.

## Appendices

### A. Nomenclature

Presentation of the rigorous analytical framework is aided by a nomenclature that describes energy stages and rebound effects. Table A.1 shows symbols and abbreviations, their meanings, and example units. Table A.2 shows Greek letters, their meanings, and example units. Table A.3 shows initialisms. Table A.4 shows symbol decorations and their meanings. Table A.5 shows subscripts and their meanings.

**Table A.1.** Symbols and Abbreviations.

Symbol	Meaning [example units]
A	Annualized cost [$/yr]
a	The share parameter in the CES utility model [–]
C	Cost [$]
E	Final energy [MJ]
f	Expenditure share [–]
G	Freed cash [$]
g	A constant in the derivation of $\varepsilon_{{\overset{\cdot }{q}}_{s},p_{s},c}$ and $\varepsilon_{{\overset{\cdot }{q}}_{g},p_{s},c}$ . [–]
h	A constant in the derivation of $\varepsilon_{{\overset{\cdot }{q}}_{s},p_{s},c}$ and $\varepsilon_{{\overset{\cdot }{q}}_{g},p_{s},c}$ [–]
I	Energy intensity of economic activity [MJ/$]
i	Summation index for present value calculations [–]
k	Macro factor [–]
M	Income [$]
m	An exponent in the derivation of $\varepsilon_{{\overset{\cdot }{q}}_{s},p_{s},c}$ and $\varepsilon_{{\overset{\cdot }{q}}_{g},p_{s},c}$ [–]
N	Net savings [$]
n	An exponent in the derivation of $\varepsilon_{{\overset{\cdot }{q}}_{s},p_{s},c}$ and $\varepsilon_{{\overset{\cdot }{q}}_{g},p_{s},c}$ [–]
P	Present value [$]
p	Price [$]
Q	Quantity at the macroeconomic level [–]
q	Quantity [–]
Re	Rebound [–]
r	Real monetary discount rate [1/yr]
S	Energy cost savings [$]
t	Time variable [yr]
u	Utility [utils]
x	Position [m]
z	A constant in the derivation of $\varepsilon_{{\overset{\cdot }{q}}_{s},p_{s},c}$ and $\varepsilon_{{\overset{\cdot }{q}}_{g},p_{s},c}$ [–]

**Table A.2.** Greek Letters.

Greek letter	Meaning [example units]
α	Subscript that indicates capital cost payments at beginning of life
Δ	Difference (later quantity less earlier quantity, see Figure 1)
ε	Price or income elasticity [–]
$\varepsilon_{{\overset{\cdot }{q}}_{s},\overset{\cdot }{M}}$	Income ( $\overset{\cdot }{M}$ ) elasticity of energy service demand ( ${\overset{\cdot }{q}}_{s}$ ) [–]
$\varepsilon_{{\overset{\cdot }{q}}_{g},\overset{\cdot }{M}}$	Income ( $\overset{\cdot }{M}$ ) elasticity of other goods demand ( ${\overset{\cdot }{q}}_{g}$ ) [–]
$\varepsilon_{{\overset{\cdot }{q}}_{s},p_{s}}$	Uncompensated energy service price ( $p_{s}$ ) elasticity of energy service demand ( ${\overset{\cdot }{q}}_{s}$ ) [–]
$\varepsilon_{{\overset{\cdot }{q}}_{g},p_{s}}$	Uncompensated energy service price ( $p_{s}$ ) elasticity of other goods demand ( ${\overset{\cdot }{q}}_{g}$ ) [–]
$\varepsilon_{{\overset{\cdot }{q}}_{s},p_{s},c}$	Compensated energy service price ( $p_{s}$ ) elasticity of energy service demand ( ${\overset{\cdot }{q}}_{s}$ ) [–]
$\varepsilon_{{\overset{\cdot }{q}}_{g},p_{s},c}$	Compensated energy service price ( $p_{s}$ ) elasticity of other goods demand ( ${\overset{\cdot }{q}}_{g}$ ) [–]
η	Final-energy-to-service efficiency [vehicle-km/MJ]
γ	Term in the derivation of end-of-life payment discounting [–]
ω	Subscript that indicates disposal cost at end of life
ϕ	Term in the derivation of beginning-of-life payment discounting [–]
ρ	Exponent in the CES utility function, ρ≡(σ−1)/σ [–]
σ	Elasticity of substitution between the energy service ( ${\overset{\cdot }{q}}_{s}^{{}^{\circ}}$ ) and other goods ( ${\overset{\cdot }{q}}_{o}^{{}^{\circ}}$ ) [–]
τ	Multiplicative term that accounts for discounting [–]

**Table A.3.** Initialisms.

Initials	Meaning
CES	Constant elasticity of substitution
CPE	Constant price elasticity
CV	Compensating variation
EEU	Energy efficiency upgrade
EPSRC	Engineering and Physical Sciences Research Council
GDP	Gross domestic product
UK	United Kingdom
UKRI	UK Research and Innovation
U.S.	United States

**Table A.4.** Decorations.

Decoration	Meaning [example units]
$X^{{}^{\circ}}$	X originally (before the emplacement effect)
$X^{*}$	X after the emplacement effect (before the substitution effect)
$\hat{X}$	X after the substitution effect (before the income effect)
$\bar{X}$	X after the income effect (before the macro effect)
$\tilde{X}$	X after the macro effect
$\overset{\cdot }{X}$	Rate of X [units of X/yr]
$M^{'}$	Effective income [$]

**Table A.5.** Subscripts.

Subscript	Meaning
c	Compensated
cap	Capital costs
dev	Device
dempl	Direct emplacement effect
d	Disposal
dinc	Direct income effect
dsub	Direct substitution effect
E	Energy
emb	Embodied
empl	Emplacement effect
g	Other expenditures (besides energy) by the device user
iempl	Indirect emplacement effects
iinc	Indirect income effect
inc	Income effect
isub	Indirect substitution effect
life	Lifetime
m	Maintenance
macro	Macro effect
OM	Operations and maintenance
OMd	Operations, maintenance, and disposal
s	Service stage of the energy conversion chain
sub	Substitution effect
tot	Sum of all rebound effects in the framework

Differences are indicated by the Greek letter Δ and always signify subtraction of a quantity at an earlier stage of Figure 1 from the same quantity at the next later stage of Figure 1. For example, $\Delta \bar{X}\;\equiv \;\bar{X}-\hat{X}$ , and $\Delta \tilde{X}\;\equiv \;\tilde{X}-\bar{X}$ . Lack of decoration on a difference term indicates a difference that spans all stages of Figure 1. For example, $\Delta X\;\equiv \;\tilde{X}-X^{{}^{\circ}}$ . ΔX is also the sum of differences across each stage in Figure 1, as shown below.

(37)
ΔX=ΔX~+ΔX¯+ΔX^+ΔX∗ΔX=(X~−X¯)+(X¯−X^)+(X^−X∗)+(X∗−X°)ΔX=(X~−X¯)+(X¯−X^)+(X^−X∗)+(X∗−X°)ΔX=X~−X°

### B. Derivation of the Analytical Framework

This appendix provides a detailed derivation of the analytical framework, beginning with the budget constraint for the device owner.

#### B.1. Budget Constraint

We assume the device owner has four expense categories related to the device: capital cost ( $C_{\mathrm{cap}}$ ), energy service cost ( $C_{s}$ ), operations and maintenance cost ( $C_{\mathrm{OM}}$ ), and disposal cost ( $C_{d}$ ). We count one expense category for all other goods and services ( $C_{g}$ ), one category for annual income ( M ), and net savings ( N ), the difference between income and expenses. Capital ( cap ) and disposal ( d ) costs are applied at the beginning ( α ) and end ( ω ), respectively, of the device lifetime ( $t_{\mathrm{life}}$ ). All other budget categories are applied at the beginning of each year. A budget can be constructed for the device owner for each stage of Figure 1, leading to a different budget before emplacement (°), after emplacement (*), after the substitution effect (∧), after the income effect (−), and after the macro effect (~). When needed, the different budgets can be distinguished by symbol decorations shown in Table A.4. We allow the device owner to purchase the device with a loan and assume a real discount rate r . For a device not purchased on credit, r=0 applies. The device owner may save (with real discount rate r ) to pay for future disposal costs.

Each budget category is analyzed in perpetuity to allow comparisons at different rebound stages (°, *, etc.) where the device lifetime ( $t_{\mathrm{life}}$ ) may be different. The present value ( P ) of each expense category is obtained with an infinite sum for three cases.

First, the present value ( $P_{\mathrm{cap}}$ ) of the capital cost ( $C_{\mathrm{cap}}$ ) is given by the infinite sum

(38)
$$\begin{array}{cc} P_{\mathrm{cap}} & =C_{\mathrm{cap}}+\frac{C_{\mathrm{cap}}}{{\left(1+r\right)}^{t_{\mathrm{life}}}}+\frac{C_{\mathrm{cap}}}{{\left(1+r\right)}^{2t_{\mathrm{life}}}}+\ldots +\frac{C_{\mathrm{cap}}}{{\left(1+r\right)}^{i\;t_{\mathrm{life}}}}+\ldots \\ =C_{\mathrm{cap}}\sum_{i=0}^{\infty }\frac{1}{{\left(1+r\right)}^{i\;t_{\mathrm{life}}}}\\ =\phi_{t_{\mathrm{life}}}C_{\mathrm{cap}}, \end{array}$$

where $\phi_{t}\;\equiv \;\frac{{\left(1+r\right)}^{t}}{{\left(1+r\right)}^{t}-1}$ .

Second, the present value of all yearly expenses or income can be given by similar equations. For the example of the present value ( $P_{s}$ ) of annual energy services costs ( $C_{s}$ ), we have

(39)
$$\begin{array}{c} P_{s}=C_{s}+\frac{C_{s}}{{\left(1+r\right)}^{1\;\mathrm{yr}}}+\frac{C_{s}}{{\left(1+r\right)}^{2\;\mathrm{yr}}}+\ldots +\frac{C_{s}}{{\left(1+r\right)}^{i\;\mathrm{yr}}}+\ldots \\ =C_{s}\sum_{i=0}^{\infty }\frac{1}{{\left(1+r\right)}^{i\;\mathrm{yr}}}\\ =\phi_{1\;\mathrm{yr}}C_{s}. \end{array}$$

Equations for the present value of annual operations and maintenance costs ( $P_{\mathrm{OM}}$ and $C_{\mathrm{OM}}$ ), annual other goods costs ( $P_{g}$ and $C_{g}$ ), annual income ( $P_{M}$ and M ), and annual net savings ( $P_{N}$ and N ) are identical except for the subscripts.

Finally, the present value ( $P_{d}$ ) of disposal costs ( $C_{d}$ ) is given by

(40)
$$\begin{array}{cc} P_{d} & =\frac{C_{d}}{{\left(1+r\right)}^{t_{\mathrm{life}}}}+\frac{C_{d}}{{\left(1+r\right)}^{2t_{\mathrm{life}}}}+\ldots +\frac{C_{d}}{{\left(1+r\right)}^{i\;t_{\mathrm{life}}}}+\ldots \\ =C_{d}\sum_{i=1}^{\infty }\frac{1}{{\left(1+r\right)}^{i\;t_{\mathrm{life}}}}\\ =\gamma_{t_{\mathrm{life}}}C_{d}, \end{array}$$

where $\gamma_{t}\equiv \frac{1}{{\left(1+r\right)}^{t}-1}$ .

For simplicity, we desire annual values ( A ) with equivalent present value for each cost category. Using the capital cost to illustrate, we begin with the present value equivalence of the infinite series and annual costs:

(41)
$$P_{\mathrm{cap}}=P_{A_{\mathrm{cap}}}.$$

Substituting expressions for present values (*P*) gives

(42)
$$\phi_{t_{\mathrm{life}}}C_{\mathrm{cap}}=\phi_{1\;\mathrm{yr}}A_{\mathrm{cap}}.$$

Rearranging gives

(43)
$$A_{\mathrm{cap}}=\frac{\phi_{\mathrm{life}}}{\phi_{1\;\mathrm{yr}}}C_{\mathrm{cap}}.$$

Further, we desire annualized rates defined as $\overset{\cdot }{A}\;\equiv \;A/1\;\mathrm{yr}$ such that ${\overset{\cdot }{A}}_{\mathrm{cap}}=A_{\mathrm{cap}}/1\;\mathrm{yr}$ and ${\overset{\cdot }{C}}_{\mathrm{cap}}\;\equiv \;C_{\mathrm{cap}}/t_{\mathrm{life}}$ . Solving for $A_{\mathrm{cap}}$ and $C_{\mathrm{cap}}$ and substituting gives

(44)
$${\overset{\cdot }{A}}_{\mathrm{cap}}\left(1\;\mathrm{yr}\right)=\frac{\phi_{t_{\mathrm{life}}}}{\phi_{1\;\mathrm{yr}}}{\overset{\cdot }{C}}_{\mathrm{cap}}t_{\mathrm{life}}.$$

Defining $\tau_{\alpha }\;\equiv \;\frac{\phi_{t_{\mathrm{life}}}}{\phi_{1\;\mathrm{yr}}}\frac{t_{\mathrm{life}}}{1\;\mathrm{yr}}$ (with subscript α indicating payments at the beginning of each device lifetime) gives

(45)
$${\overset{\cdot }{A}}_{\mathrm{cap}}=\tau_{\alpha }{\overset{\cdot }{C}}_{\mathrm{cap}}.$$

Similar derivations can be employed for all other budget categories to obtain

(46)
$${\overset{\cdot }{A}}_{s}={\overset{\cdot }{C}}_{s}$$

(47)
$${\overset{\cdot }{A}}_{\mathrm{OM}}={\overset{\cdot }{C}}_{\mathrm{OM}}$$

(48)
$${\overset{\cdot }{A}}_{d}=\tau_{\omega }{\overset{\cdot }{C}}_{d}$$

(49)
$${\overset{\cdot }{A}}_{g}={\overset{\cdot }{C}}_{g}$$

(50)
$${\overset{\cdot }{A}}_{N}=\overset{\cdot }{N}$$

(51)
$${\overset{\cdot }{A}}_{M}=\overset{\cdot }{M},$$

where $\tau_{\omega }\;\equiv \;\frac{\gamma_{t_{\mathrm{life}}}}{\phi_{1\;\mathrm{yr}}}\frac{t_{\mathrm{life}}}{1\;\mathrm{yr}}$ (with subscript ω indicating payments at the end of each device lifetime), and ${\overset{\cdot }{C}}_{d}\;\equiv \;C_{d}/t_{\mathrm{life}}$ , the annualized disposal cost without discounting.

The budget constraint expressed in annualized present-value equivalent terms is

(52)
$${\overset{\cdot }{A}}_{M}={\overset{\cdot }{A}}_{\mathrm{cap}}+{\overset{\cdot }{A}}_{s}+{\overset{\cdot }{A}}_{\mathrm{OM}}+{\overset{\cdot }{A}}_{d}+{\overset{\cdot }{A}}_{g}+{\overset{\cdot }{A}}_{N}.$$

Substituting cost rates gives

(53)
$$\overset{\cdot }{M}=\tau_{\alpha }{\overset{\cdot }{C}}_{\mathrm{cap}}+{\overset{\cdot }{C}}_{s}+{\overset{\cdot }{C}}_{\mathrm{OM}}+\tau_{\omega }{\overset{\cdot }{C}}_{d}+{\overset{\cdot }{C}}_{g}+\overset{\cdot }{N}.$$

Substituting the product of energy price ( $p_{E}$ ) and the rate of energy consumption (given by the ratio of the rate of energy service consumption and efficiency, ${\overset{\cdot }{q}}_{s}/\eta$ ), the product of price ( $p_{g}$ ) and the rate ( ${\overset{\cdot }{q}}_{g}$ ) of other goods consumption, ${\overset{\cdot }{C}}_{\mathrm{OMd}}^{{}^{\circ}}\;\equiv \;{\overset{\cdot }{C}}_{\mathrm{OM}}^{{}^{\circ}}+\tau_{\omega }^{{}^{\circ}}C_{d}^{{}^{\circ}}$ , and after some rearranging gives the budget constraint used in equation (5):

(54)
$$\overset{\cdot }{M}-\tau_{\alpha }{\overset{\cdot }{C}}_{\mathrm{cap}}-{\overset{\cdot }{C}}_{\mathrm{OMd}}=p_{E}\frac{{\overset{\cdot }{q}}_{s}}{\eta }+p_{g}{\overset{\cdot }{q}}_{g}+\overset{\cdot }{N}.$$

The term $\tau_{\alpha }$ represents the additional cost of annual interest payments when the device is purchased with a loan. When r>0 , $\tau_{\alpha }\;>\;1$ . When r=0 , $\tau_{\alpha }=1$ , as proved below (Section B.1.1). The term $\tau_{\omega }$ represents the reduction of disposal costs if the device owner pays for disposal costs with money invested annually assuming real discount rate r . When r>0 , $0\;<\;\tau_{\omega }\;<\;1$ . When r=0 , $\tau_{\omega }=1$ , as proved below (Section B.1.2).

##### B.1.1. Proof: $\tau_{\alpha }=1$ when r=0

We expect that $\tau_{\alpha }=1$ when r=0 . However, direct substitution of r=0 into the expression for $\tau_{\alpha }$ gives $\frac{0}{0}$ , so we rather assess $\underset{r\to 0^{\;+\;}}{\lim}\tau_{\alpha }\overset{?}{=1}$ .

Substituting for $\tau_{\alpha }$ gives

(55)
$$\underset{r\to 0^{+}}{\lim}\left(\frac{\phi_{t_{\mathrm{life}}}}{\phi_{1\;\mathrm{yr}}}\frac{t_{\mathrm{life}}}{1\;\mathrm{yr}}\right)\overset{?}{=}\;1.$$

Substituting for ϕ terms gives

(56)
$$\underset{r\to 0^{+}}{\lim}\left[\frac{\frac{{\left(1+r\right)}^{t_{\mathrm{life}}}}{{\left(1+r\right)}^{t_{\mathrm{life}}}-1}}{\frac{{\left(1+r\right)}^{1\;\mathrm{yr}}}{{\left(1+r\right)}^{1\;\mathrm{yr}}-1}}\cdot \frac{t_{\mathrm{life}}}{1\;\mathrm{yr}}\right]\overset{?}{=}\;1.$$

Distributing double-fractions gives

(57)
$$\underset{r\to 0^{+}}{\lim}\left[\frac{{\left(1+r\right)}^{t_{\mathrm{life}}}}{{\left(1+r\right)}^{1\;\mathrm{yr}}}\cdot \frac{{\left(1+r\right)}^{1\;\mathrm{yr}}-1}{{\left(1+r\right)}^{t_{\mathrm{life}}}-1}\cdot \frac{t_{\mathrm{life}}}{1\;\mathrm{yr}}\right]\overset{?}{=}\;1.$$

Multiplying terms in numerator and denominator gives

(58)
$$\underset{r\to 0^{+}}{\lim}\left\{\frac{\left[{\left(1+r\right)}^{t_{\mathrm{life}}}{\left(1+r\right)}^{1\;\mathrm{yr}}-{\left(1+r\right)}^{t_{\mathrm{life}}}\right]\frac{t_{\mathrm{life}}}{1\;\mathrm{yr}}}{{\left(1+r\right)}^{t_{\mathrm{life}}}{\left(1+r\right)}^{1\;\mathrm{yr}}-{\left(1+r\right)}^{1\;\mathrm{yr}}}\right\}\overset{?}{=}\;1.$$

Applying L’Hôpital’s rule gives

(59)
$$\underset{r\to 0^{+}}{\lim}\left(\frac{\frac{\partial }{\partial r}\left\{\left[{\left(1+r\right)}^{t_{\mathrm{life}}}{\left(1+r\right)}^{1\;\mathrm{yr}}-{\left(1+r\right)}^{t_{\mathrm{life}}}\right]\frac{t_{\mathrm{life}}}{1\;\mathrm{yr}}\right\}}{\frac{\partial }{\partial r}\left[{\left(1+r\right)}^{t_{\mathrm{life}}}{\left(1+r\right)}^{1\;\mathrm{yr}}-{\left(1+r\right)}^{1\;\mathrm{yr}}\right]}\right)\overset{?}{=}\;1.$$

Applying the chain rule repeatedly gives

(60)
$$\underset{r\to 0^{+}}{\lim}\left(\frac{\frac{t_{\mathrm{life}}}{1\;\mathrm{yr}}\left\{\frac{\partial }{\partial r}\left[{\left(1+r\right)}^{t_{\mathrm{life}}}{\left(1+r\right)}^{1\;\mathrm{yr}}\right]-\frac{\partial }{\partial r}\left[{\left(1+r\right)}^{t_{\mathrm{life}}}\right]\right\}}{\frac{\partial }{\partial r}\left[{\left(1+r\right)}^{t_{\mathrm{life}}}{\left(1+r\right)}^{1\;\mathrm{yr}}\right]-\frac{\partial }{\partial r}\left[{\left(1+r\right)}^{1\;\mathrm{yr}}\right]}\right)\overset{?}{=}\;1.$$

Several intermediate results are helpful.

(61)
$$\underset{r\to 0^{+}}{\lim}\left\{\frac{\partial }{\partial r}\left[{\left(1+r\right)}^{t_{\mathrm{life}}}\right]\right\}=t_{\mathrm{life}}$$

(62)
$$\underset{r\to 0^{+}}{\lim}\left\{\frac{\partial }{\partial r}\left[{\left(1+r\right)}^{1\;\mathrm{yr}}\right]\right\}=1\;\mathrm{yr}$$

(63)
$$\underset{r\to 0^{+}}{\lim}\left\{\frac{\partial }{\partial r}\left[{\left(1+r\right)}^{t_{\mathrm{life}}}{\left(1+r\right)}^{1\;\mathrm{yr}}\right]\right\}=t_{\mathrm{life}}{\left(1+r\right)}^{1\;\mathrm{yr}}+1\;\mathrm{yr}{\left(1+r\right)}^{t_{\mathrm{life}}}$$

Substituting the intermediate results gives

(64)
$$\underset{r\to 0^{+}}{\lim}\left\{\frac{\frac{t_{\mathrm{life}}}{1\;\mathrm{yr}}\left[{\left(1+r\right)}^{1\;\mathrm{yr}}\left(t_{\mathrm{life}}\right)+{\left(1+r\right)}^{t_{\mathrm{life}}}\left(1\;\mathrm{yr}\right)-t_{\mathrm{life}}\right]}{{\left(1+r\right)}^{1\;\mathrm{yr}}\left(t_{\mathrm{life}}\right)+{\left(1+r\right)}^{t_{\mathrm{life}}}\left(1\;\mathrm{yr}\right)-1\;\mathrm{yr}}\right\}{=}^{?}\;1.$$

Setting r=0 in the remaining terms gives

(65)
$$\frac{\frac{t_{\mathrm{life}}}{1\;\mathrm{yr}}\left[\left(1\right)\left(t_{\mathrm{life}}\right)+\left(1\right)\left(1\;\mathrm{yr}\right)-t_{\mathrm{life}}\right]}{\left(1\right)\left(t_{\mathrm{life}}\right)+\left(1\right)\left(1\;\mathrm{yr}\right)-1\;\mathrm{yr}}\overset{?}{=}\;1.$$

Simplifying gives

(66)
$$\frac{\left(\frac{t_{\mathrm{life}}}{1\;\mathrm{yr}}\right)\left(1\;\mathrm{yr}\right)}{t_{\mathrm{life}}}{=}^{?}\;1$$

(67)
$$1\overset{✓}{=}\;1,$$

thereby completing the proof with the expected result.

##### B.1.2. Proof: $\tau_{\omega }=1$ when r=0

We expect that $\tau_{\omega }=1$ when r=0 . However, direct substitution of r=0 into the expression for $\tau_{\omega }$ gives $\frac{0}{0}$ , so we rather assess $\lim_{r\to 0^{+}}\tau_{\omega }{=}^{?}\;1$ .

Substituting for $\tau_{\omega }$ gives

(68)
$$\underset{r\to 0^{+}}{\lim}\left(\frac{\gamma_{t_{\mathrm{life}}}}{\phi_{1\;\mathrm{yr}}}\frac{t_{\mathrm{life}}}{1\;\mathrm{yr}}\right)\overset{?}{=}\;1.$$

Substituting for γ and ϕ terms gives

(69)
$$\underset{r\to 0^{+}}{\lim}\left[\frac{\frac{1}{{\left(1+r\right)}^{t_{\mathrm{life}}}-1}}{\frac{{\left(1+r\right)}^{1\;\mathrm{yr}}}{{\left(1+r\right)}^{1\;\mathrm{yr}}-1}}\frac{t_{\mathrm{life}}}{1\;\mathrm{yr}}\right]\overset{?}{=}\;1.$$

Distributing double-fractions gives

(70)
$$\underset{r\to 0^{+}}{\lim}\left[\frac{1}{{\left(1+r\right)}^{1\;\mathrm{yr}}}\cdot \frac{{\left(1+r\right)}^{1\;\mathrm{yr}}-1}{{\left(1+r\right)}^{t_{\mathrm{life}}}-1}\cdot \frac{t_{\mathrm{life}}}{1\;\mathrm{yr}}\right]\overset{?}{=}\;1.$$

Multiplying terms in numerator and denominator gives

(71)
$$\underset{r\to 0^{+}}{\lim}\left\{\frac{\left[{\left(1+r\right)}^{1\;\mathrm{yr}}-1\right]\left(\frac{t_{\mathrm{life}}}{1\;\mathrm{yr}}\right)}{{\left(1+r\right)}^{t_{\mathrm{life}}}{\left(1+r\right)}^{1\;\mathrm{yr}}-{\left(1+r\right)}^{1\;\mathrm{yr}}}\right\}\overset{?}{=}\;1.$$

Applying L’Hôpital’s rule gives

(72)
$$\underset{r\to 0^{+}}{\lim}\left\{\frac{\frac{t_{\mathrm{life}}}{1\;\mathrm{yr}}\frac{\partial }{\partial r}\left[{\left(1+r\right)}^{1\;\mathrm{yr}}-1\right]}{\frac{\partial }{\partial r}\left[{\left(1+r\right)}^{t_{\mathrm{life}}}{\left(1+r\right)}^{1\;\mathrm{yr}}\right]-\frac{\partial }{\partial r}\left[{\left(1+r\right)}^{1\;\mathrm{yr}}\right]}\right\}\overset{?}{=}\;1.$$

Applying the intermediate results from Section B.1.1 yields

(73)
$$\underset{r\to 0^{+}}{\lim}\left[\frac{\left(\frac{t_{\mathrm{life}}}{1\;\mathrm{yr}}\right)\left(1\;\mathrm{yr}\right)}{{\left(1+r\right)}^{1\;\mathrm{yr}}\left(t_{\mathrm{life}}\right)+{\left(1+r\right)}^{t_{\mathrm{life}}}\left(1\;\mathrm{yr}\right)-1\;\mathrm{yr}}\right]\overset{?}{=}\;1.$$

Setting r=0 in the remaining terms gives

(74)
$$\frac{\left(\frac{t_{\mathrm{life}}}{1\;\mathrm{yr}}\right)\left(1\;\mathrm{yr}\right)}{\left(1\right)\left(t_{\mathrm{life}}\right)+\left(1\right)1\;\mathrm{yr}-1\;\mathrm{yr}}\overset{?}{=}\;1.$$

Simplifying the denominator gives

(75)
$$\frac{\left(\frac{t_{\mathrm{life}}}{1\;\mathrm{yr}}\right)\left(1\;\mathrm{yr}\right)}{t_{\mathrm{life}}}\overset{?}{=}\;1$$

(76)
$$1\overset{✓}{=}\;1,$$

thereby completing the proof with the expected result.

#### B.2. Relationships for Rebound Effects

For each energy rebound effect in Figure 1, energy and financial analysis must be performed. The purposes of the analyses are to determine for each effect (i) an expression for energy rebound ( Re ) for the effect and (ii) an equation for net savings ( $\overset{\cdot }{N}$ ) remaining after the effect.

Analysis of each rebound effect involves a set of assumptions and constraints as shown in Table B.1. In Table B.1, relationships for emplacement effect embodied energy rates ( ${\overset{\cdot }{E}}_{\mathrm{emb}}^{{}^{\circ}}$ and ${\overset{\cdot }{E}}_{\mathrm{emb}}^{*}$ ), capital expenditure rates ( ${\overset{\cdot }{C}}_{\mathrm{cap}}^{{}^{\circ}}$ and ${\overset{\cdot }{C}}_{\mathrm{cap}}^{*}$ ), and operations, maintenance, and disposal expenditure rates ( ${\overset{\cdot }{C}}_{\mathrm{OMd}}^{{}^{\circ}}$ and ${\overset{\cdot }{C}}_{\mathrm{OMd}}^{*}$ ) are typical, and inequalities could switch direction for a specific EEU. Macro effect relationships are given for a single device only. If the EEU is deployed at scale across the economy, the energy service consumption rate ( ${\tilde{\overset{\cdot }{q}}}_{s}$ ), device energy consumption rate ( ${\tilde{\overset{\cdot }{E}}}_{s}$ ), embodied energy rate ( ${\tilde{\overset{\cdot }{E}}}_{\mathrm{emb}}$ ), capital expenditure rate ( ${\tilde{\overset{\cdot }{C}}}_{\mathrm{cap}}$ ), and operations, maintenance, and disposal expenditure rate ( ${\tilde{\overset{\cdot }{C}}}_{\mathrm{OMd}}$ ) will all increase in proportion to the number of devices emplaced.

**Table B.1.** Assumptions and Constraints for Analysis of Rebound Effects.

Parameter	Emplacement effect	Substitution effect	Income effect	Macro effect
Energy price	$p_{E}^{{}^{\circ}}=p_{E}^{*}$	$p_{E}^{*}={\hat{p}}_{E}$	${\hat{p}}_{E}={\bar{p}}_{E}$	${\bar{p}}_{E}={\tilde{p}}_{E}$
Energy service efficiency	$\eta^{{}^{\circ}}\;<\;\eta^{*}$	$\eta^{*}=\hat{\eta }$	$\hat{\eta }=\bar{\eta }$	$\bar{\eta }=\tilde{\eta }$
Energy service price	$p_{s}^{{}^{\circ}}\;>\;p_{s}^{*}$	$p_{s}^{*}={\hat{p}}_{s}$	${\hat{p}}_{s}={\bar{p}}_{s}$	${\bar{p}}_{s}={\tilde{p}}_{s}$
Other goods price	$p_{g}$	$p_{g}$	$p_{g}$	$p_{g}$
Energy service consumption rate	${\overset{\cdot }{q}}_{s}^{{}^{\circ}}={\overset{\cdot }{q}}_{s}^{*}$	${\overset{\cdot }{q}}_{s}^{*}\;<\;{\hat{\overset{\cdot }{q}}}_{s}$	${\hat{\overset{\cdot }{q}}}_{s}\;<\;{\bar{\overset{\cdot }{q}}}_{s}$	${\bar{\overset{\cdot }{q}}}_{s}={\tilde{\overset{\cdot }{q}}}_{s}$
Other goods consumption rate	${\overset{\cdot }{q}}_{g}^{{}^{\circ}}={\overset{\cdot }{q}}_{g}^{*}$	${\overset{\cdot }{q}}_{g}^{*}\;>\;{\hat{\overset{\cdot }{q}}}_{g}$	${\hat{\overset{\cdot }{q}}}_{g}\;<\;{\bar{\overset{\cdot }{q}}}_{g}$	${\bar{\overset{\cdot }{q}}}_{g}={\tilde{\overset{\cdot }{q}}}_{g}$
Device energy consumption rate	${\overset{\cdot }{E}}_{s}^{{}^{\circ}}\;>\;{\overset{\cdot }{E}}_{s}^{*}$	${\overset{\cdot }{E}}_{s}^{*}\;<\;{\hat{\overset{\cdot }{E}}}_{s}$	${\hat{\overset{\cdot }{E}}}_{s}\;<\;{\bar{\overset{\cdot }{E}}}_{s}$	${\bar{\overset{\cdot }{E}}}_{s}={\tilde{\overset{\cdot }{E}}}_{s}$
Embodied energy rate	${\overset{\cdot }{E}}_{\mathrm{emb}}^{{}^{\circ}}\;<\;{\overset{\cdot }{E}}_{\mathrm{emb}}^{*}$	${\overset{\cdot }{E}}_{\mathrm{emb}}^{*}={\hat{\overset{\cdot }{E}}}_{\mathrm{emb}}$	${\hat{\overset{\cdot }{E}}}_{\mathrm{emb}}={\bar{\overset{\cdot }{E}}}_{\mathrm{emb}}$	${\bar{\overset{\cdot }{E}}}_{\mathrm{emb}}={\tilde{\overset{\cdot }{E}}}_{\mathrm{emb}}$
Device lifetime	$t_{\mathrm{life}}^{{}^{\circ}}\;<\;t_{\mathrm{life}}^{*}$	$t_{\mathrm{life}}^{*}={\hat{t}}_{\mathrm{life}}$	${\hat{t}}_{\mathrm{life}}={\bar{t}}_{\mathrm{life}}$	${\bar{t}}_{\mathrm{life}}={\tilde{t}}_{\mathrm{life}}$
Beginning-of-life discount factor	$\tau_{\alpha }^{{}^{\circ}}\;<\;\tau_{\alpha }^{*}$	$\tau_{\alpha }^{*}={\hat{\tau }}_{\alpha }$	${\hat{\tau }}_{\alpha }={\bar{\tau }}_{\alpha }$	${\bar{\tau }}_{\alpha }={\tilde{\tau }}_{\alpha }$
End-of-life discount factor	$\tau_{\omega }^{{}^{\circ}}\;>\;\tau_{\omega }^{*}$	$\tau_{\omega }^{*}={\hat{\tau }}_{\omega }$	${\hat{\tau }}_{\omega }={\bar{\tau }}_{\omega }$	${\bar{\tau }}_{\omega }={\tilde{\tau }}_{\omega }$
Capital expenditure rate	${\overset{\cdot }{C}}_{\mathrm{cap}}^{{}^{\circ}}\;<\;{\overset{\cdot }{C}}_{\mathrm{cap}}^{*}$	${\overset{\cdot }{C}}_{\mathrm{cap}}^{*}={\hat{\overset{\cdot }{C}}}_{\mathrm{cap}}$	${\hat{\overset{\cdot }{C}}}_{\mathrm{cap}}={\bar{\overset{\cdot }{C}}}_{\mathrm{cap}}$	${\bar{\overset{\cdot }{C}}}_{\mathrm{cap}}={\tilde{\overset{\cdot }{C}}}_{\mathrm{cap}}$
Ops., maint., and disp. expenditure rate	${\overset{\cdot }{C}}_{\mathrm{OMd}}^{{}^{\circ}}\;<\;{\overset{\cdot }{C}}_{\mathrm{OMd}}^{*}$	${\overset{\cdot }{C}}_{\mathrm{OMd}}^{*}={\hat{\overset{\cdot }{C}}}_{\mathrm{OMd}}$	${\hat{\overset{\cdot }{C}}}_{\mathrm{OMd}}={\bar{\overset{\cdot }{C}}}_{\mathrm{OMd}}$	${\bar{\overset{\cdot }{C}}}_{\mathrm{OMd}}={\tilde{\overset{\cdot }{C}}}_{\mathrm{OMd}}$
Energy service expenditure rate	${\overset{\cdot }{C}}_{s}^{{}^{\circ}}\;>\;{\overset{\cdot }{C}}_{s}^{*}$	${\overset{\cdot }{C}}_{s}^{*}\;<\;{\hat{\overset{\cdot }{C}}}_{s}$	${\hat{\overset{\cdot }{C}}}_{s}\;<\;{\bar{\overset{\cdot }{C}}}_{s}$	${\bar{\overset{\cdot }{C}}}_{s}={\tilde{\overset{\cdot }{C}}}_{s}$
Other goods expenditure rate	${\overset{\cdot }{C}}_{g}^{{}^{\circ}}={\overset{\cdot }{C}}_{g}^{*}$	${\overset{\cdot }{C}}_{g}^{*}\;>\;{\hat{\overset{\cdot }{C}}}_{g}$	${\hat{\overset{\cdot }{C}}}_{g}\;<\;{\bar{\overset{\cdot }{C}}}_{g}$	${\bar{\overset{\cdot }{C}}}_{g}={\tilde{\overset{\cdot }{C}}}_{g}$
Income	$\overset{\cdot }{M}$	$\overset{\cdot }{M}$	$\overset{\cdot }{M}$	$\overset{\cdot }{M}$
Net savings	$0\;=\;{\overset{\cdot }{N}}^{{}^{\circ}}\;<\;{\overset{\cdot }{N}}^{*}$	${\overset{-}{N}}^{*}<\hat{\dot{N}}$	$\hat{\overset{\cdot }{N}}\;>\;\bar{\overset{\cdot }{N}}=0$	$\bar{\overset{\cdot }{N}}=\tilde{\overset{\cdot }{N}}=0$

**Table B.2.** Justification for Zeroed Terms in Tables B.3–B.6.

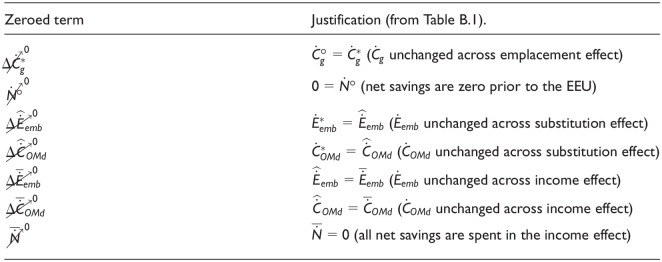

**Table B.3.** Emplacement Effect.

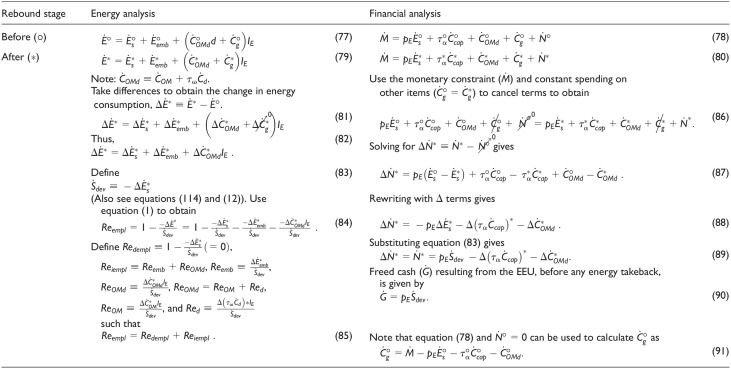

**Table B.4.** Substitution Effect.

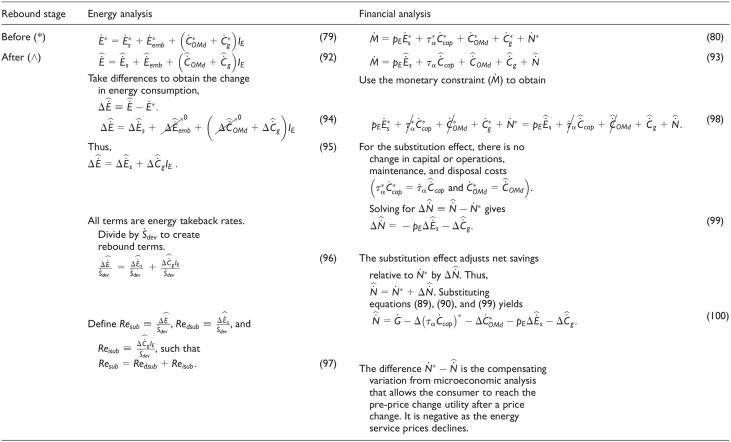

**Table B.5.** Income Effect.

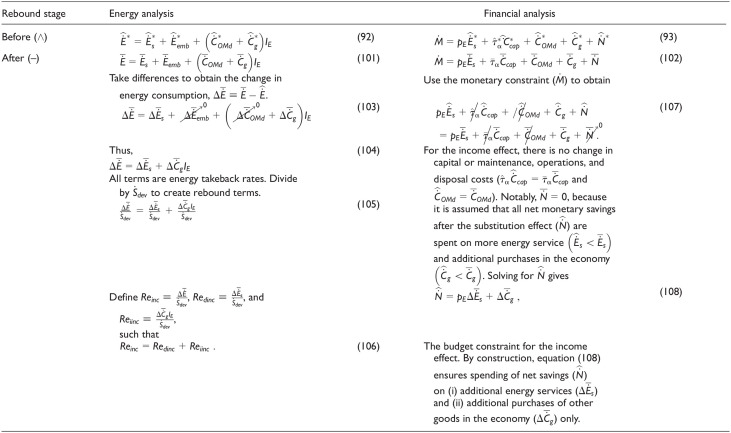

**Table B.6.** Macro Effect.

Rebound stage	Energy analysis		Financial analysis
Before (–)	$\bar{\overset{\cdot }{E}}$	(109)	
After (~)	$\tilde{\overset{\cdot }{E}}$	(110)	
	Take differences to obtain the change in energy consumption,		N/A
	$\Delta \tilde{\overset{\cdot }{E}}\;\equiv \;\tilde{\overset{\cdot }{E}}-\bar{\overset{\cdot }{E}}.$	(111)	
	The energy change due to the macro effect ( $\Delta \tilde{\overset{\cdot }{E}}$ ) is a scalar multiple ( k ) of net savings ( ${\overset{\cdot }{N}}^{*}$ ), assumed to be spent at the energy intensity of the economy ( $I_{E}$ ).		
	$\Delta \tilde{\overset{\cdot }{E}}={\overset{\cdot }{N}}^{*}I_{E}$	(112)	
	All terms are energy takeback rates. Divide by ${\overset{\cdot }{S}}_{\mathrm{dev}}$ to create rebound terms.		
	$\frac{\Delta \tilde{\overset{\cdot }{E}}}{{\overset{\cdot }{S}}_{\mathrm{dev}}}=\frac{k{\overset{\cdot }{N}}^{*}I_{E}}{{\overset{\cdot }{S}}_{\mathrm{dev}}}$	(113)	
	Define $Re_{\mathrm{macro}}\;\equiv \;\frac{\Delta \tilde{\overset{\cdot }{E}}}{{\overset{\cdot }{S}}_{\mathrm{dev}}}$ , such that		
	$Re_{\mathrm{macro}}=\frac{k{\overset{\cdot }{N}}^{*}I_{E}}{{\overset{\cdot }{S}}_{\mathrm{dev}}}\;.$	(33)	

#### B.3. Derivations

Table B.2 highlights equations in Table B.1 that justify zeroing terms in tables that follow. Derivations for rebound definitions and net savings equations are presented in Tables B.3 to B.6, one for each rebound effect in Figure 1. Energy and financial analyses are shown side by side, because each informs the other.

#### B.4. Rebound Expressions

All that remains is to determine expressions for each rebound effect. We begin with the device-level expected energy savings rate ( ${\overset{\cdot }{S}}_{\mathrm{dev}}$ ), which appears in the denominator of all rebound expressions.

##### B.4.1. Expected energy savings ( ${\overset{\cdot }{S}}_{\mathrm{dev}}$ )

${\overset{\cdot }{S}}_{\mathrm{dev}}$ is the reduction of energy consumption rate by the device due to the EEU. No other effects are considered.

(114)
$${\overset{\cdot }{S}}_{\mathrm{dev}}\;\equiv \;{\overset{\cdot }{E}}_{s}^{{}^{\circ}}-{\overset{\cdot }{E}}_{s}^{*}$$

The final energy consumption rates ( ${\overset{\cdot }{E}}_{s}^{{}^{\circ}}$ and ${\overset{\cdot }{E}}_{s}^{*}$ ) can be written as equation (6) in the forms ${\overset{\cdot }{E}}_{s}^{{}^{\circ}}={\overset{\cdot }{q}}_{s}^{{}^{\circ}}/\eta^{{}^{\circ}}$ and ${\overset{\cdot }{E}}_{s}^{*}={\overset{\cdot }{q}}_{s}^{*}/\eta^{*}$ .

(115)
$${\overset{\cdot }{S}}_{\mathrm{dev}}=\frac{{\overset{\cdot }{q}}_{s}^{{}^{\circ}}}{\eta^{{}^{\circ}}}-\frac{{\overset{\cdot }{q}}_{s}^{*}}{\eta^{*}}$$

With reference to Table B.1, we use ${\overset{\cdot }{q}}_{s}^{*}={\overset{\cdot }{q}}_{s}^{{}^{\circ}}$ to obtain

(116)
$${\overset{\cdot }{S}}_{\mathrm{dev}}=\frac{{\overset{\cdot }{q}}_{s}^{{}^{\circ}}}{\eta^{{}^{\circ}}}-\frac{{\overset{\cdot }{q}}_{s}^{{}^{\circ}}}{\eta^{*}}.$$

When the EEU increases efficiency such that $\eta^{{}^{\circ}}\;<\;\eta^{*}$ , expected energy savings grows ( ${\overset{\cdot }{S}}_{\mathrm{dev}}\;>\;0$ ) as the rate of final energy consumption declines, as expected. As $\eta^{*}\to \infty$ , all final energy consumption is eliminated ( ${\overset{\cdot }{E}}_{s}^{*}\to 0$ ), and ${\overset{\cdot }{S}}_{\mathrm{dev}}={\overset{\cdot }{q}}_{s}^{{}^{\circ}}/\eta^{{}^{\circ}}={\overset{\cdot }{E}}_{s}^{{}^{\circ}}$ . (Of course, $\eta^{*}\to \infty$ is impossible. See Paoli and Cullen (2020) for a discussion of upper limits to device efficiencies.)

After rearrangement and using ${\overset{\cdot }{E}}_{s}^{{}^{\circ}}={\overset{\cdot }{q}}_{s}^{{}^{\circ}}/\eta^{{}^{\circ}}$ , we obtain a convenient form

(12)
$${\overset{\cdot }{S}}_{\mathrm{dev}}=\left(\frac{\eta^{*}}{\eta^{{}^{\circ}}}-1\right)\;\frac{\eta^{{}^{\circ}}}{\eta^{*}}{\overset{\cdot }{E}}_{s}^{{}^{\circ}}.$$

##### B.4.2. Emplacement effect

The emplacement effect accounts for performance of the EEU only. No behavior changes occur. The direct emplacement effect of the EEU is device energy savings and energy cost savings. The indirect emplacement effects of the EEU produce changes in the embodied energy rate and the maintenance and disposal expenditure rates. By definition, the direct emplacement effect has no rebound. However, indirect emplacement effects may cause energy rebound. Both direct and indirect emplacement effects are discussed below.

*Direct emplacement effect rebound expression* ( $Re_{\mathrm{dempl}}$ ). As shown in Table B.3, the direct rebound from the emplacement effect is $Re_{\mathrm{dempl}}\;\equiv \;0$ . This result is expected, because in the absence of embodied energy, maintenance and disposal cost, or behavioral changes, there is no takeback of energy savings at the upgraded device.

*Indirect emplacement effect rebound expression* ( $Re_{\mathrm{iempl}}$ ). Indirect emplacement rebound effects can occur at any point in the life cycle of an energy conversion device, from manufacturing and distribution to the use phase (maintenance), and finally to disposal. For simplicity, we group maintenance with disposal to form two distinct indirect emplacement rebound effects: (i) an embodied energy effect ( $Re_{\mathrm{emb}}$ ) and (ii) a maintenance and disposal effect ( $Re_{\mathrm{md}}$ ).

*Embodied energy effect rebound expression* ( $Re_{\mathrm{emb}}$ ). The first component of indirect emplacement effect rebound involves embodied energy. We define embodied energy consistent with the energy analysis literature to be the sum of all final energy consumed in the production of the energy conversion device. The EEU causes the embodied final energy of the device to change from ${\overset{\cdot }{E}}_{\mathrm{emb}}^{{}^{\circ}}$ to ${\overset{\cdot }{E}}_{\mathrm{emb}}^{*}$ .

Energy is embodied in the device within manufacturing and distribution supply chains prior to consumer acquisition of the device. For simplicity, we spread all embodied energy over the lifetime of the device, an equal amount assigned to each period.

Thus, we allocate embodied energy over the life of the original and upgraded devices ( $t_{\mathrm{life}}^{{}^{\circ}}$ and $t_{\mathrm{life}}^{*}$ , respectively) without discounting to obtain embodied energy rates, such that ${\overset{\cdot }{E}}_{\mathrm{emb}}^{{}^{\circ}}=E_{\mathrm{emb}}^{{}^{\circ}}/t_{\mathrm{life}}^{{}^{\circ}}$ and ${\overset{\cdot }{E}}_{\mathrm{emb}}^{*}=E_{\mathrm{emb}}^{*}/t_{\mathrm{life}}^{*}$ . The change in embodied final energy due to the EEU (expressed as a rate) is given by ${\overset{\cdot }{E}}_{\mathrm{emb}}^{*}-{\overset{\cdot }{E}}_{\mathrm{emb}}^{{}^{\circ}}$ . After substitution and algebraic rearrangement, the change in embodied energy rate due to the EEU can be expressed as $[(E_{\mathrm{emb}}^{*}/E_{\mathrm{emb}}^{{}^{\circ}})(t_{\mathrm{life}}^{{}^{\circ}}/t_{\mathrm{life}}^{*})-1]{\overset{\cdot }{E}}_{\mathrm{emb}}^{{}^{\circ}}$ , a term that represents energy savings taken back due to embodied energy effects. Thus, equation (3) can be employed to write embodied energy rebound as

(14)
$$Re_{\mathrm{emb}}=\frac{\left(\frac{E_{\mathrm{emb}}^{*}}{E_{\mathrm{emb}}^{{}^{\circ}}}\frac{t_{\mathrm{life}}^{{}^{\circ}}}{t_{\mathrm{life}}^{*}}-1\right){\overset{\cdot }{E}}_{\mathrm{emb}}^{{}^{\circ}}}{{\overset{\cdot }{S}}_{\mathrm{dev}}}.$$

Embodied energy rebound can be either positive or negative, depending on the sign of the term $(E_{\mathrm{emb}}^{*}/E_{\mathrm{emb}}^{{}^{\circ}})(t_{\mathrm{life}}^{{}^{\circ}}/t_{\mathrm{life}}^{*})-1$ . Rising energy efficiency can be associated with increased device complexity and more embodied energy, such that $E_{\mathrm{emb}}^{*}\;>\;E_{\mathrm{emb}}^{{}^{\circ}}$ and $Re_{\mathrm{emb}}\;>\;0$ . However, if the upgraded device has longer life than the original device ( $t_{\mathrm{life}}^{*}\;>\;t_{\mathrm{life}}^{{}^{\circ}}$ ), ${\overset{\cdot }{E}}_{\mathrm{emb}}^{*}-{\overset{\cdot }{E}}_{\mathrm{emb}}^{{}^{\circ}}$ can be negative, meaning that the upgraded device has a lower embodied energy rate than the original device.

*Operations, maintenance, and disposal effect rebound expression* ( $Re_{\mathrm{OMd}}$ ). In addition to embodied energy effects, indirect emplacement rebound can be associated with energy demanded by operations, maintenance, and disposal expenditures. We apply discounting to end-of-life disposal expenditures to form expenditure rates such that ${\overset{\cdot }{C}}_{\mathrm{OMd}}^{{}^{\circ}}={\overset{\cdot }{C}}_{\mathrm{OM}}^{{}^{\circ}}+R_{\omega }^{{}^{\circ}}{\overset{\cdot }{C}}_{d}^{{}^{\circ}}$ and ${\overset{\cdot }{C}}_{\mathrm{OMd}}^{*}={\overset{\cdot }{C}}_{\mathrm{OM}}^{*}+R_{\omega }^{*}{\overset{\cdot }{C}}_{d}^{*}$ , with ${\overset{\cdot }{C}}_{d}\;\equiv \;C_{d}/t_{\mathrm{life}}$ . (For details, see Appendix B.1.)

We assume, for simplicity, that operations, maintenance, and disposal expenditures indicate energy consumption elsewhere in the economy at its energy intensity ( $I_{E}$ ). Therefore, the change in energy consumption rate caused by a change in operations, maintenance, and disposal expenditures is given by $\Delta {\overset{\cdot }{C}}_{\mathrm{OMd}}^{*}I_{E}$ . This term is an energy takeback rate, so maintenance and disposal rebound is given by

(117)
$$Re_{\mathrm{OMd}}=\frac{\Delta {\overset{\cdot }{C}}_{\mathrm{OMd}}^{*}I_{E}}{{\overset{\cdot }{S}}_{\mathrm{dev}}},$$

as shown in Table B.3. Slight rearrangement gives

(15)
$$Re_{\mathrm{OMd}}=\frac{\left(\frac{{\overset{\cdot }{C}}_{\mathrm{OMd}}^{*}}{{\overset{\cdot }{C}}_{\mathrm{OMd}}^{{}^{\circ}}}-1\right){\overset{\cdot }{C}}_{\mathrm{OMd}}^{{}^{\circ}}I_{E}}{{\overset{\cdot }{S}}_{\mathrm{dev}}}.$$

Rebound from operations, maintenance, and disposal can be positive or negative, depending on the sign of the term ${\overset{\cdot }{C}}_{\mathrm{OMd}}^{*}/{\overset{\cdot }{C}}_{\mathrm{OMd}}^{{}^{\circ}}-1$ .

##### B.4.3. Substitution effect

This section derives expressions for substitution effect rebound. Two terms comprise substitution effect rebound, direct substitution rebound ( $Re_{\mathrm{dsub}}$ ) and indirect substitution rebound ( $Re_{\mathrm{isub}}$ ). Assuming that conditions after the emplacement effect (*) are known, both the rate of energy service consumption ( ${\hat{\overset{\cdot }{q}}}_{s}$ ) and the rate of other goods consumption ( ${\hat{\overset{\cdot }{C}}}_{g}$ ) must be determined as a result of the substitution effect (the the^point point).

The EEU’s energy efficiency increase ( $\eta^{{}^{\circ}}\;<\;\tilde{\eta }$ ) causes the price of the energy service provided by the device to fall ( $p_{s}^{{}^{\circ}}\;>\;{\tilde{p}}_{s}$ ). The substitution effect quantifies the amount by which the device user, in response, increases the consumption rate of the energy service ( ${\overset{\cdot }{q}}_{s}^{*}\;<\;{\hat{\overset{\cdot }{q}}}_{s}$ ) and decreases the consumption rate of other goods ( ${\overset{\cdot }{q}}_{g}^{*}\;>\;{\hat{\overset{\cdot }{q}}}_{g}$ ).

The increase in consumption of the energy service substitutes for consumption of other goods in the economy, subject to a utility constraint. The reduction in spending on other goods in the economy is captured by indirect substitution rebound ( $Re_{\mathrm{isub}}$ ).

We begin by deriving an expression for direct and indirect substitution effect rebound ( $Re_{\mathrm{dsub}}$ and $Re_{\mathrm{isub}}$ , respectively). Thereafter, we develop a constant price elasticity (CPE) utility model and a constant elasticity of substitution (CES) utility model for determining the post-substitution point ( ${\hat{\overset{\cdot }{q}}}_{s}$ and ${\hat{\overset{\cdot }{C}}}_{g}$ ).

*Direct substitution effect rebound expression*. Direct substitution effect rebound ( $Re_{\mathrm{dsub}}$ ) is given by

(17)
$$Re_{\mathrm{dsub}}=\frac{\Delta {\hat{\overset{\cdot }{E}}}_{s}}{{\overset{\cdot }{S}}_{\mathrm{dev}}}=\frac{{\hat{\overset{\cdot }{E}}}_{s}-{\overset{\cdot }{E}}_{s}^{*}}{{\overset{\cdot }{S}}_{\mathrm{dev}}}.$$

Substituting the typical relationship of equation (6) in the form ${\overset{\cdot }{E}}_{s}={\overset{\cdot }{q}}_{s}/\eta$ gives

(118)
$$Re_{\mathrm{dsub}}=\frac{\frac{{\hat{\overset{\cdot }{q}}}_{s}}{\hat{\eta }}-\frac{{\overset{\cdot }{q}}_{s}^{*}}{\eta^{*}}}{{\overset{\cdot }{S}}_{\mathrm{dev}}}.$$

Realizing that $\eta^{*}=\hat{\eta }$ and rearranging produces

(119)
$$Re_{\mathrm{dsub}}=\frac{\left(\frac{{\hat{\overset{\cdot }{q}}}_{s}}{{\overset{\cdot }{q}}_{s}^{{}^{\circ}}}-\frac{{\overset{\cdot }{q}}_{s}^{*}}{{\overset{\cdot }{q}}_{s}^{{}^{\circ}}}\right)\frac{{\overset{\cdot }{q}}_{s}^{{}^{\circ}}}{\eta^{*}}}{{\overset{\cdot }{S}}_{\mathrm{dev}}}.$$

Recognizing that the rate of energy service consumption ( ${\overset{\cdot }{q}}_{s}$ ) is unchanged across the emplacement effect leads to ${\overset{\cdot }{q}}_{s}^{*}/{\overset{\cdot }{q}}_{s}^{{}^{\circ}}=1$ . Furthermore, ${\dot{q}}_{s}^{{}^{\circ}}/\eta^{*}=\left({\dot{q}}_{s}^{{}^{\circ}}/\eta^{{}^{\circ}}\right)\left(\eta^{{}^{\circ}}/\eta^{*}\right)={\dot{E}}_{s}^{{}^{\circ}}\left(\eta^{{}^{\circ}}/\eta^{*}\right)$ , such that

(120)
$$Re_{\mathrm{dsub}}=\left(\frac{{\hat{\overset{\cdot }{q}}}_{s}}{{\overset{\cdot }{q}}_{s}^{{}^{\circ}}}-1\right)\frac{{\overset{\cdot }{E}}_{s}^{{}^{\circ}}\frac{\eta^{{}^{\circ}}}{\eta^{*}}}{{\overset{\cdot }{S}}_{\mathrm{dev}}}.$$

Substituting equation (12) for ${\overset{\cdot }{S}}_{\mathrm{dev}}$ and rearranging gives

(121)
Redsub=q·^sq·s°−1η*η°−1(E·s°η°η*η°η*E·s°).

Canceling terms yields

(18)
$$Re_{\mathrm{dsub}}=\frac{\frac{{\hat{\overset{\cdot }{q}}}_{s}}{{\overset{\cdot }{q}}_{s}^{{}^{\circ}}}-1}{\frac{\eta^{*}}{\eta^{{}^{\circ}}}-1}.$$

Equation (18) is the basis for developing expressions for $Re_{\mathrm{dsub}}$ under both the CPE and the CES utility models.

*Indirect substitution effect rebound expression*. Indirect substitution effect rebound ( $Re_{\mathrm{isub}}$ ) is given by

(19)
$$Re_{\mathrm{isub}}=\frac{\Delta {\hat{\overset{\cdot }{C}}}_{g}I_{E}}{{\overset{\cdot }{S}}_{\mathrm{dev}}}=\frac{\left({\hat{\overset{\cdot }{C}}}_{g}-{\overset{\cdot }{C}}_{g}^{*}\right)I_{E}}{{\overset{\cdot }{S}}_{\mathrm{dev}}}.$$

Rearranging gives

(122)
$$Re_{\mathrm{isub}}=\frac{\left(\;\frac{{\hat{\overset{\cdot }{C}}}_{g}}{{\overset{\cdot }{C}}_{g}^{{}^{\circ}}}-\frac{{\overset{\cdot }{C}}_{g}^{*}}{{\overset{\cdot }{C}}_{g}^{{}^{\circ}}}\right){\overset{\cdot }{C}}_{g}^{{}^{\circ}}I_{E}}{{\overset{\cdot }{S}}_{\mathrm{dev}}}.$$

Recognizing that expenditures on other goods are constant across the emplacement effect gives ${\overset{\cdot }{C}}_{g}^{*}/{\overset{\cdot }{C}}_{g}^{{}^{\circ}}=1$ and

(123)
$$Re_{\mathrm{isub}}=\left(\frac{{\hat{\overset{\cdot }{C}}}_{g}}{{\overset{\cdot }{C}}_{g}^{{}^{\circ}}}-1\right)\frac{{\overset{\cdot }{C}}_{g}^{{}^{\circ}}I_{E}}{{\overset{\cdot }{S}}_{\mathrm{dev}}}.$$

Substituting equation ( 12 ) for ${\overset{\cdot }{S}}_{\mathrm{dev}}$ and rearranging gives

(20)
$$Re_{\mathrm{isub}}=\frac{\frac{{\hat{\overset{\cdot }{C}}}_{g}}{{\overset{\cdot }{C}}_{g}^{{}^{\circ}}}-1}{\frac{\eta^{*}}{\eta^{{}^{\circ}}}-1}\;\frac{\eta^{*}}{\eta^{{}^{\circ}}}\;\frac{{\overset{\cdot }{C}}_{g}^{{}^{\circ}}I_{E}}{{\overset{\cdot }{E}}_{s}^{{}^{\circ}}}.$$

Equation (20) is the basis for developing expressions for $Re_{\mathrm{isub}}$ under both the CPE and the CES utility models.

Determining the post-substitution effect conditions requires reference to a consumer utility model. We first show the CPE utility model, often used in the literature. Second, we use a constant elasticity of substitution (CES) utility model. The CES utility model is used for nearly all calculations and graphs in this paper.

*Constant price elasticity (CPE) utility model*. In the literature, a constant price elasticity (CPE) utility model has been used to determine conditions after the substitution effect (∧) (Borenstein 2015, 17: footnote 43). However, the CPE model does not produce precisely utility-preserving preferences, thus it cannot calculate the actual substitution effect. We discuss the CPE utility model here for comparison purposes only.

Borenstein‘s CPE utility model uses the reduced form relationship between energy service price ( $p_{s}$ ) and energy service consumption rate ( ${\overset{\cdot }{q}}_{s}$ ), namely the observed, uncompensated own price elasticity of energy service demand ( $\varepsilon_{{\overset{\cdot }{q}}_{s},p_{s}}$ ), such that

(124)
$$\frac{{\hat{\overset{\cdot }{q}}}_{s}}{{\overset{\cdot }{q}}_{s}^{*}}={\left(\frac{p_{s}^{*}}{p_{s}^{{}^{\circ}}}\right)}^{\varepsilon_{{\overset{\cdot }{q}}_{s},p_{s}}}.$$

Note that the uncompensated own price elasticity of energy service demand ( $\varepsilon_{{\overset{\cdot }{q}}_{s},p_{s}}$ ) is assumed constant in the CPE utility model. A negative value for the uncompensated own price elasticity of energy service demand is expected ( $\varepsilon_{{\overset{\cdot }{q}}_{s},p_{s}}\;<\;0$ ), such that when the energy service price decreases ( $p_{s}^{{}^{\circ}}\;>\;p_{s}^{*}$ ), the rate of energy service consumption increases ( ${\overset{\cdot }{q}}_{s}^{*}\;<\;{\hat{\overset{\cdot }{q}}}_{s}$ ).

Substituting equation (7) in the form $p_{s}^{{}^{\circ}}=p_{E}^{{}^{\circ}}/\eta^{{}^{\circ}}$ and $p_{s}^{*}=p_{E}^{{}^{\circ}}/\eta^{*}$ and noting that ${\overset{\cdot }{q}}_{s}^{{}^{\circ}}={\overset{\cdot }{q}}_{s}^{*}$ gives

(125)
$$\frac{{\hat{\overset{\cdot }{q}}}_{s}}{{\overset{\cdot }{q}}_{s}^{{}^{\circ}}}={\left(\frac{\eta^{*}}{\eta^{{}^{\circ}}}\right)}^{-\varepsilon_{{\overset{\cdot }{q}}_{s},p_{s}}}.$$

Again, note that the compensated own price elasticity of energy service demand is negative ( $\varepsilon_{{\overset{\cdot }{q}}_{s},p_{s}}\;<\;0$ ), so that as energy service efficiency increases $\left(\eta^{{}^{\circ}}\;<\;\eta^{*}\right)$ , the energy service consumption rate increases ( ${\overset{\cdot }{q}}_{s}^{{}^{\circ}}={\overset{\cdot }{q}}_{s}^{*}\;<\;{\hat{\overset{\cdot }{q}}}_{s}$ ) as well.

Substituting equations (125) into (18) yields the CPE model’s expression for direct substitution rebound

(126)
$$Re_{\mathrm{dsub}}=\frac{{\left(\frac{\eta^{*}}{\eta^{{}^{\circ}}}\right)}^{-\varepsilon_{{\overset{\cdot }{q}}_{s},p_{s}}}-1}{\frac{\eta^{*}}{\eta^{{}^{\circ}}}-1},$$

such that, for example, $\varepsilon_{{\overset{\cdot }{q}}_{s},p_{s}}=-0.2$ and $\eta^{*}/\eta^{{}^{\circ}}=2$ yields $Re_{\mathrm{dsub}}=0.15$ .

As long as $\varepsilon_{{\overset{\cdot }{q}}_{s},p_{s}}\in \left(-1,0\right)$ , the CPE utility model indicates that direct substitution rebound will be below 1. At $\varepsilon_{{\overset{\cdot }{q}}_{s},p_{s}}=1$ , the effect would be the same as the Cobb-Douglas utility model (see footnote 16) and the sum of substitution and income rebound effects would be exactly 100%.

To quantify the substitution effect on other purchases in the CPE utility model, expenditure on other goods is reduced by the same dollar amount as expenditure on the energy service increased due to the direct substitution effect: expenditure is held constant. Thus,

(127)
$$\Delta {\hat{\overset{\cdot }{C}}}_{g}=-\Delta {\hat{\overset{\cdot }{C}}}_{s}.$$

The advantage of this approach is that no cross price elasticity is needed. The disadvantage is that it does not adhere to the definition of the substitution effect, which assumes that utility, not expenditure, is held constant.

Solving for ${\hat{\overset{\cdot }{C}}}_{g}/{\overset{\cdot }{C}}_{g}^{*}$ , substituting an expression for the change in expenditure on the energy service ( $\Delta {\hat{\overset{\cdot }{C}}}_{s}$ ), namely

(128)
$$\Delta {\hat{\overset{\cdot }{C}}}_{s}=\frac{p_{E}\left({\hat{\overset{\cdot }{q}}}_{s}-{\overset{\cdot }{q}}_{s}^{*}\right)}{\eta^{*}},$$

and substituting equation (125) gives

(129)
$$\frac{{\hat{\overset{\cdot }{C}}}_{g}}{{\overset{\cdot }{C}}_{g}^{*}}=1-\frac{p_{E}{\overset{\cdot }{q}}_{s}^{*}}{\eta^{*}{\overset{\cdot }{C}}_{g}^{*}}\left[{\left(\frac{\eta^{*}}{\eta^{{}^{\circ}}}\right)}^{-\varepsilon_{{\overset{\cdot }{q}}_{s},p_{s}}}-1\right].$$

Substituting equations (129) into (20) gives

(130)
$$Re_{\mathrm{isub}}=-\frac{\frac{p_{E}{\overset{\cdot }{q}}_{s}^{*}}{\eta^{*}{\overset{\cdot }{C}}_{g}^{*}}\left[{\left(\frac{\eta^{*}}{\eta^{{}^{\circ}}}\right)}^{-\varepsilon_{{\overset{\cdot }{q}}_{s},p_{s}}}-1\right]}{\frac{\eta^{*}}{\eta^{{}^{\circ}}}-1}\frac{\eta^{*}}{\eta^{{}^{\circ}}}\frac{{\overset{\cdot }{C}}_{g}^{{}^{\circ}}I_{E}}{{\overset{\cdot }{E}}_{s}^{{}^{\circ}}}.$$

Rearranging and substituting equation (126) gives the expression for indirect substitution rebound under the CPE utility model.

(131)
$$Re_{\mathrm{isub}}=-\frac{{\overset{\cdot }{q}}_{s}^{*}{\overset{\cdot }{C}}_{g}^{{}^{\circ}}p_{E}I_{E}}{\eta^{{}^{\circ}}{\overset{\cdot }{C}}_{g}^{*}{\overset{\cdot }{E}}_{s}^{{}^{\circ}}}Re_{\mathrm{dsub}}$$

Because (i) the compensated cross price elasticity of other goods consumption is positive ( $\varepsilon_{{\overset{\cdot }{q}}_{g},p_{s},c}\;>\;0$ ), that is, we exclude Giffen goods (Spiegel 1994) whose consumption declines as their price declines and (ii) the energy service efficiency ratio is greater than 1 ( $\eta^{{}^{\circ}}\;<\;\tilde{\eta }$ ), direct substitution rebound will be positive always ( $Re_{\mathrm{dsub}}\;>\;0$ ) and indirect substitution rebound will be negative always ( $Re_{\mathrm{isub}}\;<\;0$ ), as expected, under the CPE utility model. Negative rebound indicates that indirect substitution effects reduce the energy takeback rate by direct substitution effects.

*CES utility model*. The CPE utility model assumes that the compensated own price elasticity of energy service demand ( $\varepsilon_{{\overset{\cdot }{q}}_{s},p_{s},c}$ ) is constant along an indifference curve, an assumption that holds only for infinitesimally small energy service price changes ( $\Delta p_{s}^{*}\;\equiv \;p_{s}^{*}-p_{s}^{{}^{\circ}}\approx 0$ ). The CPE utility model provides reasonable approximations for a 1% to 2% change in energy efficiency. However, in the case of an energy efficiency upgrade (EEU), the energy service price change is neither infinitesimal nor confined to single-digit percentages. Rather, $\Delta p_{s}^{*}$ is finite and may be very large in percentage terms.

To determine the new consumption bundle after the substitution effect ( ${\hat{\overset{\cdot }{q}}}_{s}$ and ${\hat{\overset{\cdot }{C}}}_{g}$ ) and, ultimately, to quantify the direct and indirect substitution rebound effects ( $Re_{\mathrm{dsub}}$ and $Re_{\mathrm{isub}}$ ) exactly, we remove the restriction that energy service price elasticity ( $\varepsilon_{{\overset{\cdot }{q}}_{s},p_{s}}$ ) must be constant along an indifference curve (as in the CPE utility model). Instead, we require constancy of only the elasticity of substitution ( σ ) between the consumption rate of the energy service ( ${\overset{\cdot }{q}}_{s}$ ) and the expenditure rate for other goods ( ${\overset{\cdot }{C}}_{g}$ ) across the substitution effect. Thus, we employ a CES utility model in our framework. Figures 4 and 7 in Heun et al. (2025) (especially segments *−−c and c−−∧ ) illustrates features of the CES utility model for determining the new consumption bundle.

Two equations are helpful for this analysis. First, the slope at any point on indifference curve (the $i^{{}^{\circ}}--\;i^{{}^{\circ}}$ curve in Figures 4 and 7 of Heun et al. (2025)) is given by equation (160) with $\overset{\cdot }{u}/{\overset{\cdot }{u}}^{{}^{\circ}}=1$ and the share parameter ( a ) replaced by $f_{{\overset{\cdot }{C}}_{s}}^{{}^{\circ}}$ , as discussed in Appendix C.

(132)
$$\begin{array}{c} \frac{\partial \left(\frac{{\overset{\cdot }{C}}_{g}}{{\overset{\cdot }{C}}_{g}^{{}^{\circ}}}\right)}{\partial \left(\frac{{\overset{\cdot }{q}}_{s}}{{\overset{\cdot }{q}}_{s}^{{}^{\circ}}}\right)}=-\frac{f_{{\overset{\cdot }{C}}_{s}}^{{}^{\circ}}}{1-f_{{\overset{\cdot }{C}}_{s}}^{{}^{\circ}}}{\left(\frac{{\overset{\cdot }{q}}_{s}}{{\overset{\cdot }{q}}_{s}^{{}^{\circ}}}\right)}^{\left(\rho -1\right)}{\left[\left(\frac{1}{1-f_{{\overset{\cdot }{C}}_{s}}^{{}^{\circ}}}\right){\left(\frac{\overset{\cdot }{q}}{{\overset{\cdot }{q}}_{s}^{{}^{\circ}}}\right)}^{\rho }\right]}^{\left(1-\rho \right)/\rho }. \end{array}$$

Second, the equation of the pre-substitution-effect expenditure line (*−−* in Figures 4 and 7 of Heun et al. (2025)) is

(133)
$$\frac{{\overset{\cdot }{C}}_{g}}{{\overset{\cdot }{C}}_{g}^{{}^{\circ}}}=-\frac{p_{s}^{*}{\overset{\cdot }{q}}_{s}^{{}^{\circ}}}{{\overset{\cdot }{C}}_{g}^{{}^{\circ}}}\left(\frac{{\overset{\cdot }{q}}_{s}}{{\overset{\cdot }{q}}_{s}^{{}^{\circ}}}\right)+\frac{1}{{\overset{\cdot }{C}}_{g}^{{}^{\circ}}}\left(\overset{\cdot }{M}-\tau_{\alpha }^{{}^{\circ}}{\overset{\cdot }{C}}_{\mathrm{cap}}^{{}^{\circ}}-{\overset{\cdot }{C}}_{\mathrm{OMd}}^{{}^{\circ}}-\overset{\cdot }{G}\right).$$

To find the rate of energy service consumption after the substitution effect ( ${\hat{\overset{\cdot }{q}}}_{s}$ ), we set the slope of the expenditure line (equation (133) and line *−−* in Figures 4 and 7 of Heun et al. (2025)) equal to the slope of the indifference curve ( $i^{{}^{\circ}}--i^{{}^{\circ}}$ in Figures 4 and 7 of Heun et al. (2025)) at the original utility rate of $\overset{\cdot }{u}/{\overset{\cdot }{u}}^{{}^{\circ}}=1$ (equation (132)).

(134)
$$-\frac{p_{s}^{*}{\overset{\cdot }{q}}_{s}^{{}^{\circ}}}{{\overset{\cdot }{C}}_{g}^{{}^{\circ}}}=-\frac{f_{{\overset{\cdot }{C}}_{s}}^{{}^{\circ}}}{1-f_{{\overset{\cdot }{C}}_{s}}^{{}^{\circ}}}{\left(\frac{{\overset{\cdot }{q}}_{s}}{{\overset{\cdot }{q}}_{s}^{{}^{\circ}}}\right)}^{\left(\rho -1\right)}{\left[\left(\frac{1}{1-f_{{\overset{\cdot }{C}}_{s}}^{{}^{\circ}}}\right)-\left(\frac{f_{{\overset{\cdot }{C}}_{s}}^{{}^{\circ}}}{1-f_{{\overset{\cdot }{C}}_{s}}^{{}^{\circ}}}\right){\left(\frac{\overset{\cdot }{q}}{{\overset{\cdot }{q}}_{s}^{{}^{\circ}}}\right)}^{\rho }\right]}^{\left(1-\rho \right)/\rho }$$

Solving for ${\overset{\cdot }{q}}_{s}/{\overset{\cdot }{q}}_{s}^{{}^{\circ}}$ gives ${\hat{\overset{\cdot }{q}}}_{s}/{\overset{\cdot }{q}}_{s}^{{}^{\circ}}$ as

(21)
$$\frac{{\hat{\overset{\cdot }{q}}}_{s}}{{\overset{\cdot }{q}}_{s}^{{}^{\circ}}}={\left\{f_{{\overset{\cdot }{C}}_{s}}^{{}^{\circ}}+\left(1-f_{{\overset{\cdot }{C}}_{s}}^{{}^{\circ}}\right){\left[\left(\frac{1-f_{{\overset{\cdot }{C}}_{s}}^{{}^{\circ}}}{f_{{\overset{\cdot }{C}}_{s}}^{{}^{\circ}}}\right)\frac{p_{s}^{*}{\overset{\cdot }{q}}_{s}^{{}^{\circ}}}{{\overset{\cdot }{C}}_{g}^{{}^{\circ}}}\right]}^{\rho /\left(1-\rho \right)}\right\}}^{-1/\rho }.$$

Equation (21) can be substituted directly into equation (18) to obtain an estimate for direct substitution rebound ( $Re_{\mathrm{dsub}}$ ) via the CES utility model.

(23)
$$Re_{\mathrm{dsub}}=\frac{{\left\{f_{{\overset{\cdot }{C}}_{s}}^{{}^{\circ}}+\left(1-f_{{\overset{\cdot }{C}}_{s}}^{{}^{\circ}}\right){\left[\left(\frac{1-f_{{\overset{\cdot }{C}}_{s}}^{{}^{\circ}}}{f_{{\overset{\cdot }{C}}_{s}}^{{}^{\circ}}}\right)\frac{p_{s}^{*}{\overset{\cdot }{q}}_{s}^{{}^{\circ}}}{{\overset{\cdot }{C}}_{g}^{{}^{\circ}}}\right]}^{\rho /\left(1-\rho \right)}\right\}}^{-1/\rho }-1}{\frac{\hat{\eta }}{\eta^{{}^{\circ}}}-1}$$

The rate of other goods consumption after the substitution effect ( ${\hat{\overset{\cdot }{C}}}_{g}$ ) can be found by substituting equation (21) and $\overset{\cdot }{u}/{\overset{\cdot }{u}}^{{}^{\circ}}=1$ into the functional form of the CES utility model (equation (159)) to obtain

(135)
$$\frac{{\hat{\overset{\cdot }{C}}}_{g}}{{\overset{\cdot }{C}}_{g}^{{}^{\circ}}}={\left(\left(\frac{1}{1-f_{{\overset{\cdot }{C}}_{s}}^{{}^{\circ}}}\right)-\left(\frac{f_{{\overset{\cdot }{C}}_{s}}^{{}^{\circ}}}{1-f_{{\overset{\cdot }{C}}_{s}}^{{}^{\circ}}}\right){\left\{f_{{\overset{\cdot }{C}}_{s}}^{{}^{\circ}}+\left(1-f_{{\overset{\cdot }{C}}_{s}}^{{}^{\circ}}\right){\left[\left(\frac{1-f_{{\overset{\cdot }{C}}_{s}}^{{}^{\circ}}}{f_{{\overset{\cdot }{C}}_{s}}^{{}^{\circ}}}\right)\frac{p_{s}^{*}{\overset{\cdot }{q}}_{s}^{{}^{\circ}}}{{\overset{\cdot }{C}}_{g}^{{}^{\circ}}}\right]}^{\frac{\rho }{1-\rho }}\right\}}^{-1}\right)}^{1/\rho }.$$

Simplifying gives

(22)
$$\frac{{\hat{\overset{\cdot }{C}}}_{g}}{{\overset{\cdot }{C}}_{g}^{{}^{\circ}}}={\left(1+f_{{\overset{\cdot }{C}}_{s}}^{{}^{\circ}}\left\{{\left[\left(\frac{1-f_{{\overset{\cdot }{C}}_{s}}^{{}^{\circ}}}{f_{{\overset{\cdot }{C}}_{s}}^{{}^{\circ}}}\right)\frac{p_{s}^{*}{\overset{\cdot }{q}}_{s}^{{}^{\circ}}}{{\overset{\cdot }{C}}_{g}^{{}^{\circ}}}\right]}^{\rho /\left(\rho -1\right)}-1\right\}\right)}^{-1/\rho }.$$

Equation (22) can be substituted into equation (20) to obtain an expression for indirect substitution rebound ( $Re_{\mathrm{isub}}$ ) via the CES utility model.

(24)
$$Re_{\mathrm{isub}}=\frac{{\left(1+f_{{\overset{\cdot }{C}}_{s}}^{{}^{\circ}}\left\{{\left[\left(\frac{1-f_{{\overset{\cdot }{C}}_{s}}^{{}^{\circ}}}{f_{{\overset{\cdot }{C}}_{s}}^{{}^{\circ}}}\right)\frac{p_{s}^{*}{\overset{\cdot }{q}}_{s}^{{}^{\circ}}}{{\overset{\cdot }{C}}_{g}^{{}^{\circ}}}\right]}^{\rho /\left(\rho -1\right)}-1\right\}\right)}^{-1/\rho }-1}{\frac{\hat{\eta }}{\eta^{{}^{\circ}}}-1}\frac{\hat{\eta }}{\eta^{{}^{\circ}}}\;\frac{{\overset{\cdot }{C}}_{g}^{{}^{\circ}}I_{E}}{{\overset{\cdot }{E}}_{s}^{{}^{\circ}}}$$

##### B.4.4. Income effect

Rebound from the income effect rebound quantifies the rate of additional energy demand that arises because the user of the energy conversion device spends net savings from the EEU. The income rate of the device user is $\overset{\cdot }{M}$ , which remains unchanged across the rebound effects. Freed cash from the EEU is given by equation (90) as $\overset{\cdot }{G}=p_{E}{\overset{\cdot }{S}}_{\mathrm{dev}}$ . In combination, the emplacement effect and the substitution effect leave the device user with *net* savings ( $\hat{\overset{\cdot }{N}}$ ) from the EEU, as shown in equation (100). Derivations of expressions for freed cash from the emplacement effect ( $\overset{\cdot }{G}$ ) and net savings after the substitution effect ( $\hat{\overset{\cdot }{N}}$ ) are presented in Tables B.3 and B.4.

In this framework, all net savings ( $\hat{\overset{\cdot }{N}}$ ) are spent on either (i) additional energy service ( ${\hat{\overset{\cdot }{q}}}_{s}\;<\;{\bar{\overset{\cdot }{q}}}_{s}$ ) or (ii) additional other goods ( ${\hat{\overset{\cdot }{q}}}_{g}\;<\;{\bar{\overset{\cdot }{q}}}_{g}$ ). The income elasticity of energy service demand and the income elasticity of other goods demand ( $\varepsilon_{{\overset{\cdot }{q}}_{s},\overset{\cdot }{M}}$ and $\varepsilon_{{\overset{\cdot }{q}}_{g},\overset{\cdot }{M}}$ , respectively) quantify the income preferences of the device user according to the following expressions:

(25)
q·¯sq·^s=(1+N·^M·^')εq·s,M·

and

(29)
q·¯gq·^g=(1+N·^M·^')εq·g,M·,

where effective income ( M·^' ) is

(26)
M·^'≡M·−τα*C·cap*−C·OMd*−N·^.

Homotheticity means that $\varepsilon_{{\overset{\cdot }{q}}_{s},\overset{\cdot }{M}}=1$ and $\varepsilon_{{\overset{\cdot }{q}}_{g},\overset{\cdot }{M}}=1$ .

The budget constraint across the income effect (equation (108)) ensures that all net savings available after the substitution effect ( $\hat{\overset{\cdot }{N}}$ ) is re-spent across the income effect, such that $\bar{\overset{\cdot }{N}}=0$ . Appendix D proves that the income preference equations (equations (25) and (29)) satisfy the budget constraint (equation (108)).

The purpose of this section is derivation of expressions for (i) direct income rebound ( $Re_{\mathrm{dinc}}$ ) arising from increased consumption of the energy service ( ${\hat{\dot{q}}}_{s}<\;{\bar{\dot{q}}}_{s}$ ) and (ii) indirect income rebound ( $Re_{\mathrm{iinc}}$ ) arising from increased consumption of other goods ( ${\hat{\overset{\cdot }{q}}}_{g}\;<\;{\bar{\overset{\cdot }{q}}}_{g}$ ).

But first, we derive an expression for device energy consumption rate prior to the income effect ( ${\hat{\overset{\cdot }{E}}}_{s}$ ). This expression will be helpful later.

*Derivation of expression for* ${\hat{\overset{\cdot }{E}}}_{s}$ . An expression for ${\hat{\overset{\cdot }{E}}}_{s}$ . that will be helpful later begins with

(136)
$${\hat{\overset{\cdot }{E}}}_{s}=\left(\frac{{\hat{\overset{\cdot }{E}}}_{s}}{{\overset{\cdot }{E}}_{s}^{*}}\right)\left(\frac{{\overset{\cdot }{E}}_{s}^{*}}{{\overset{\cdot }{E}}_{s}^{{}^{\circ}}}\right){\overset{\cdot }{E}}_{s}^{{}^{\circ}}.$$

Substituting equation (6) and noting efficiency ( η ) equalities from Table B.1 gives

(137)
E·^s=(q·^s/η^q·s*/η*)(q·s*/η*q·s°/η°)E·s°.

Canceling terms yields

(138)
E·^s=(q·^sq·s*)(q·s*q·s°)(η°η*)E·s°.

Noting energy service consumption rate equalities from Table B.1 ( ${\overset{\cdot }{q}}_{s}^{*}={\overset{\cdot }{q}}_{s}^{{}^{\circ}}$ ) gives

(139)
$${\hat{\overset{\cdot }{E}}}_{s}=\frac{{\hat{\overset{\cdot }{q}}}_{s}}{{\overset{\cdot }{q}}_{s}^{*}}\frac{\eta^{{}^{\circ}}}{\eta^{*}}{\overset{\cdot }{E}}_{s}^{{}^{\circ}}.$$

The next step is to develop an expression for $Re_{\mathrm{dinc}}$ using the income preference for energy service consumption.

*Derivation of expression for* $Re_{\mathrm{dinc}}.$ As shown in Table B.5, direct income rebound is defined as

(27)
$$Re_{\mathrm{dinc}}\;\equiv \;\frac{\Delta {\bar{\overset{\cdot }{E}}}_{s}}{{\overset{\cdot }{S}}_{\mathrm{dev}}}.$$

Expanding the difference and rearranging gives

(140)
$$Re_{\mathrm{dinc}}=\frac{{\bar{\overset{\cdot }{E}}}_{s}-{\hat{\overset{\cdot }{E}}}_{s}}{{\overset{\cdot }{S}}_{\mathrm{dev}}}\;,$$

and

(141)
$$Re_{\mathrm{dinc}}=\frac{\left(\frac{{\bar{\overset{\cdot }{E}}}_{s}}{{\hat{\overset{\cdot }{E}}}_{s}}-1\right){\hat{\overset{\cdot }{E}}}_{s}}{{\overset{\cdot }{S}}_{\mathrm{dev}}}.$$

Substituting equation (6) as ${\bar{\overset{\cdot }{E}}}_{s}={\bar{\overset{\cdot }{q}}}_{s}/\bar{\eta }$ and ${\hat{\overset{\cdot }{E}}}_{s}={\hat{\overset{\cdot }{q}}}_{s}/\hat{\eta }$ gives

(142)
Redinc=(q·¯s/η¯q·^s/η^−1)E·^sS·dev.

Eliminating terms and substituting equation (12) for ${\overset{\cdot }{S}}_{\mathrm{dev}}$ and equation (25) for ${\bar{\overset{\cdot }{q}}}_{s}/{\hat{\overset{\cdot }{q}}}_{s}$ gives

(143)
Redinc=[(1+N·^M·^')εq·s,M·−1]E·^s(η*η°−1)η°η*E·s°.

Substituting equation (139) for ${\hat{\overset{\cdot }{E}}}_{s}$ gives

(144)
Redinc=[(1+N·^M·^')εq·s,M·−1]q·^sq·s*η°η*E·s°(η*η°−1)η°η*E·s°.

Eliminating terms, recognizing that ${\overset{\cdot }{q}}_{s}^{{}^{\circ}}={\overset{\cdot }{q}}_{s}^{*}$ , and substituting equation (21), which assumes the CES utility model, gives

(28)
Redinc=(1+N·^M·^')εq·s,M·−1η*η°−1{fC·s°+(1−fC·s°)[(1−fC·s°fC·s°)ps*q·s°C·g°]ρ/(1−ρ)}−1/ρ.

If there is no net savings ( $\hat{\overset{\cdot }{N}}=0$ ), direct income effect rebound is zero ( $Re_{\mathrm{dinc}}=0$ ), as expected.

The next step is to develop an expression for $Re_{\mathrm{iinc}}$ using the income preference for other goods consumption.

*Derivation of expression for* $Re_{\mathrm{iinc}}$ . As shown in Table B.5, indirect income rebound is defined as

(31)
$$Re_{\mathrm{iinc}}\;\equiv \;\frac{\Delta {\bar{\overset{\cdot }{C}}}_{g}I_{E}}{{\overset{\cdot }{S}}_{\mathrm{dev}}}.$$

Expanding the difference and rearranging gives

(145)
$$Re_{\mathrm{iinc}}=\frac{\left({\bar{\overset{\cdot }{C}}}_{g}-{\hat{\overset{\cdot }{C}}}_{g}\right)I_{E}}{{\overset{\cdot }{S}}_{\mathrm{dev}}}\;,$$

and

(146)
$$Re_{\mathrm{iinc}}=\frac{\left(\frac{{\bar{\overset{\cdot }{C}}}_{g}}{{\hat{\overset{\cdot }{C}}}_{g}}-1\right){\hat{\overset{\cdot }{C}}}_{g}I_{E}}{{\overset{\cdot }{S}}_{\mathrm{dev}}}.$$

Substituting ${\bar{\overset{\cdot }{C}}}_{g}=p_{g}{\bar{\overset{\cdot }{q}}}_{g}$ and ${\hat{\overset{\cdot }{C}}}_{g}=p_{g}{\hat{\overset{\cdot }{q}}}_{g}$ and cancelling terms gives

(147)
$$Re_{\mathrm{iinc}}=\frac{\left(\frac{{\bar{\overset{\cdot }{q}}}_{g}}{{\hat{\overset{\cdot }{q}}}_{g}}-1\right){\hat{\overset{\cdot }{C}}}_{g}I_{E}}{{\overset{\cdot }{S}}_{\mathrm{dev}}}.$$

Substituting the income preference equation for other goods consumption (equation (29) for ${\bar{\overset{\cdot }{q}}}_{g}/{\hat{\overset{\cdot }{q}}}_{g}$ and equation (12) for ${\overset{\cdot }{S}}_{\mathrm{dev}}$ yields

(148)
Reiinc=[(1+N·^M·^')εq·g,M·−1]C·^gIE(η*η°−1)η°η*E·s°.

Substituting $\left({\hat{\overset{\cdot }{C}}}_{g}/{\overset{\cdot }{C}}_{g}^{{}^{\circ}}\right){\overset{\cdot }{C}}_{g}^{{}^{\circ}}$ for ${\hat{\overset{\cdot }{C}}}_{g}$ , recognizing that ${\overset{\cdot }{C}}_{g}^{*}={\overset{\cdot }{C}}_{g}^{{}^{\circ}}$ , and simplifying gives

(149)
Reiinc=(1+N·^M·^')εq·g,M·−1η*η°−1(η*η°)C·g°IEE·s°(C·^gC·g°).

Substituting equation (22) for ${\hat{\overset{\cdot }{C}}}_{g}/{\overset{\cdot }{C}}_{g}^{{}^{\circ}}$ , thereby assuming the CES utility model, gives the final form of the indirect income rebound expression:

(32)
Reiinc=(1+N·^M·^')εq·g,M·−1η*η°−1(η*η°)C·g°IEE·s°×(1+fC·s°{[(1−fC·s°fC·s°)ps*q·s°C·g°]ρ/(ρ−1)−1})−1/ρ.

If there is no net savings ( $\hat{\overset{\cdot }{N}}=0$ ), indirect income effect rebound is zero ( $Re_{\mathrm{iinc}}=0$ ), as expected.

*Income effect rebound under the CPE utility model*. Following Borenstein (2015), under the CPE utility model all freed cash is spent on other goods, as in the fully satiated case discussed in Section 2.5.3. However, because the substitution effect under the CPE utility model does not alter freed cash, the income effect involves the product of the energy intensity of the economy ( $I_{E}$ ) and ${\overset{\cdot }{N}}^{*}$ (instead of $\hat{\overset{\cdot }{N}}$ ).

##### B.4.5. Macro effect

Macro rebound ( $Re_{\mathrm{macro}}$ ) is given by equation (33). Substituting equation (89) for net savings ( ${\overset{\cdot }{N}}^{*}$ ) gives

(150)
$$Re_{\mathrm{macro}}=\frac{k\left(p_{E}{\overset{\cdot }{S}}_{\mathrm{dev}}-\Delta {\left(\tau_{\alpha }{\overset{\cdot }{C}}_{\mathrm{cap}}\right)}^{*}-\Delta {\overset{\cdot }{C}}_{\mathrm{OMd}}^{*}\right)I_{E}}{{\overset{\cdot }{S}}_{\mathrm{dev}}}.$$

Separating terms gives

(151)
$$Re_{\mathrm{macro}}=\frac{kp_{E}/{\overset{\cdot }{S}}_{\mathrm{dev}}I_{E}}{/{\overset{\cdot }{S}}_{\mathrm{dev}}}-\frac{k\Delta {\left(\tau_{\alpha }{\overset{\cdot }{C}}_{\mathrm{cap}}\right)}^{*}I_{E}}{{\overset{\cdot }{S}}_{\mathrm{dev}}}-\frac{k\Delta {\overset{\cdot }{C}}_{\mathrm{OMd}}^{*}I_{E}}{{\overset{\cdot }{S}}_{\mathrm{dev}}}.$$

Canceling terms, substituting equation (117) to obtain $Re_{\mathrm{OMd}}$ , and defining $Re_{\mathrm{cap}}$ as

(152)
$$Re_{\mathrm{cap}}\;\equiv \;\frac{\Delta {\left(\tau_{\alpha }{\overset{\cdot }{C}}_{\mathrm{cap}}\right)}^{*}I_{E}}{{\overset{\cdot }{S}}_{\mathrm{dev}}}$$

gives

(34)
$$Re_{\mathrm{macro}}=k\left(p_{E}I_{E}-Re_{\mathrm{cap}}-Re_{\mathrm{OMd}}\right).$$

##### B.4.6. Rebound sum

The sum of the four rebound effects is

(153)
$$Re_{\mathrm{tot}}=Re_{\mathrm{empl}}+Re_{\mathrm{sub}}+Re_{\mathrm{inc}}+Re_{\mathrm{macro}}.$$

Substituting equations (85), (97), and (106) gives

(154)
$$\begin{array}{cc} Re_{\mathrm{tot}}=Re_{\mathrm{emb}}+Re_{\mathrm{OMd}} & \mathrm{emplacement}\;\mathrm{effect}\\ \;\;\;\;\;\;\;\;\;\;+Re_{\mathrm{dsub}}+Re_{\mathrm{isub}} & \mathrm{substitution}\;\mathrm{effect}\\ \;\;\;\;\;\;\;\;\;\;+Re_{\mathrm{dinc}}+Re_{\mathrm{iinc}} & \mathrm{income}\;\mathrm{effect}\\ \;\;\;\;\;\;\;\;\;\;+Re_{\mathrm{macro}} & \mathrm{macro}\;\mathrm{effect} \end{array}$$

Macro effect rebound ( $Re_{\mathrm{macro}}$ , equation (34)) can be expressed in terms of other rebound effects. Substituting equation (34) gives

(155)
$$\begin{array}{cc} Re_{\mathrm{tot}}=Re_{\mathrm{emb}}+Re_{\mathrm{OMd}} & \mathrm{emplacement}\;\mathrm{effect}\\ \;\;\;\;\;\;\;\;\;\;+Re_{\mathrm{dsub}}+Re_{\mathrm{isub}} & \mathrm{substitution}\;\mathrm{effect}\\ \;\;\;\;\;\;\;\;\;\;+Re_{\mathrm{dinc}}+Re_{\mathrm{iinc}} & \mathrm{income}\;\mathrm{effect}\\ \;\;\;\;\;\;\;\;\;\;+kp_{E}I_{E}-\mathrm{kR}e_{\mathrm{cap}}-\mathrm{kR}e_{\mathrm{OMd}}\;. & \mathrm{macro}\;\mathrm{effect} \end{array}$$

Rearranging distributes macro effect terms to emplacement and substitution effect terms. This last rearrangement gives the final expression for total rebound.

(35)
$$\begin{array}{c} Re_{\mathrm{tot}}=Re_{\mathrm{emb}}+k\left(p_{E}I_{E}-Re_{\mathrm{cap}}\right)+\left(1-k\right)Re_{\mathrm{OMd}}\\ \;\;\;\;\;\;\;\;\;\;+\;Re_{\mathrm{dsub}}+Re_{\mathrm{isub}}+Re_{\mathrm{dinc}}+Re_{\mathrm{iinc}} \end{array}$$

Equation (35) shows that determining seven rebound values,

- *Re*_*emb*_ (equation (14)),
- *Re*_*cap*_ (equation (152)),
- *Re*_*OMd*_ (equation (15)),
- *Re*_*dsub*_ (equation (23)),
- *Re*_*isub*_ (equation (24)),
- *Re*_*dinc*_ (equation (28)), and
- *Re*_*iinc*_ (equation (32)),

is sufficient to calculate total rebound, provided that the macro factor ( k ), the price of energy ( $p_{E}$ ), and the energy intensity of the economy ( $I_{E}$ ) are known.

### C. Utility Models and Elasticities

As discussed in Section 2.5.2 and Appendix B.4.3, the substitution effect requires a model for device user behavior. Behavior is typically represented by a model of utility that is maximized with arguments of consuming the energy service ( ${\overset{\cdot }{q}}_{s}$ ) and other goods and services ( ${\overset{\cdot }{q}}_{g}$ ) and subject to income and price constraints. In this appendix, we describe two utility models. The first utility model is a constant price elasticity (CPE) utility model, which allows an easy calculation of price-demand relationships as Appendix B.4.3 illustrates. It gives a good approximation of the behavioral response for very small changes in energy efficiency and energy service price, such that $\Delta \eta^{*}\approx 0$ and $\Delta p_{s}^{*}\approx 0$ . The CPE utility model is discussed for continuity with the literature only (see, e.g., Borenstein 2015, 17: footnote 43).

We note that larger and non-marginal efficiency gains cause greater rebound (measured in joules) than small and marginal efficiency gains. Thus, any rebound analysis framework needs to accommodate large, non-marginal efficiency changes. Since price elasticities are point-measures in analytical utility models, a version of the framework amenable to empirical applications should account for the changing price elasticity along an indifference curve.^25^ The second utility model discussed in this appendix is the Constant Elasticity of Substitution (CES) utility model which does, in fact, accommodate large, non-marginal energy efficiency and energy service price changes. The CES utility model underlies the substitution effect in this framework (see Section 2.5.2). Furthermore, the CES utility model is needed for the example energy efficiency upgrades (EEUs) in Heun et al. (2025), which have large, non-marginal percentage increases in energy efficiency.

In addition to the substitution effect, the income effect requires income elasticities to describe consumer behavior. Elasticities for both the substitution effect and the income effect are discussed below, after we lay out the CPE and CES utility models.

Before proceeding with the utility models and elasticities, we note briefly that the rate of other goods consumption ( ${\overset{\cdot }{q}}_{g}$ ) is not known independently from the prices of other goods ( $p_{g}$ ). With the assumption that the prices of other goods do not change across rebound effects (i.e., $p_{g}$ is exogenous), the ratio of other goods consumption is equal to the ratio of other goods spending, such that

(156)
q·gq·g°=C·g/pgC·g°/pg=C·gC·g°

at all rebound stages. (See Appendix E for details.)

#### C.1. Utility Models for the Substitution Effect

A utility model gives the ratio of energy service consumption rate and other goods consumption rates across the substitution effect ( ${\hat{\overset{\cdot }{q}}}_{s}/{\overset{\cdot }{q}}_{s}^{*}$ and ${\hat{\overset{\cdot }{q}}}_{g}/{\overset{\cdot }{q}}_{g}^{*}$ , respectively). In so doing, utility models quantify the decrease in other goods consumption ( ${\hat{\overset{\cdot }{q}}}_{g}/{\overset{\cdot }{q}}_{g}^{*}\;<\;1$ ) caused by the increase of energy service consumption ( ${\hat{\overset{\cdot }{q}}}_{s}/{\overset{\cdot }{q}}_{s}^{*}\;>\;1$ ) resulting from the decrease of the energy service price ( $p_{s}^{*}\;<\;p_{s}^{{}^{\circ}}$ ) under the constraint of constant device user utility. Across the substitution effect, the utility increase of the larger energy service consumption rate must be exactly offset by the utility decrease of the smaller other goods consumption rate.

##### C.1.1. Constant price elasticity (CPE) utility model

The constant price elasticity (CPE) utility model is given by equations (125) and (129). The equations for the approximate utility model are repeated here for convenience.

(125)
$$\frac{{\hat{\overset{\cdot }{q}}}_{s}}{{\overset{\cdot }{q}}_{s}^{{}^{\circ}}}={\left(\frac{\eta^{*}}{\eta^{{}^{\circ}}}\right)}^{-\varepsilon_{{\overset{\cdot }{q}}_{s},p_{s}}}$$

(129)
$$\frac{{\hat{\overset{\cdot }{C}}}_{g}}{{\overset{\cdot }{C}}_{g}^{*}}=1-\frac{p_{E}{\overset{\cdot }{q}}_{s}^{*}}{\eta^{*}{\overset{\cdot }{C}}_{g}^{*}}\left[{\left(\frac{\eta^{*}}{\eta^{{}^{\circ}}}\right)}^{-\varepsilon_{{\overset{\cdot }{q}}_{s},p_{s}}}-1\right]$$

##### C.1.2. CES utility model

The CES utility model is given by equation (16). Here, its derivation is shown. Throughout the derivation, references to Heun et al. (2025) are provided for visual representations of several important concepts. Those concepts (e.g., equilibrium tangency requirements) are best visualized in rebound planes that are introduced in Section 2.2 of Heun et al. (2025).

The CES utility model is normalized by (indexed to) conditions prior to emplacement:

(157)
$$\frac{\overset{\cdot }{u}}{{\overset{\cdot }{u}}^{{}^{\circ}}}={\left[a{\left(\frac{{\overset{\cdot }{q}}_{s}}{{\overset{\cdot }{q}}_{s}^{{}^{\circ}}}\right)}^{\rho }+\left(1-a\right){\left(\frac{{\overset{\cdot }{q}}_{g}}{{\overset{\cdot }{q}}_{g}^{{}^{\circ}}}\right)}^{\rho }\right]}^{\left(1/\rho \right)},$$

where ρ≡(σ−1)/σ , a is a share parameter (determined below), and σ is the elasticity of substitution between the normalized consumption rate of the energy service ( ${\overset{\cdot }{q}}_{s}$ ) and the normalized consumption rate of other goods ( ${\overset{\cdot }{q}}_{g}$ ).^26^ By definition, σ is assumed constant such that $\sigma^{{}^{\circ}}=\sigma^{*}=\hat{\sigma }=\bar{\sigma }=\tilde{\sigma }=\sigma$ .

With the assumption of exogenous other goods prices in equation (156), we find

(158)
$$\frac{\overset{\cdot }{u}}{{\overset{\cdot }{u}}^{{}^{\circ}}}={\left[a{\left(\frac{{\overset{\cdot }{q}}_{s}}{{\overset{\cdot }{q}}_{s}^{{}^{\circ}}}\right)}^{\rho }+\left(1-a\right){\left(\frac{{\overset{\cdot }{C}}_{g}}{{\overset{\cdot }{C}}_{g}^{{}^{\circ}}}\right)}^{\rho }\right]}^{\left(1/\rho \right)}.$$

Equation (158) is the functional form of the CES utility model, whose share parameter ( a ) is yet to be determined. The correct expression for the share parameter ( a ) is found from the equilibrium requirement, namely that the expenditure curve is tangent to the indifference curve in the ${\overset{\cdot }{C}}_{g}/{\overset{\cdot }{C}}_{g}^{{}^{\circ}}$ vs. ${\overset{\cdot }{q}}_{s}/{\overset{\cdot }{q}}_{s}^{{}^{\circ}}$ plane (the “consumption plane” in Heun et al. (2025)) prior to the EEU. For example, the °−−° line is tangent to the constant-utility indifference curve $i^{{}^{\circ}}{-i}^{{}^{\circ}}$ at point ° in Figures 4 and 7 of Heun et al. (2025).

To find the slope at any point on the indifference curve ( $i^{{}^{\circ}}-\;\;\;i^{{}^{\circ}}$ in Figures 4 and 7 of Heun et al. (2025)), equation (158) can be rearranged to give the normalized consumption rate of other goods ( ${\overset{\cdot }{C}}_{g}/{\overset{\cdot }{C}}_{g}^{{}^{\circ}}$ ) as a function of the normalized consumption rate of the energy service ( ${\overset{\cdot }{q}}_{s}/{\overset{\cdot }{q}}_{s}^{{}^{\circ}}$ ) and the normalized utility rate ( $\overset{\cdot }{u}/{\overset{\cdot }{u}}^{{}^{\circ}}$ ):

(159)
$$\frac{{\overset{\cdot }{C}}_{g}}{{\overset{\cdot }{C}}_{g}^{{}^{\circ}}}={\left[\frac{1}{1-a}{\left(\frac{\overset{\cdot }{u}}{{\overset{\cdot }{u}}^{{}^{\circ}}}\right)}^{\rho }-\frac{a}{1-a}{\left(\frac{\overset{\cdot }{q}}{{\overset{\cdot }{q}}_{s}^{{}^{\circ}}}\right)}^{\rho }\right]}^{\left(1/\rho \right)}\;,$$

a form convenient for drawing constant utility rate ( $\overset{\cdot }{u}/{\overset{\cdot }{u}}^{{}^{\circ}}$ ) indifference curves on a graph of ${\overset{\cdot }{C}}_{g}/{\overset{\cdot }{C}}_{g}^{{}^{\circ}}$ vs. ${\overset{\cdot }{q}}_{s}/{\overset{\cdot }{q}}_{s}^{{}^{\circ}}$ (the consumption plane of Figures 4 and 7 in Heun et al. (2025)). In the consumption plane, the slope of an indifference curve is found by taking the first partial derivative of ${\overset{\cdot }{C}}_{g}/{\overset{\cdot }{C}}_{g}^{{}^{\circ}}$ with respect to ${\overset{\cdot }{q}}_{s}/{\overset{\cdot }{q}}_{s}^{{}^{\circ}}$ , starting from equation (159) and using the chain rule repeatedly. The result is

(160)
$$\begin{array}{c} \frac{\partial \left({\overset{\cdot }{C}}_{g}/{\overset{\cdot }{C}}_{g}^{{}^{\circ}}\right)}{\partial \left({\overset{\cdot }{q}}_{s}/{\overset{\cdot }{q}}_{s}^{{}^{\circ}}\right)}=-\frac{a}{1-a}{\left(\frac{{\overset{\cdot }{q}}_{s}}{{\overset{\cdot }{q}}_{s}^{{}^{\circ}}}\right)}^{\left(\rho -1\right)}\;{\left[\left(\frac{1}{1-a}\right){\left(\frac{\overset{\cdot }{u}}{{\overset{\cdot }{u}}^{{}^{\circ}}}\right)}^{\rho }-\left(\frac{a}{1-a}\right){\left(\frac{\overset{\cdot }{q}}{{\overset{\cdot }{q}}_{s}^{{}^{\circ}}}\right)}^{\rho }\right]}^{\left(1-\rho \right)/\rho }. \end{array}$$

The budget constraint is the starting point for finding the slope of an expenditure line in the consumption plane. (Example expenditure lines include the °−−°, *−−*, ∧−−∧ and −−- lines in Figures 4 and 7 of Heun et al. (2025).) The following equation is a generic version of equations (78), (80), (93), and (102) with $p_{s}{\overset{\cdot }{q}}_{s}$ substituted for $p_{E}{\overset{\cdot }{E}}_{s}$ .

(161)
$$\overset{\cdot }{M}=p_{s}{\overset{\cdot }{q}}_{s}+\tau_{\alpha }{\overset{\cdot }{C}}_{\mathrm{cap}}+{\overset{\cdot }{C}}_{\mathrm{OMd}}+{\overset{\cdot }{C}}_{g}+\overset{\cdot }{N}$$

In a manner similar to derivations in Appendix B.3.1 of Heun et al. (2025), we solve for ${\overset{\cdot }{C}}_{g}$ and judiciously multiply by ${\overset{\cdot }{C}}_{g}^{{}^{\circ}}/{\overset{\cdot }{C}}_{g}^{{}^{\circ}}$ and ${\overset{\cdot }{q}}_{s}^{{}^{\circ}}/{\overset{\cdot }{q}}_{s}^{{}^{\circ}}$ to obtain

(162)
$$\frac{{\overset{\cdot }{C}}_{g}}{{\overset{\cdot }{C}}_{g}^{{}^{\circ}}}{\overset{\cdot }{C}}_{g}^{{}^{\circ}}=-p_{s}\frac{{\overset{\cdot }{q}}_{s}}{{\overset{\cdot }{q}}_{s}^{{}^{\circ}}}{\overset{\cdot }{q}}_{s}^{{}^{\circ}}+\overset{\cdot }{M}-\tau_{\alpha }{\overset{\cdot }{C}}_{\mathrm{cap}}-{\overset{\cdot }{C}}_{\mathrm{OMd}}-\overset{\cdot }{N}.$$

Solving for ${\overset{\cdot }{C}}_{g}/{\overset{\cdot }{C}}_{g}^{{}^{\circ}}$ and rearranging gives

(163)
$$\frac{{\overset{\cdot }{C}}_{g}}{{\overset{\cdot }{C}}_{g}^{{}^{\circ}}}=-\frac{p_{s}{\overset{\cdot }{q}}_{s}^{{}^{\circ}}}{{\overset{\cdot }{C}}_{g}^{{}^{\circ}}}\left(\frac{{\overset{\cdot }{q}}_{s}}{{\overset{\cdot }{q}}_{s}^{{}^{\circ}}}\right)+\frac{1}{{\overset{\cdot }{C}}_{g}^{{}^{\circ}}}\left(\overset{\cdot }{M}-\tau_{\alpha }{\overset{\cdot }{C}}_{\mathrm{cap}}-{\overset{\cdot }{C}}_{\mathrm{OMd}}-\overset{\cdot }{N}\right),$$

from which the slope of the indifference curve in the consumption plane is taken by inspection to be

(164)
$$\frac{\partial \left({\overset{\cdot }{C}}_{g}/{\overset{\cdot }{C}}_{g}^{{}^{\circ}}\right)}{\partial \left({\overset{\cdot }{q}}_{s}/{\overset{\cdot }{q}}_{s}^{{}^{\circ}}\right)}=-\frac{p_{s}{\overset{\cdot }{q}}_{s}^{{}^{\circ}}}{{\overset{\cdot }{C}}_{g}^{{}^{\circ}}}.$$

At any equilibrium point, the expenditure line must be tangent to its indifference curve, or, as economists say, the ratio of prices must be equal to the marginal rate of substitution. Applying the tangency requirement before emplacement enables solving for the correct expression for a , the share parameter in the CES utility model. Setting the slope of the expenditure line (equation (164)) equal to the slope of the indifference curve (equation (160)) gives

(165)
$$-\frac{p_{s}{\overset{\cdot }{q}}_{s}^{{}^{\circ}}}{{\overset{\cdot }{C}}_{g}^{{}^{\circ}}}=-\frac{a}{1-a}{\left(\frac{{\overset{\cdot }{q}}_{s}}{{\overset{\cdot }{q}}_{s}^{{}^{\circ}}}\right)}^{\left(\rho -1\right)}\;{\left[\left(\frac{1}{1-a}\right){\left(\frac{\overset{\cdot }{u}}{{\overset{\cdot }{u}}^{{}^{\circ}}}\right)}^{\rho }-\left(\frac{a}{1-a}\right){\left(\frac{\overset{\cdot }{q}}{{\overset{\cdot }{q}}_{s}^{{}^{\circ}}}\right)}^{\rho }\right]}^{\left(1-\rho \right)/\rho }.$$

For the equilibrium point prior to emplacement (point ° in Figures 4 and 7 of Heun et al. (2025)), ${\overset{\cdot }{q}}_{s}/{\overset{\cdot }{q}}_{s}^{{}^{\circ}}=1$ , $\overset{\cdot }{u}/{\overset{\cdot }{u}}^{{}^{\circ}}=1$ , and $p_{s}=p_{s}^{{}^{\circ}}$ , which reduces equation (165) to

(166)
$$-\frac{p_{s}^{{}^{\circ}}{\overset{\cdot }{q}}_{s}^{{}^{\circ}}}{{\overset{\cdot }{C}}_{g}^{{}^{\circ}}}=-\frac{a}{1-a}{\left(1\right)}^{\left(\rho -1\right)}{\left[\left(\frac{1}{1-a}\right){\left(1\right)}^{\rho }-\left(\frac{a}{1-a}\right){\left(1\right)}^{\rho }\right]}^{\left(1-\rho \right)/\rho }.$$

Simplifying gives

(167)
$$\frac{p_{s}^{{}^{\circ}}{\overset{\cdot }{q}}_{s}^{{}^{\circ}}}{{\overset{\cdot }{C}}_{g}^{{}^{\circ}}}=\frac{a}{1-a}.$$

Recognizing that $p_{s}^{{}^{\circ}}{\overset{\cdot }{q}}_{s}^{{}^{\circ}}={\overset{\cdot }{C}}_{s}^{{}^{\circ}}$ and solving for a gives

(168)
$$a=\frac{{\overset{\cdot }{C}}_{s}^{{}^{\circ}}}{{\overset{\cdot }{C}}_{s}^{{}^{\circ}}+{\overset{\cdot }{C}}_{g}^{{}^{\circ}}}\;,$$

which is called $f_{{\overset{\cdot }{C}}_{s}}^{{}^{\circ}}$ , the share of energy service expenditure ( ${\overset{\cdot }{C}}_{s}^{{}^{\circ}}$ ) relative to the sum of energy service and other goods expenditures ( ${\overset{\cdot }{C}}_{s}^{{}^{\circ}}+{\overset{\cdot }{C}}_{g}^{{}^{\circ}}$ ) before emplacement of the EEU. Thus, the CES utility equation (equation (158)) becomes

(16)
$$\frac{\overset{\cdot }{u}}{{\overset{\cdot }{u}}^{{}^{\circ}}}={\left[f_{{\overset{\cdot }{C}}_{s}}^{{}^{\circ}}{\left(\frac{{\overset{\cdot }{q}}_{s}}{{\overset{\cdot }{q}}_{s}^{{}^{\circ}}}\right)}^{\rho }+\left(1-f_{{\overset{\cdot }{C}}_{s}}^{{}^{\circ}}\right){\left(\frac{{\overset{\cdot }{C}}_{g}}{{\overset{\cdot }{C}}_{g}^{{}^{\circ}}}\right)}^{\rho }\right]}^{\left(1/\rho \right)}\;,$$

with

(169)
$$f_{{\overset{\cdot }{C}}_{s}}^{{}^{\circ}}\;\equiv \;\frac{{\overset{\cdot }{C}}_{s}^{{}^{\circ}}}{{\overset{\cdot }{C}}_{s}^{{}^{\circ}}+{\overset{\cdot }{C}}_{g}^{{}^{\circ}}}.$$

#### C.2. Elasticities for the Substitution Effect

Calculating the change in consumer preferences across the substitution effect requires a utility model, two of which are described in the section above: the constant price elasticity (CPE) model and the constant elasticity of substitution (CES) model. Within those utility models, price ( ε ) and substitution ( σ ) elasticities describe consumer preferences.

Own and cross price elasticities describe consumer preferences for consumption of the energy service ( ${\overset{\cdot }{q}}_{s}$ ) and other goods ( ${\overset{\cdot }{q}}_{g}$ ) as the price of the energy service ( $p_{s}$ ) changes due to the EEU. Thus, there are four price elasticities: (i) the uncompensated own price elasticity of energy service consumption ( $\varepsilon_{{\overset{\cdot }{q}}_{s},p_{s}}$ ), (ii) the uncompensated cross price elasticity of other goods consumption ( $\varepsilon_{{\overset{\cdot }{q}}_{g},p_{s}}$ ), (iii) the compensated own price elasticity of energy service consumption ( $\varepsilon_{{\overset{\cdot }{q}}_{s},p_{s},c}$ ), and (iv) the compensated cross price elasticity of other goods consumption ( $\varepsilon_{{\overset{\cdot }{q}}_{g},p_{s},c}$ ).

The elasticity of substitution ( σ ) describes the willingness of consumers to substitute one good for another. In the context of rebound from an EEU, substitution is considered between consumption of the energy service ( ${\overset{\cdot }{q}}_{s}$ ) and comsumption of the basket of other goods ( ${\overset{\cdot }{q}}_{g}$ ).

##### C.2.1. Original, pre-EEU (°) elasticities

Economists use surveys, statistical data, and other means to estimate values for the uncompensated own price elasticity of energy service consumption $\left(\varepsilon_{{\overset{\cdot }{q}}_{s},p_{s}}^{{}^{\circ}}\right)$ prior to the EEU. With $\varepsilon_{{\overset{\cdot }{q}}_{s},p_{s}}^{{}^{\circ}}$ in hand, calculation of all other elasticities is possible.

*Elasticity of substitution* ( σ ). For the constant price elasticity (CPE) utility model, there is no analytical expression for the elasticity of substitution ( σ ) and values are most likely taken from estimation, if they are obtained at all. As we show in Tables 12 and 13 of Heun et al. (2025), not all rebounds are typically calculated, so not all elasticities are needed.

For the constant elasticity of substitution (CES) utility model, Gørtz (1977) shows that the elasticity of substitution prior to the EEU ( $\sigma^{{}^{\circ}}$ ) can be computed by

(170)
$$\sigma^{{}^{\circ}}=\frac{f_{{\overset{\cdot }{C}}_{s}}^{{}^{\circ}}+\varepsilon_{{\overset{\cdot }{q}}_{s},p_{s}}^{{}^{\circ}}}{f_{{\overset{\cdot }{C}}_{s}}^{{}^{\circ}}-1}.$$

Thus, the original elasticity of substitution ( $\sigma^{{}^{\circ}}$ ) can be determined from two pieces of readily available information: (i) the original uncompensated own price elasticity ( $\varepsilon_{{\overset{\cdot }{q}}_{s},p_{s}}^{{}^{\circ}}$ ) and (ii) the share of income spent on the energy service prior to the EEU ( $f_{{\overset{\cdot }{C}}_{s}}^{{}^{\circ}}$ from equation (169)). In the CES utility model, $\sigma^{{}^{\circ}}$ is assumed invariant and given the undecorated symbol σ to indicate that it applies across all rebound effects.

For the rest of the pre-EEU elasticities ( $\varepsilon_{{\overset{\cdot }{q}}_{s},p_{s}}^{{}^{\circ}}$ , $\varepsilon_{{\overset{\cdot }{q}}_{s},p_{s},c}^{{}^{\circ}}$ , and $\varepsilon_{{\overset{\cdot }{q}}_{g},\mathrm{ps},c}^{{}^{\circ}}$ ), there is no difference for the CPE utility model or the CES utility model.

*Uncompensated cross price elasticity* ( $\varepsilon_{{\overset{\cdot }{q}}_{s},p_{s}}^{{}^{\circ}}$ ). From Hicks and Allen (1934), we note that the pre-EEU uncompensated cross price elasticity ( $\varepsilon_{{\overset{\cdot }{q}}_{s},p_{s}}^{{}^{\circ}}$ ) can be expressed as

(171)
$$\varepsilon_{{\overset{\cdot }{q}}_{s},p_{s}}^{{}^{\circ}}=f_{{\overset{\cdot }{C}}_{s}}^{{}^{\circ}}\left(\sigma -\varepsilon_{{\overset{\cdot }{q}}_{g},\overset{\cdot }{M}}\right).$$

*Compensated own price elasticity* ( $\varepsilon_{{\overset{\cdot }{q}}_{s},p_{s},c}^{{}^{\circ}}$ ). An expression for the pre-EEU compensated own price elasticity ( $\varepsilon_{{\overset{\cdot }{q}}_{s},p_{s},c}^{{}^{\circ}}$ ) can be derived using the Slutsky equation, whereby the uncompensated own price elasticity of the energy service ( $\varepsilon_{{\overset{\cdot }{q}}_{s},p_{s}}^{{}^{\circ}}$ ) is decomposed into the compensated own price elasticity ( $\varepsilon_{{\overset{\cdot }{q}}_{s},p_{s},c}^{{}^{\circ}}$ ) and the income elasticity ( $\varepsilon_{{\overset{\cdot }{q}}_{s},\overset{\cdot }{M}}$ ) as follows:

(172)
$$\varepsilon_{{\overset{\cdot }{q}}_{s},p_{s}}^{{}^{\circ}}=\varepsilon_{{\overset{\cdot }{q}}_{s},p_{s},c}^{{}^{\circ}}-f_{{\overset{\cdot }{C}}_{s}}^{{}^{\circ}}\varepsilon_{{\overset{\cdot }{q}}_{s},\overset{\cdot }{M}}\;,$$

where $f_{{\overset{\cdot }{C}}_{s}}^{{}^{\circ}}$ is given by equation (169), and the income elasticity ( $\varepsilon_{{\overset{\cdot }{q}}_{s},\overset{\cdot }{M}}$ ) is given in Section C.3. Solving for the compensated price elasticity prior to the EEU ( $\varepsilon_{{\overset{\cdot }{q}}_{s},p_{s},c}^{{}^{\circ}}$ ) gives

(173)
$$\varepsilon_{{\overset{\cdot }{q}}_{s},p_{s},c}^{{}^{\circ}}=\varepsilon_{{\overset{\cdot }{q}}_{s},p_{s}}^{{}^{\circ}}+f_{{\overset{\cdot }{C}}_{s}}^{{}^{\circ}}\varepsilon_{{\overset{\cdot }{q}}_{s},\overset{\cdot }{M}}.$$

Compensated cross price elasticity ( $\varepsilon_{{\overset{\cdot }{q}}_{g},p_{s},c}^{{}^{\circ}}$ ). The cross price version of the Slutsky equation is the starting point for deriving the pre-EEU compensated cross price elasticity ( $\varepsilon_{{\overset{\cdot }{q}}_{g},p_{s},c}^{{}^{\circ}}$ ):

(174)
$$\varepsilon_{{\overset{\cdot }{q}}_{s},p_{s}}^{{}^{\circ}}=\varepsilon_{{\overset{\cdot }{q}}_{g},p_{s},c}^{{}^{\circ}}-f_{{\overset{\cdot }{C}}_{s}}^{{}^{\circ}}\varepsilon_{{\overset{\cdot }{q}}_{g},\overset{\cdot }{M}}.$$

The income elasticity of other goods consumption ( $\varepsilon_{{\overset{\cdot }{q}}_{g},\overset{\cdot }{M}}$ ) is given in Section C.3. Solving for $\varepsilon_{{\overset{\cdot }{q}}_{g},p_{s},c}^{{}^{\circ}}$ gives

(175)
$$\varepsilon_{{\overset{\cdot }{q}}_{g},p_{s},c}^{{}^{\circ}}=\varepsilon_{{\overset{\cdot }{q}}_{s},p_{s}}^{{}^{\circ}}+f_{{\overset{\cdot }{C}}_{s}}^{{}^{\circ}}\varepsilon_{{\overset{\cdot }{q}}_{g},\overset{\cdot }{M}}.$$

An alternative formulation can be derived by setting equation (171) equal to equation (174) to obtain

(176)
$$f_{{\overset{\cdot }{C}}_{s}}^{{}^{\circ}}\left(\sigma -\varepsilon_{{\overset{\cdot }{q}}_{g},\overset{\cdot }{M}}\right)=\varepsilon_{{\overset{\cdot }{q}}_{g},p_{s},c}^{{}^{\circ}}-f_{{\overset{\cdot }{C}}_{s}}^{{}^{\circ}}\varepsilon_{{\overset{\cdot }{q}}_{g},\overset{\cdot }{M}}.$$

Solving for $\varepsilon_{{\overset{\cdot }{q}}_{g},p_{s},c}^{{}^{\circ}}$ gives

(177)
$$\varepsilon_{{\overset{\cdot }{q}}_{g},p_{s},c}^{{}^{\circ}}=f_{{\overset{\cdot }{C}}_{s}}^{{}^{\circ}}\sigma .$$

Substituting σ from equation (170) gives

(178)
$$\varepsilon_{{\overset{\cdot }{q}}_{g},p_{s},c}^{{}^{\circ}}=\frac{f_{{\overset{\cdot }{C}}_{s}}^{{}^{\circ}}\left(f_{{\overset{\cdot }{C}}_{s}}^{{}^{\circ}}+\varepsilon_{{\overset{\cdot }{q}}_{s},p_{s}}^{{}^{\circ}}\right)}{f_{{\overset{\cdot }{C}}_{s}}^{{}^{\circ}}-1}.$$

Assuming a known value for the original uncompensated own price elasticity ( $\varepsilon_{{\overset{\cdot }{q}}_{s},p_{s}}^{{}^{\circ}}$ ), all other pre-EEU elasticities can be calculated from equations (170), (171), (173), and (175) or (178).

Note that the rebound framework in this paper uses the CES utility model and needs only the uncompensated own price elasticity ( $\varepsilon_{{\overset{\cdot }{q}}_{s},p_{s}}^{{}^{\circ}}$ ) and the derived elasticity of substitution ( σ ) to calculate rebound values. The other price elasticities ( $\varepsilon_{{\overset{\cdot }{q}}_{s},p_{s}}^{{}^{\circ}}$ , $\varepsilon_{{\overset{\cdot }{q}}_{s},p_{s},c}^{{}^{\circ}}$ , and $\varepsilon_{{\overset{\cdot }{q}}_{g},p_{s},c}^{{}^{\circ}}$ ) are not necessary for the model. However, they are helpful for elucidating results derived from the framework, a task left for Heun et al. (2025).

##### C.2.2. Post substitution effect (∧) elasticities

The stage after the substitution effect (∧) represents utility-maximizing behavior after the energy service price drop caused by the EEU and the compensating variation. Post-EEU, elasticities may be different from the original condition, because the consumption bundle has changed (due to a move along the indifference curve). This section derives expressions for elasticities at the ∧ stage. Elasticities at the ∧ stage are different for the CPE utility model and the CES utility model.

*CPE utility model*. By definition, the uncompensated own-price elasticity is assumed unchanged from their original values across the substitution effect in the constant price elasticity (CPE) utility model. Thus,

(179)
$$\varepsilon_{{\overset{\cdot }{q}}_{s},p_{s}}^{{}^{\circ}}={\hat{\varepsilon }}_{{\overset{\cdot }{q}}_{s},p_{s}}.$$

*CES utility model.* The CES utility model is rather different to the CPE model with respect to the behavior of elasticities across the substitution effect. In the CES utility model, price elasticities ( ε ) are different after the substitution effect (∧) compared to the original (°).

*Elasticity of substitution* ( σ ). Be definition, the elasticity of substitution ( σ ) is constant across the substitution effect for the CES utility model. Thus,

(180)
$$\sigma^{{}^{\circ}}=\hat{\sigma }.$$

Because the elasticity of substitution is unchanged, we refer to σ without decoration for the CES utility model. The constancy of σ means that the price elasticities ( ε ) will vary with the energy service price ( $p_{s}^{*}$ ) across the substitution effect.

*Compensated own price elasticity* ( ${\hat{\varepsilon }}_{{\overset{\cdot }{q}}_{s},p_{s},c}$ ). The compensated own price elasticity of energy service demand ( ${\hat{\varepsilon }}_{{\overset{\cdot }{q}}_{s},p_{s},c}$ ) gives the percentage change of the consumption rate of the energy service ( ${\overset{\cdot }{q}}_{s}$ ) across the substitution effect due to a unit percentage change in the energy service price ( $p_{s}$ ) resulting from the EEU under the constraint that utility is unchanged ( ${\overset{\cdot }{u}}^{*}=\hat{\overset{\cdot }{u}}$ ). In contrast to the CPE utility model above, the compensated own price elasticity of energy service demand ( ${\hat{\varepsilon }}_{{\overset{\cdot }{q}}_{s},p_{s},c}$ ) is not constant in the CES utility model. Rather, ${\hat{\varepsilon }}_{{\overset{\cdot }{q}}_{s},p_{s},c}$ is a function of the post-EEU energy service price ( $p_{s}^{*}$ ). The definition of ${\hat{\varepsilon }}_{{\overset{\cdot }{q}}_{s},p_{s},c}$ is

(181)
$${\hat{\varepsilon }}_{{\overset{\cdot }{q}}_{s},p_{s},c}\;\equiv \;\frac{p_{s}^{*}}{{\hat{\overset{\cdot }{q}}}_{s}}{\frac{\partial {\hat{\overset{\cdot }{q}}}_{s}}{\partial p_{s}^{*}}|}_{\overset{\cdot }{u}\;=\;{\overset{\cdot }{u}}^{*}\;=\;\hat{\overset{\cdot }{u}}}.$$

To find an expression for ${\hat{\varepsilon }}_{{\overset{\cdot }{q}}_{s},p_{s},c}$ for the CES utility function, we need to first find the partial derivative of the rate of energy service consumption ( ${\hat{\overset{\cdot }{q}}}_{s}$ ) with respect to the post-EEU energy service price ( $p_{s}^{*}$ ) at constant utility ( $\overset{\cdot }{u}={\overset{\cdot }{u}}^{*}=\hat{\overset{\cdot }{u}}$ ) across the substitution effect. This derivation of an expression for ${\hat{\varepsilon }}_{{\overset{\cdot }{q}}_{s},p_{s},c}$ for the CES utility model commences with equation (21), which was derived for constant utility across the substitution effect.

(21)
$$\frac{{\hat{\overset{\cdot }{q}}}_{s}}{{\overset{\cdot }{q}}_{s}^{{}^{\circ}}}={\left\{f_{{\overset{\cdot }{C}}_{s}}^{{}^{\circ}}+\left(1-f_{{\overset{\cdot }{C}}_{s}}^{{}^{\circ}}\right){\left[\left(\frac{1-f_{{\overset{\cdot }{C}}_{s}}^{{}^{\circ}}}{f_{{\overset{\cdot }{C}}_{s}}^{{}^{\circ}}}\right)\frac{p_{s}^{*}{\overset{\cdot }{q}}_{s}^{{}^{\circ}}}{{\overset{\cdot }{C}}_{g}^{{}^{\circ}}}\right]}^{\rho /\left(1-\rho \right)}\right\}}^{-1/\rho }$$

In equation (21), all terms on the right side except $p_{s}^{*}$ are constant for the purposes of the partial derivative. Finding the partial derivative of ${\hat{\overset{\cdot }{q}}}_{s}$ with respect to $p_{s}^{*}$ amounts to applying the chain rule repeatedly. To simplify the derivation, we can define the following constants

(182)
$$f\;\equiv \;f_{{\overset{\cdot }{C}}_{s}}^{{}^{\circ}}\;,$$

(183)
$$g\;\equiv \;1-f_{{\overset{\cdot }{C}}_{s}}^{{}^{\circ}}\;,$$

(184)
$$h\;\equiv \;\frac{{\overset{\cdot }{q}}_{s}^{{}^{\circ}}}{{\overset{\cdot }{C}}_{g}^{{}^{\circ}}}\;,$$

(185)
$$m_{s}\;\equiv \;\rho /\left(1-\rho \right)\;,$$

(186)
n≡−1/ρ,and

(187)
$$z\;\equiv \;\frac{g}{f}h=\frac{1-f_{{\overset{\cdot }{C}}_{s}}^{{}^{\circ}}}{f_{{\overset{\cdot }{C}}_{s}}^{{}^{\circ}}}\frac{{\overset{\cdot }{q}}_{s}^{{}^{\circ}}}{{\overset{\cdot }{C}}_{g}^{{}^{\circ}}}$$

and rearrange slightly to obtain

(188)
$${\hat{\overset{\cdot }{q}}}_{s}={\overset{\cdot }{q}}_{s}^{{}^{\circ}}{\left[f+g{\left({\mathrm{zp}}_{s}^{*}\right)}^{m_{s}}\right]}^{n}.$$

Taking the partial derivative of ${\hat{\overset{\cdot }{q}}}_{s}$ with respect to $p_{s}^{*}$ , via repeated application of the chain rule, gives

(189)
$$\frac{\partial {\hat{\overset{\cdot }{q}}}_{s}}{\partial p_{s}^{*}}={\overset{\cdot }{q}}_{s}^{{}^{\circ}}m_{s}\mathrm{ng}z^{m_{s}}{\left(p_{s}^{*}\right)}^{m_{s}-1}\left\{{\left[f+g{\left({\mathrm{zp}}_{s}^{*}\right)}^{m_{s}}\right]}^{n-1}\right\}.$$

Forming the elasticity via its definition (equation (181)) gives

(190)
ε^q·s,ps,c≡ps*q·^s∂q·^s∂ps*|u·=u·*=u·^=p~sq·s°[f+g(zps*)ms]nq·s°msngzms(ps*)ms−1{[f+g(zps*)ms]n−1}.

Cancelling terms and combining $p_{s}^{*}$ and $\left[f+g{\left({\mathrm{zp}}_{s}^{*}\right)}^{m_{s}}\right]$ terms with different exponents gives

(191)
$${\hat{\varepsilon }}_{{\overset{\cdot }{q}}_{s},p_{s},c}=\frac{m_{s}\mathrm{ng}{\left({\mathrm{zp}}_{s}^{*}\right)}^{m_{s}}}{f+g{\left({\mathrm{zp}}_{s}^{*}\right)}^{m_{s}}}.$$

Back-substituting the constants and simplifying where possible yields

(192)
$${\hat{\varepsilon }}_{{\overset{\cdot }{q}}_{s},p_{s},c}=-\frac{\frac{1}{1-\rho }\left(1-f_{{\overset{\cdot }{C}}_{s}}^{{}^{\circ}}\right){\left[\frac{1-f_{{\overset{\cdot }{C}}_{s}}^{{}^{\circ}}}{f_{{\overset{\cdot }{C}}_{s}}^{{}^{\circ}}}\frac{p_{s}^{*}{\overset{\cdot }{q}}_{s}^{{}^{\circ}}}{{\overset{\cdot }{C}}_{g}^{{}^{\circ}}}\right]}^{\rho /\left(1-\rho \right)}}{f_{{\overset{\cdot }{C}}_{s}}^{{}^{\circ}}+\left(1-f_{{\overset{\cdot }{C}}_{s}}^{{}^{\circ}}\right){\left[\frac{1-f_{{\overset{\cdot }{C}}_{s}}^{{}^{\circ}}}{f_{{\overset{\cdot }{C}}_{s}}^{{}^{\circ}}}\frac{p_{s}^{*}{\overset{\cdot }{q}}_{s}^{{}^{\circ}}}{{\overset{\cdot }{C}}_{g}^{{}^{\circ}}}\right]}^{\rho /\left(1-\rho \right)}}.$$

Equation (192) shows that the compensated energy service price elasticity of energy service consumption ( ${\hat{\varepsilon }}_{{\overset{\cdot }{q}}_{s},p_{s},c}$ ) under the CES utility model is a function of the energy service price after the EEU ( $p_{s}^{*}$ ). It is negative, as it should be, because all terms are positive, with ρ and $f_{{\overset{\cdot }{C}}_{s}}^{{}^{\circ}}$ being bounded above by 1.

Of interest is how the elasticity changes as $p_{s}^{*}$ changes. Taking the derivative of equation (191) and simplifying gives

(193)
$$\frac{\partial {\hat{\varepsilon }}_{{\overset{\cdot }{q}}_{s},p_{s},c}}{\partial p_{s}^{*}}=\frac{m_{s}^{2}\mathrm{ng}{\left({\mathrm{zp}}_{s}^{*}\right)}^{m_{s}}}{p_{s}^{*}{\left(f+g{\left({\mathrm{zp}}_{s}^{*}\right)}^{m_{s}}\right)}^{2}}.$$

All terms taken to their power are positive with the exception of n . For σ<1 , n is positive; for σ>1 , n is negative. Since we expect σ<1 (otherwise we have backfire rebound conditions), the derivative is positive: the compensated own price elasticity becomes less negative as $p_{s}^{*}$ increases.^27^ Since the share of income spent on the energy service declines for σ<1 , it is not immediately clear in which direction ${\hat{\varepsilon }}_{{\overset{\cdot }{q}}_{s},p_{s}}$ moves according to equation (171). See Figure C.8 in Appendix C.7 of Heun et al. (2025) for a graph of the sensitivity of price elasticities ( $\hat{\varepsilon }$ ) to energy service price ( $p_{s}^{*}$ ) for concrete examples.

*Compensated cross price elasticity* ( ${\hat{\varepsilon }}_{{\overset{\cdot }{q}}_{g},p_{s},c}$ ). The compensated cross price elasticity of other goods demand ( ${\hat{\varepsilon }}_{{\overset{\cdot }{q}}_{g},p_{s},c}$ ) gives the percentage change of the consumption rate of other goods ( ${\overset{\cdot }{q}}_{g}$ ) across the substitution effect due to a unit percentage change in the energy service price ( ${\tilde{p}}_{s}$ ) resulting from the EEU under the constraint that utility is unchanged ( ${\overset{\cdot }{u}}^{*}=\hat{\overset{\cdot }{u}}$ ). To find the compensated cross price elasticity of other goods consumption ( ${\hat{\varepsilon }}_{{\overset{\cdot }{q}}_{g},p_{s},c}$ ), we follow a similar procedure as for deriving the own price elasticity of energy service consumption ( ${\hat{\varepsilon }}_{{\overset{\cdot }{q}}_{s},p_{s},c}$ ), with two differences being (i) the elasticity definition and (ii) the equation from which the partial derivative is derived.

The first difference is the definition of the compensated cross price elasticity of other goods consumption ( ${\hat{\varepsilon }}_{{\overset{\cdot }{q}}_{g},p_{s},c}$ ).

(194)
$${\hat{\varepsilon }}_{{\overset{\cdot }{q}}_{g},p_{s},c}\;\equiv \;\frac{p_{s}^{*}}{{\hat{\overset{\cdot }{q}}}_{g}}{\frac{\partial {\hat{\overset{\cdot }{q}}}_{g}}{\partial p_{s}^{*}}|}_{\overset{\cdot }{u}\;=\;{\overset{\cdot }{u}}^{*}\;=\;\hat{\overset{\cdot }{u}}}$$

Again, we need to find the partial derivative of the rate of other goods consumption ( ${\overset{\cdot }{q}}_{g}$ ) with respect to the energy service price ( $p_{s}^{*}$ ) at constant utility ( ${\overset{\cdot }{u}}^{*}=\hat{\overset{\cdot }{u}}$ ) across the substitution effect. The second difference is the starting point for this derivation, equation (22) (instead of equation (21)).

(22)
$$\frac{{\hat{\overset{\cdot }{C}}}_{g}}{{\overset{\cdot }{C}}_{g}^{{}^{\circ}}}={\left(1+f_{{\overset{\cdot }{C}}_{s}}^{{}^{\circ}}\left\{{\left[\left(\frac{1-f_{{\overset{\cdot }{C}}_{s}}^{{}^{\circ}}}{f_{{\overset{\cdot }{C}}_{s}}^{{}^{\circ}}}\right)\frac{p_{s}^{*}{\overset{\cdot }{q}}_{s}^{{}^{\circ}}}{{\overset{\cdot }{C}}_{g}^{{}^{\circ}}}\right]}^{\rho /\left(\rho -1\right)}-1\right\}\right)}^{-1/\rho }.$$

In equation (22), all terms on the right side except $p_{s}^{*}$ are constant for the purposes of the partial derivative. So finding the derivative amounts to applying the chain rule repeatedly. To simplify the derivation, we can define

(195)
$$m_{g}\;\equiv \;\rho /\left(\rho -1\right)\;,$$

invoke the constancy of other prices ( $p_{g}^{{}^{\circ}}={\hat{p}}_{g}$ ) from Appendix E, and rearrange slightly to obtain

(196)
$${\hat{\overset{\cdot }{q}}}_{g}={\overset{\cdot }{q}}_{g}^{{}^{\circ}}{\left\{1+f\left[{\left({\mathrm{zp}}_{s}^{*}\right)}^{m_{g}}-1\right]\right\}}^{n}\;,$$

with f , n , and z being constants defined in the derivation of ${\hat{\varepsilon }}_{{\overset{\cdot }{q}}_{s},p_{s},c}$ above.

Taking the partial derivative of ${\hat{\overset{\cdot }{q}}}_{g}$ with respect to $p_{s}^{*}$ , via repeated application of the chain rule, gives

(197)
$$\frac{\partial {\hat{\overset{\cdot }{q}}}_{g}}{\partial p_{s}^{*}}={\overset{\cdot }{q}}_{g}^{{}^{\circ}}m_{g}\mathrm{nf}z^{m_{g}}{\left(p_{s}^{*}\right)}^{m_{g}-1}{\left\{1+\left[f{\left({\mathrm{zp}}_{s}^{*}\right)}^{m_{g}}-1\right]\right\}}^{n-1}.$$

Forming the elasticity via its definition (equation (194)) gives

(198)
ε^q·g,ps,c≡ps*q·^g∂q·^g∂ps*|u·=u·*=u·^=ps*q·g°{1+f[(zps*)mg−1]}nq·g°mgnfzmg(ps*)mg−1{1+f[(zps*)mg−1]}n−1.

Cancelling terms and combining ${\tilde{p}}_{s}$ and $\left\{1+f\left[{\left({\mathrm{zp}}_{s}^{*}\right)}^{m_{g}}-1\right]\right\}$ terms with different exponents gives

(199)
$${\hat{\varepsilon }}_{{\overset{\cdot }{q}}_{g},p_{s},c}=\frac{m_{g}\mathrm{nf}{\left({\mathrm{zp}}_{s}^{*}\right)}^{m_{g}}}{1+f\left[{\left({\mathrm{zp}}_{s}^{*}\right)}^{m_{g}}-1\right]}.$$

Back-substituting the constants and simplifying where possible yields

(200)
$${\hat{\varepsilon }}_{{\overset{\cdot }{q}}_{g},p_{s},c}=-\frac{\left(\frac{1}{\rho -1}\right)f_{{\overset{\cdot }{C}}_{s}}^{{}^{\circ}}{\left(\frac{1-f_{{\overset{\cdot }{C}}_{s}}^{{}^{\circ}}}{f_{{\overset{\cdot }{C}}_{s}}^{{}^{\circ}}}\frac{p_{s}^{*}{\overset{\cdot }{q}}_{s}^{{}^{\circ}}}{{\overset{\cdot }{C}}_{g}^{{}^{\circ}}}\right)}^{\rho /\left(\rho -1\right)}}{1+f_{{\overset{\cdot }{C}}_{s}}^{{}^{\circ}}\left[{\left(\frac{1-f_{{\overset{\cdot }{C}}_{s}}^{{}^{\circ}}}{f_{{\overset{\cdot }{C}}_{s}}^{{}^{\circ}}}\frac{p_{s}^{*}{\overset{\cdot }{q}}_{s}^{{}^{\circ}}}{{\overset{\cdot }{C}}_{g}^{{}^{\circ}}}\right)}^{\rho /\left(\rho -1\right)}-1\right]}.$$

Equation (200) shows that the compensated energy service price elasticity of other goods consumption ( ${\hat{\varepsilon }}_{{\overset{\cdot }{q}}_{g},p_{s},c}$ ) under the CES utility model is a function of the energy service price after the EEU ( $p_{s}^{*}$ ). It is positive, because all terms except $\frac{1}{\rho -1}$ are positive, with ρ and $f_{{\overset{\cdot }{C}}_{s}}^{{}^{\circ}}$ being bounded above by 1.

Of interest is how the elasticity changes as $p_{s}^{*}$ changes. Taking the derivative of 199 and simplifying gives

(201)
$$\frac{\partial {\hat{\varepsilon }}_{{\overset{\cdot }{q}}_{g},p_{s},c}}{\partial p_{s}^{*}}=\frac{m_{g}^{2}\mathrm{nf}{\left({\mathrm{zp}}_{s}^{*}\right)}^{m_{g}}}{p_{s}^{*}{\left(1+f\left[{\left({\mathrm{zp}}_{s}^{*}\right)}^{m_{g}}-1\right]\right)}^{2}}.$$

All terms taken to their power are positive with the exception of n , analogous to the derivative of the own price elasticity in equation 193. Thus, with σ<1 and n positive, the compensated cross price elasticity becomes more positive as $p_{s}^{*}$ increases.

See Figure C.8 of Appendix C.7 of Heun et al. (2025) for a graph of the sensitivity of price elasticities ( $\hat{\varepsilon }$ ) to energy service price ( $p_{s}^{*}$ ) for concrete examples.

*Uncompensated own price elasticity* ( ${\hat{\varepsilon }}_{{\overset{\cdot }{q}}_{s},p_{s}}$ ). After finding the compensated own price elasticity ( ${\hat{\varepsilon }}_{{\overset{\cdot }{q}}_{s},p_{s},c}$ ), the Slutsky equation can be used directly to find the uncompensated own price elasticity ( ${\hat{\varepsilon }}_{{\overset{\cdot }{q}}_{s},p_{s}}$ ) after the substitution effect for the CES utility model.

(202)
$${\hat{\varepsilon }}_{{\overset{\cdot }{q}}_{s},p_{s}}={\hat{\varepsilon }}_{{\overset{\cdot }{q}}_{s},p_{s},c}-{\hat{f}}_{{\overset{\cdot }{C}}_{s}}\varepsilon_{{\overset{\cdot }{q}}_{s},\overset{\cdot }{M}}$$

*Uncompensated cross price elasticity* ( ${\hat{\varepsilon }}_{{\overset{\cdot }{q}}_{g},p_{s}}$ ). The result from Hicks and Allen (1934) can be used to calculate the uncompensated cross price elasticity ( ${\hat{\varepsilon }}_{{\overset{\cdot }{q}}_{g},p_{s}}$ ) for the CES utility model.

(203)
$${\hat{\varepsilon }}_{{\overset{\cdot }{q}}_{g},p_{s}}={\hat{f}}_{{\overset{\cdot }{C}}_{s}}\left(\sigma -\varepsilon_{{\overset{\cdot }{q}}_{g},\overset{\cdot }{M}}\right).$$

#### C.3. Elasticities for the Income Effect ( $\varepsilon_{{\overset{\cdot }{q}}_{s},\overset{\cdot }{M}}$ and $\varepsilon_{{\overset{\cdot }{q}}_{g},\overset{\cdot }{M}}$ )

The income effect requires two elasticities to estimate the spending of net savings: the income elasticity of energy service consumption ( $\varepsilon_{{\overset{\cdot }{q}}_{s},\overset{\cdot }{M}}$ ) and the income elasticity of other goods consumption ( $\varepsilon_{{\overset{\cdot }{q}}_{g},\overset{\cdot }{M}}$ ). Due to the homotheticity assumption, both income elasticities are unitary. Thus,

(204)
$$\varepsilon_{{\overset{\cdot }{q}}_{s},\overset{\cdot }{M}}=1\;,$$

and

(205)
$$\varepsilon_{{\overset{\cdot }{q}}_{g},\overset{\cdot }{M}}=1.$$

### D. Proof: Income Preference Equations Satisfy the Budget Constraint

After the substitution effect, a rate of net savings is available ( $\hat{\overset{\cdot }{N}}$ ), all of which is spent on additional energy service ( $\Delta {\bar{\overset{\cdot }{q}}}_{s}$ , $\Delta {\bar{\overset{\cdot }{C}}}_{s}=p_{E}\Delta {\bar{\overset{\cdot }{E}}}_{s}$ ) or additional other goods ( $\Delta {\bar{\overset{\cdot }{q}}}_{g}$ , $\Delta {\bar{\overset{\cdot }{C}}}_{g}$ ). The income effect must satisfy the budget constraint such that net savings is zero afterward ( $\bar{\overset{\cdot }{N}}=0$ ). The budget constraint across the income effect is represented by equation (108):

(108)
$$\hat{\overset{\cdot }{N}}=p_{E}\Delta {\bar{\overset{\cdot }{E}}}_{s}+\Delta {\bar{\overset{\cdot }{C}}}_{g}.$$

The additional spending due to the income effect is given by income preference equations

(25)
q·¯sq·^s=(1+N·^M·^')εq·s,M·

and

(29)
q·¯gq·^g=(1+N·^M·^')εq·g,M·,

where

(26)
M·^'≡M·−τα*C·cap*−C·OMd*−N·^.

This appendix proves that the income preference equations (equations (25) and (29)) satisfy the budget constraint (equation (108)).

The first step in the proof is to convert the income preference equations to ${\overset{\cdot }{C}}_{s}^{{}^{\circ}}$ and ${\overset{\cdot }{C}}_{g}^{{}^{\circ}}$ ratios. For the energy service income preference equation (equation (25)), multiply numerator and denominator of the left-hand side by $p_{s}^{*}=p_{E}/\eta^{*}$ (equation (7)) to obtain ${\bar{\overset{\cdot }{C}}}_{s}/{\hat{\overset{\cdot }{C}}}_{s}$ . For the other goods income preference equation (equation (29)), multiply numerator and denominator of the left-hand side by $p_{g}$ to obtain ${\bar{\overset{\cdot }{C}}}_{g}/{\hat{\overset{\cdot }{C}}}_{g}$ . Then, invoke homotheticity to set $\varepsilon_{{\overset{\cdot }{q}}_{s},\overset{\cdot }{M}}=1$ and $\varepsilon_{{\overset{\cdot }{q}}_{g},\overset{\cdot }{M}}=1$ to obtain

(206)
C·¯sC·^s=1+N·^M·^'

and

(207)
C·¯gC·^g=1+N·^M·^'.

The second step in the proof is to obtain expressions for $\Delta {\bar{\overset{\cdot }{C}}}_{s}$ and $\Delta {\bar{\overset{\cdot }{C}}}_{g}$ . Multiply the income preference equations above by $\Delta {\hat{\overset{\cdot }{C}}}_{s}$ and $\Delta {\hat{\overset{\cdot }{C}}}_{g}$ , respectively. Then, subtract $\Delta {\hat{\overset{\cdot }{C}}}_{s}$ and $\Delta {\hat{\overset{\cdot }{C}}}_{g}$ , respectively, to obtain

(208)
ΔC·¯s=C·^sM·^'N·^

and

(209)
ΔC·¯g=C·^gM·^'N·^.

The above versions of the income preference equations can be substituted into the budget constraint (equation (108)) to obtain

(210)
N·^=?C·^sM·^'N·^+C·^gM·^'N·^.

If equality is demonstrated, the income preference equations satisfy the budget constraint. The remainder of the proof shows the equality of equation (210).

Dividing by $\hat{\overset{\cdot }{N}}$ and multiplying by M·^' gives

(211)
C·^s+C·^g=?M·^'.

Substituting equation (26) for M·^' gives

(212)
$${\hat{\overset{\cdot }{C}}}_{s}+{\hat{\overset{\cdot }{C}}}_{g}\overset{?}{=}\;\;\overset{\cdot }{M}-R_{\alpha }^{*}{\overset{\cdot }{C}}_{\mathrm{cap}}^{*}-{\overset{\cdot }{C}}_{\mathrm{OMd}}^{*}-\hat{\overset{\cdot }{N}}.$$

Substituting equation (93) for $\overset{\cdot }{M}$ gives

(213)
C·^s+C·^g=?pEE·^s+R^αC·^cap+C·^OMd+C·^g+N·^−Rα*C·cap*−C·OMd*−N·^.

Cancelling terms and recognizing that $R_{\alpha }^{*}{\overset{\cdot }{C}}_{\mathrm{cap}}^{*}={\hat{R}}_{\alpha }{\hat{\overset{\cdot }{C}}}_{\mathrm{cap}}$ , ${\overset{\cdot }{C}}_{\mathrm{OMd}}^{*}={\hat{\overset{\cdot }{C}}}_{\mathrm{OMd}}$ , and ${\hat{\overset{\cdot }{C}}}_{s}=p_{E}{\hat{\overset{\cdot }{E}}}_{s}$ gives

(214)
C·^s+C·^g=?C·^s+R^αC·^cap+C·^OMd+C·^g−R^αC·^cap−C·^OMd.

Cancelling terms gives

(215)
$${\hat{\overset{\cdot }{C}}}_{s}+{\hat{\overset{\cdot }{C}}}_{g}\overset{✓}{=}\;{\hat{\overset{\cdot }{C}}}_{s}+{\hat{\overset{\cdot }{C}}}_{g},$$

thereby completing the proof that the income preference equations (equations (25) and (29)) satisfy the budget constraint (equation (108)).

### E. Other Goods Expenditures and Constant $p_{g}$

This framework utilizes a partial equilibrium analysis (at the microeconomic level) in which we account for the change of the energy service price due to the EEU ( $p_{s}^{{}^{\circ}}\neq p_{s}^{*}$ ), but we do not track the effect of the EEU on prices of other goods. These assumptions have important implications for the relationship between the rate of consumption of other goods ( ${\overset{\cdot }{q}}_{g}$ ) and the rate of expenditure on other goods ( ${\overset{\cdot }{C}}_{g}$ ).

We assume a basket of other goods (besides the energy service) purchased in the economy, each ( i ) with its own price ( $p_{g,i}$ ) and rate of consumption ( ${\overset{\cdot }{q}}_{g,i}$ ), such that the average price of all other goods purchased in the economy prior to the EEU ( $p_{g}^{{}^{\circ}}$ ) is given by

(216)
$$p_{g}^{{}^{\circ}}=\frac{\sum_{i}p_{g,i}^{{}^{\circ}}q_{g,i}^{{}^{\circ}}}{\sum_{i}q_{g,i}^{{}^{\circ}}}.$$

Then, the expenditure rate of other purchases in the economy can be given as

(217)
$${\overset{\cdot }{C}}_{g}^{{}^{\circ}}=p_{g}^{{}^{\circ}}{\overset{\cdot }{q}}_{g}^{{}^{\circ}}$$

before the EEU and

(218)
$${\hat{\overset{\cdot }{C}}}_{g}={\hat{p}}_{g}{\hat{\overset{\cdot }{q}}}_{g}$$

after the substitution effect, for example.

We assume that any microeconomic effects (emplacement, substitution, or income) for a single device are not so large that they cause a measurable change in prices of other goods. Thus,

(219)
$$p_{g}^{{}^{\circ}}=p_{g}^{*}={\hat{p}}_{g}={\bar{p}}_{g}={\tilde{p}}_{g}=p_{g}.$$

In the partial equilibrium analysis, any two other goods prices can be equated across any rebound effect to obtain (for the example of the original conditions (°) and the post-substitution state (∧))

(220)
$$\frac{{\hat{\overset{\cdot }{C}}}_{g}}{{\overset{\cdot }{C}}_{g}^{{}^{\circ}}}=\frac{{\hat{\overset{\cdot }{q}}}_{g}}{{\overset{\cdot }{q}}_{g}^{{}^{\circ}}}.$$

Thus, a ratio of other goods expenditure rates is always equal to a ratio of other goods consumption rates.

### F. Energy Price Rebound

Energy price rebound ( $Re_{p_{E}}$ ) is caused by a reduction in energy price ( $p_{E}$ ) that can occur when widespread implementation of an energy efficiency upgrade (EEU) leads to an economy-wide reduction in energy demand. Reduced demand leads to the lower energy price ( $p_{E}$ ). Conceptually, the demand schedule for energy, which associates each level of economy-wide energy demand with a price, shifts to the left. Consumers demand less energy at any given price of energy, as consumers can meet their needs with less energy than before thanks to the EEU. Then adjustment takes place along the unchanged energy supply schedule. Hence, the price elasticity of energy supply can be used to derive the new energy price. As a result, the device owner spends less on energy purchases to operate the upgraded device and all other devices that use the same energy type. For simplicity, we assume the device owner’s additional freed cash is spent on other goods and services with energy implications at the energy intensity of the economy ( $I_{E}$ ).

This appendix derives an expression for an energy price rebound (equation (36)) shown in Section 3.2. This derivation and our assessment of the magnitude of energy price rebound in Heun et al. (2025) illustrate the flexibility and extensibility of the framework presented in these papers.

The derivation begins with an equation for the new economy-wide demand for energy ( ${\bar{\overset{\cdot }{Q}}}_{E}$ ) after the EEU:

(221)
$${\bar{\overset{\cdot }{Q}}}_{E}={\overset{\cdot }{Q}}_{E}^{{}^{\circ}}-f_{\mathrm{EEU}}N_{\mathrm{dev}}{\overset{\cdot }{E}}_{s}^{{}^{\circ}}\left(1-\frac{{\bar{\overset{\cdot }{E}}}_{s}}{{\overset{\cdot }{E}}_{s}^{{}^{\circ}}}\right)\;,$$

where ${\overset{\cdot }{Q}}_{E}$ is the rate of economy-wide demand for energy in MJ/year, $f_{\mathrm{EEU}}$ is the fraction of devices upgraded across the economy (i.e., the penetration of the EEU), $N_{\mathrm{dev}}$ is the number of devices in service, and ${\overset{\cdot }{E}}_{s}$ is the rate of energy consumption by a single device in MJ/device · year. The decorations “°” and “−” have the usual meanings provided in Figure 1, namely that “°” indicates the original, pre-EEU device and “−” indicates conditions for the device owner after emplacement, substitution, and income adjustments. The ratio between new ( ${\bar{\overset{\cdot }{Q}}}_{E}$ ) and pre-EEU ( ${\overset{\cdot }{Q}}_{E}^{{}^{\circ}}$ ) energy demand is given by

(222)
$$\frac{{\bar{\overset{\cdot }{Q}}}_{E}}{{\overset{\cdot }{Q}}_{E}^{{}^{\circ}}}=\frac{{\overset{\cdot }{Q}}_{E}^{{}^{\circ}}-f_{\mathrm{EEU}}N_{\mathrm{dev}}{\overset{\cdot }{E}}_{s}^{{}^{\circ}}\left(1-\frac{{\bar{\overset{\cdot }{E}}}_{s}}{{\overset{\cdot }{E}}_{s}^{{}^{\circ}}}\right)}{{\overset{\cdot }{Q}}_{E}^{{}^{\circ}}}\;.$$

Simplifying gives

(223)
$$\frac{{\bar{\overset{\cdot }{Q}}}_{E}}{{\overset{\cdot }{Q}}_{E}^{{}^{\circ}}}=1-f_{\mathrm{EEU}}\frac{N_{\mathrm{dev}}{\overset{\cdot }{E}}_{s}^{{}^{\circ}}}{{\overset{\cdot }{Q}}_{E}^{{}^{\circ}}}\left(1-\frac{{\bar{\overset{\cdot }{E}}}_{s}}{{\overset{\cdot }{E}}_{s}^{{}^{\circ}}}\right)\;.$$

Note that the group $\frac{N_{\mathrm{dev}}{\overset{\cdot }{E}}_{s}^{{}^{\circ}}}{{\overset{\cdot }{Q}}_{E}^{{}^{\circ}}}$ is the original (pre-EEU) fraction of all energy production (of the kind used by the device) consumed by all such devices throughout the economy.

The relationship between energy price ( $p_{E}$ ) and economy-wide energy supply ( ${\overset{\cdot }{Q}}_{E}$ ) can be given by an elasticity relationship

(224)
$$\frac{{\bar{\overset{\cdot }{Q}}}_{E}}{{\overset{\cdot }{Q}}_{E}^{{}^{\circ}}}={\left(\frac{{\bar{p}}_{E}}{p_{E}^{{}^{\circ}}}\right)}^{\varepsilon_{{\overset{\cdot }{Q}}_{E},p_{E}}}\;,$$

where $\varepsilon_{{\overset{\cdot }{Q}}_{E},p_{E}}$ is the energy price ( $p_{e}$ ) elasticity of economy-wide energy supply ( ${\overset{\cdot }{Q}}_{E}$ ) and is expected to be positive. To assess the effect on price ( $p_{E}^{{}^{\circ}}\;>\;{\bar{p}}_{E}$ ) of demand reduction due to widespread adoption of the EEU ( ${\overset{\cdot }{Q}}_{E}^{{}^{\circ}}\;>\;{\bar{\overset{\cdot }{Q}}}_{E}$ ), we solve for $\frac{{\bar{p}}_{E}}{p_{E}^{{}^{\circ}}}$ to obtain

(225)
$$\frac{{\bar{p}}_{E}}{p_{E}^{{}^{\circ}}}={\left(\frac{{\bar{\overset{\cdot }{Q}}}_{E}}{{\overset{\cdot }{Q}}_{E}^{{}^{\circ}}}\right)}^{\frac{1}{\varepsilon_{{\overset{\cdot }{Q}}_{E},p_{E}}}}\;.$$

Substituting equation (223) gives

(226)
$$\frac{{\bar{p}}_{E}}{p_{E}^{{}^{\circ}}}={\left[1-f_{\mathrm{EEU}}\frac{N_{\mathrm{dev}}{\overset{\cdot }{E}}_{s}^{{}^{\circ}}}{{\overset{\cdot }{Q}}_{E}^{{}^{\circ}}}\left(1-\frac{{\bar{\overset{\cdot }{E}}}_{s}}{{\overset{\cdot }{E}}_{s}^{{}^{\circ}}}\right)\right]}^{\frac{1}{\varepsilon_{{\overset{\cdot }{Q}}_{E},p_{E}}}}\;.$$

The energy price reduction ( $p_{E}^{{}^{\circ}}\;>\;{\bar{p}}_{E}$ ) leads to additional freed cash ( ${\overset{\cdot }{G}}_{p_{E}}$ ) for the device owner at a rate of

(227)
$${\overset{\cdot }{G}}_{p_{E}}=\left[{\overset{\cdot }{E}}^{{}^{\circ}}-\left({\overset{\cdot }{E}}_{s}^{{}^{\circ}}-{\bar{\overset{\cdot }{E}}}_{s}\right)\right]\left(p_{E}^{{}^{\circ}}-{\bar{p}}_{E}\right)\;,$$

where ${\overset{\cdot }{E}}^{{}^{\circ}}$ is the rate at which the device owner consumes the final energy carrier that supplies the energy service (gasoline for a car and electricity for an electric lamp) prior to the EEU in all devices (the upgraded device and others), ( ${\overset{\cdot }{E}}_{s}^{{}^{\circ}}-{\bar{\overset{\cdot }{E}}}_{s}$ ) reduces ${\overset{\cdot }{E}}^{{}^{\circ}}$ by the energy savings after the income adjustment such that ${\overset{\cdot }{E}}^{{}^{\circ}}-\left({\overset{\cdot }{E}}_{s}^{{}^{\circ}}-{\bar{\overset{\cdot }{E}}}_{s}\right)$ is the total rate of energy consumption by all of the consumer’s devices after the income effect and the energy price adjustment, and $\left(p_{E}^{{}^{\circ}}-{\bar{p}}_{E}\right)$ is the energy price reduction caused by reduced demand for energy across the whole economy estimated by equation (226).

Rearrangement of terms gives

(228)
$${\overset{\cdot }{G}}_{p_{E}}=\left[{\overset{\cdot }{E}}^{{}^{\circ}}-\left({\overset{\cdot }{E}}_{s}^{{}^{\circ}}-{\bar{\overset{\cdot }{E}}}_{s}\right)\right]\left(1-\frac{{\bar{p}}_{E}}{p_{E}^{{}^{\circ}}}\right)p_{E}^{{}^{\circ}}\;,$$

into which equation (226) can be substituted easily.

The energy implications of spending the additional freed cash ( ${\overset{\cdot }{G}}_{p_{E}}$ ) on other goods and services is ${\overset{\cdot }{G}}_{p_{E}}I_{E}$ , another energy takeback rate. By equation (3), rebound associated with this energy price effect takeback can be written as

(36)
$$Re_{p_{E}}=\frac{{\overset{\cdot }{G}}_{p_{E}}I_{E}}{{\overset{\cdot }{S}}_{\mathrm{dev}}}\;,$$

as shown in Section 3.2, thus completing the derivation.
